# Examining the lived experience of dementia with Lewy bodies through qualitative research: A systematic review

**DOI:** 10.1002/alz.70217

**Published:** 2025-05-15

**Authors:** Jennifer R. Mammen, Jennifer G. Goldman, Mirinda Tyo, Yuge Xiao

**Affiliations:** ^1^ University of Massachusetts Dartmouth College of Nursing and Health Sciences Dartmouth Massachusetts USA; ^2^ JPG Enterprises LLC, Chicago, IL Barrow Neurological Institute Phoenix Arizona USA; ^3^ Michael J. Fox Foundation for Parkinson's Research New York New York USA

**Keywords:** caregiver, cognitive, dementia with Lewy bodies, impacts, Lewy body dementia, lived experience, meaningfulness, mild cognitive impairment, mixed methods, patient centered, qualitative, symptoms

## Abstract

**INTRODUCTION:**

Dementia with Lewy bodies (DLB) is inadequately diagnosed and treated, which negatively influences patient outcomes.

**METHODS:**

This systematic review evaluated qualitative studies of lived experiences from DLB patient and caregiver perspectives to identify gaps and define future research directions.

**RESULTS:**

The review included 27 studies. Most reported caregiver experiences (67%). Few focused solely on people with DLB.  Three themes emerged: (1) symptoms and impacts; (2) caregivers’ care challenges; and (3) needs and priorities for education, support, and research. Gaps in qualitative literature included small DLB sample sizes, inconsistent diagnostic criteria, variably reported characteristics, merged data across dementia types and stages, and under‐representation of informants and diverse groups.

**DISCUSSION:**

This review provides the first qualitative evidence synthesis in DLB, highlighting profound impacts of DLB symptoms, but also gaps in understanding direct experiences. Additional qualitative work regarding the lived experience in DLB is needed to inform clinical management and therapeutic development.

**Highlights:**

- Symptoms in dementia with Lewy bodies (DLB) vary greatly across individuals.
- Existing qualitative studies, though limited, show profound personal impact in DLB.
- Rigorous, systematic qualitative research in DLB symptom science is needed.
- Identifying what matters to people with DLB is essential for guiding treatment.

## 1 BACKGROUND

Lewy body dementia (LBD), which includes dementia with Lewy bodies (DLB) and Parkinson's disease dementia (PDD), is the second most common neurodegenerative dementia after Alzheimer's disease (AD).^1^, ^2^, ^3^ Symptoms of LBD are heterogeneous and multi‐faceted, affecting cognitive, neuropsychiatric, motor, autonomic, sleep, and other functions,^4^ and frequently leadin to increased disability, caregiver stress, health‐care cost, and other negative outcomes for the person living with LBD and their caregiver.^5^, ^6^, ^7^, ^8^, ^9^ To date, there are few symptomatic treatments specifically for LBD and no medications to halt or delay its progression; thus, advances in symptomatic and disease‐modifying therapeutics are greatly needed.^4^, ^10^, ^11^ While DLB and PDD overlap biologically and clinically, they differ in their temporal course regarding the relative onset of cognitive and motor symptoms and in some symptoms, as not all people with DLB experience motor parkinsonism.^12^ Additionally, compared to Parkinson's disease (PD) and AD, DLB progresses more rapidly, and the combined cognitive and motor symptom effects in DLB frequently generate even greater economic and caregiver burden.^13^, ^14^ Yet, despite the existence of DLB criteria for almost 30 years,^15^ DLB remains under‐recognized with missed or delayed diagnoses,^16^, ^17^ which negatively influences patient care and outcomes from diagnostic, prognostic, treatment, and research standpoints.

Understanding the symptoms and impacts (i.e., functional or psychosocial changes resulting from symptoms) of DLB is essential to increasing awareness of the condition, improving clinical diagnosis and patient care, and advancing therapeutics targeting symptoms that are meaningful to patients and families. Work in related fields (e.g., PD, AD) highlights the importance of consensus‐based conceptual models of patient experience. These models help align clinical trial outcome measures that prioritize what matters to patients with the needs of regulatory organizations and the patient community.^18^, ^19^, ^20^ Indeed, these concepts form a core element of US Food and Drug Administration Guidances that stress the need for patient‐focused drug development.^21^, ^22^ However, there is currently no clear consensus on what symptoms and impacts matter in the DLB lived experience as well as how symptoms and impacts change over time or with disease stage and whether patient and caregiver perspectives differ. With increasing emphasis on identifying people at the earliest stages of disease, whether by biomarkers or clinically by “prodromal” or mild symptoms, understanding “what matters” in early disease is essential for therapeutic development and disease‐modifying clinical trials.

Noting diagnostic and disease details will aid the utility of qualitative findings.Both qualitative and quantitative data are useful for identifying patient and caregiver lived experiences. Qualitative methodologies in particular capture unique and detailed aspects through open‐ended questions, a flexible structure, and narrative generation.^23^, ^24^ Over the years, qualitative research in DLB regarding symptoms, priorities, and experiences has gained interest and illustrates many unmet needs faced by patients and caregivers.^25^, ^26^, ^27^, ^28^, ^29^ Studies, however, vary in methodology, population (LBD, DLB, and/or PDD or PD; patient, caregiver, or both), sample size, and focus (e.g., symptom based, research priorities, end of life). To the best of our knowledge, a synthesis of qualitative evidence about the DLB lived experience has not been previously reported. Therefore, the purpose of this systematic review was to analyze qualitative and mixed‐methods research with any qualitative components in LBD focusing primarily on DLB, given the relative paucity of attention about bothersome and impactful symptoms compared to what has been previously studied in PD.^18^, ^30^, ^31^, ^32^ Our study aims were to: (1) identify published literature to date, (2) characterize and summarize these findings, (3) identify gap areas, and (4) define directions for future qualitative research to advance the field. This knowledge is necessary to provide patient‐centric clinical care, support development of valid outcomes assessments, and evaluate the extent to which DLB symptom patterns may overlap with PD.

**RESEARCH IN CONTEXT**

1. **Systematic reviews**: Following Joanna Briggs Institute Mixed Methods Review criteria, a structured review of literature from selected databases (e.g., PubMed) was conducted to identify primary qualitative studies examining lived experiences in dementia with Lewy bodies (DLB). Themes were identified and quality of evidence evaluated using GRAD‐CERQual criteria.
2. **Interpretation**: Qualitative research in DLB is currently limited in breadth, size, and scope. Few studies include robust and diverse DLB cohorts, direct patient reports, and uniform criteria and data collection. Available studies, however, highlight important symptoms and perspectives from patients and caregivers. Thematic areas emphasize symptoms and impacts, challenging experiences of caregivers, and needs for the field.
3. **Future directions**: As the first qualitative evidence synthesis assessing the lived experience in DLB, this study provides a framework by which future qualitative research can address the prevalence, bothersomeness, and symptom experiences in DLB. Understanding these perspectives in overt and prodromal DLB is essential to advance therapeutic development and clinical management.

## 2 METHODS

Joanna Briggs Institute (JBI) mixed methods review criteria were used for this systematic review of the literature.^33^, ^34^ Steps included defining: (1) the review question, (2) inclusion/exclusion criteria, (3) the search strategy, followed by (4) assessment of study methodology and quality, (5) data analysis, (6) synthesis, and (7) presentation of results. The purpose of the review was to synthesize published, peer‐reviewed qualitative or mixed‐methods (MM) studies conducted in DLB to understand the state of the science and try to answer the overarching research question: “What are the meaningful symptoms and impacts (i.e., functional or psychosocial changes resulting from symptoms) of DLB from the perspective of patients and caregivers of people with DLB?”[Fig alz70217-fig-0001]

### 2.1 Inclusion/exclusion criteria

To be included in the review, studies had to be: (1) qualitative or MM research, with (2) a substantive focus on the lived experience of DLB or LBD, (3) from the perspective of patients or family/caregivers, and (4) published in a peer‐reviewed journal. Qualitative and MM research approaches included interviews (phone, video, in person, clinical), focus groups, narrative chart reviews, or open response surveys. Both DLB and LBD terms were included in the search and article review to avoid missing potentially relevant articles, due to variable reporting practices and use of LBD as an umbrella term for DLB and PDD. Gray literature and case reports were excluded due to lack of generalizability and risk of bias.

### 2.2 Search strategy and study selection

**FIGURE 1 alz70217-fig-0001:**
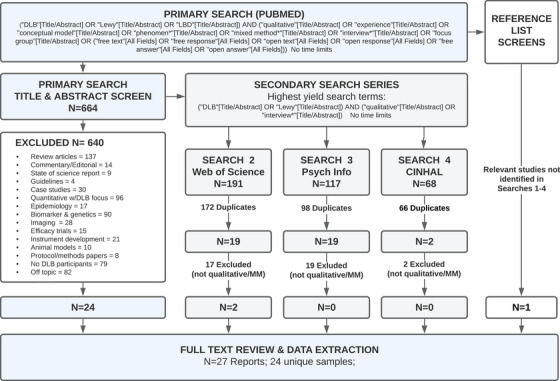
Search strategy and screening procedures. DLB, dementia with Lewy bodies; MM, mixed methods.

Search terms included all possible variations of DLB/LBD and common MM and qualitative terms (e.g., qualitative, interview*, lived‐experience, phenomen*, mixed‐method*, open response*, etc.). These terms were selected to capture any qualitative research conducted in LBD or DLB, without other limiters or time limit to avoid missing potentially relevant articles. Terms were searched first in PubMed database (August 2024). Titles and abstracts were manually screened for all references returned by J.M. with validation by J.G.G. and M.T. After the initial search and screen, a series of secondary searches was performed in PubMed to determine the highest yield search terms that most efficiently identified 100% of relevant articles identified in Search 1, while minimizing irrelevant results (e.g., “LBD” yielding “left brain disorder”). These final terms ([DLB *or* Lewy] and [Qualitative *or* Interview*]) were searched in Web of Science, PSYCHINFO, and CINHAL. Reference lists of DLB review articles retrieved in the original search were also reviewed to identify other potential sources. The search and screen flowchart are presented in Figure 1.

### 2.3 Data extraction and analysis

All studies that met inclusion criteria received a full text review (see audit trail of search and screen process in Supplement A in supporting information). Detailed data were extracted for methodological approach, study and sample characteristics (e.g., participants, demographics), diagnostic criteria and disease characteristics (e.g., duration), and key thematic findings for each study (Tables 1, 2A, 2B, 3). Dementia severity was recorded when reported in the study (e.g., as “moderate DLB”), but if not specified, was inferred from scores on standardized cognitive and functional measures (e.g., Clinical Dementia Rating scale). Thematic findings were then imported into Xmind mapping software and analyzed to determine common themes across studies. Themes were conceptually clustered to develop overarching categories of thematic findings, with attention to patient versus caregiver perspectives and specific experiences within these categories. Final thematic maps are presented in Supplements B and C in supporting information. Evidence from Tables 1 through 3 was then synthesized using GRADE‐CERQual to evaluate the quality of evidence for each thematic area. GRADE‐CERQual allows for systematic assessment of thematic evidence from qualitative studies^35^, ^36^, ^37^, ^38^, ^39^, ^40^ across four areas: (1) methodological limitations (Table 3), (2) coherence of findings, (3) adequacy of the data, and (4) relevance of the findings (Table 4). Methodological assessment included data collection approaches, participant characteristics, sampling biases, and factors affecting generalizability. Coherence was evaluated based on consistency or discrepancies in thematic findings between studies with similar topical focus. Adequacy was assessed based on quantity and depth of data to support findings. Relevance was assessed based on applicability to the lived experience of DLB from the patient or caregiver perspective. Basic descriptive statistics were computed for study characteristics.

**TABLE 1:** Study demographics.

Country of origin of study	Study *N* (%)
Australia	1 (3.7%)
France	1 (3.7%)
Japan	3 (11.1%)
Italy	1 (3.7%)
Sweden	1 (3.7%)
Netherlands	1 (3.7%)
UK	11 (40.7%)
USA	8 (29.6%)

Participant distribution	Participant *N* (%)
Caregiver total	**1688**
% Female	1287 (76.2%)
% Unknown	110 (6.5%)
Patient total	**319**
% Female	83 (26.0%)
% Unknown	32 (10.0%)

Race and ethnicity of all samples	Insufficient data^a^
Experiences explored in study	Study *N* (%)
Caregiver	11 (40.7%)
Patient	9 (33%)
Caregiver + patient	7 (25.9%)

Study data collection approach^b^	Study *N* (%)
Phone interviews	9 (33.3%)
Face to face interviews	6 (22.2%)
Focus groups	1 (3.7%)
Medical history/clinical interview	5 (18.5%)
Survey (online or in person)	7 (25.9%)
Retrospective chart review	1 (3.7%)

^a^ Only five studies reported race/ethnicity.

^b^ Some studies used multiple methods.

**TABLE 2A:** Comparison of aims, approaches, and findings from unique sample studies.

Author	CG^*^ (*n*)	Pt^*^ (*n*)	Aims	Approach	Results	Adequacy/Limitations
Matterson, Wilson‐Menzfeld, Olsen et al. (2024)^44^	7	‐	To investigate the phenomenology of cognitive fluctuations in DLB by understanding caregiver experiences	Recruited from parent study/specialty service Semi‐structured phone interview 1:1 with CG in moderate dementia Depth of interview not stated Asked: to describe frequency, duration and triggers of cognitive fluctuations, sleep experiences of Pt and own sleep experiences.	Total *N* = 7; 100% DLB; 100% CG 70% F; Race/ethnicity not reported Themes: (1) symptoms of cognitive fluctuations were inconsistent; (2) both Pt and CG had sleep disturbances due to DLB. Poor sleep quality was a trigger for worsening cognitive fluctuations. Excessive daytime sleepiness was common.	✓ is DLB specific study ✓ 2017 Consensus criteria for DLB ✓ MMSE; Severity – Moderate ✓ Dementia duration 4 yrs (2.3) ✓ Clinical Assessment Fluctuation scale ⊗ Small sample size ⊗ Cannot assess diversity ⊗ No Patient perspective ✓ Participant voice – CG quotes
Yumoto, Suwa (2024)^28^	17	13	To clarify the awareness of ‘‘changes in the daily lives of people with DLB’’ through frank descriptions by people with DLB and their families	Interview Pt with DLB and families Community dwelling Dyadic or individual interview Asked: changes and difficulties in daily life since DLB; symptoms experienced when changes or difficulties occurred.	Total N = 30; 100% DLB; 60% CG Race ethnicity, gender not explicit Most common symptoms were cognitive decline, visual hallucinations, RBSD, and parkinsonism. Difficulties were due to symptoms Pt was aware and unaware of. Themes: bodily discomfort (trouble moving/doing), daily obstacles (forgetting, communicating), psychological pain, isolation, frustration, depression.	✓ DLB specific study ✓ 2006 Consensus criteria for DLB ✓ Severity estimated MMSE and ADL score ✓ Disease duration: 4.3 years (3.8) ✓ Diversity: Japanese study ⊗ Limited CG demographics ✓ Participant voice – Pt + CG ✓ Some dyads/families ✓ Robust qualitative sample size ⊗ Does not address data saturation
Volkmer, Cross, Highton, et al. (2024)^81^	7	‐	To explore the experiences of aphasia in people living with or caring for somebody with FTD, LBD, PCA or YO‐AD regarding language and communication issues.	1 LBD focus group (7 CG) LBD duration 2‐8 yrs (early to late) 86% antemortem Asked: experiences of communication difficulties; how can speech language therapy help	Total *N* = 29; 24% LBD; 100% CG; 43% F, Race/ethnicity not reported Themes for LBD group: (1) communication difficulties a key problem, (2) feeling of isolation due to speech issues (worse for CG than for Pt), (3) doing anything that helps, (4) low access to speech and language therapy.	≅ Self‐report LBD (NOS PDD or DLB) ⊗ Diagnostic criteria not stated ⊗ Disease severity not reported ⊗ Inconsistent interview guide ⊗ Sampling bias: from RDS registry ⊗ Small LBD sample size ⊗ Cannot assess diversity ⊗ No patient perspective ✓ Participant voice – CG quotes
Harding, Rossi‐Harries, Gerritzen, et al. (2023)^29^	10	3	To explore COVID‐19 lockdown impact on people with young onset, non‐memory‐led, inherited dementias (FTD, PDD, PPA, DLB, AD) and their CG.	Online survey + open response Pt or CG recruited from RDS registry Combines dementia types Asked: (a) strategies to cope with lockdown, (b) positive aspects of lockdown (c) other experiences.	Total *N* = 208; 6% DLB; 88% CG Race/ethnicity/gender not reported Pt had worsening cognitive symptoms (70%), ability to do things (62%), well‐being (57%) and changes to medication (26%). Carers had less support (55%) which adversely affected their mental health.	✓ DLB subgroup analysis ⊗ Diagnostic criteria not stated ⊗ Disease severity not reported ⊗ Sampling bias: RDS registry ⊗ Low response rates – 208/1850 ⊗ Small DLB sample size ⊗ Minimal patient perspective ⊗ Cannot assess diversity ⊗ Participant voice – quotes
Gallop, Pham, Maclaine, et al. (2023)^82^	2	‐	To explore the impact of caring for people with PD, MSA, PAF or DLB who experience neurogenic orthostatic hypotension (nOH).	40‐70 min phone interviews CG perspective of symptoms recruited via specialist agency Asked: nOH symptoms + impact of nOH on caregiver	Total *N* = 20, 70% PD, 2% DLB; 100% CG, 70% F; Race/ethnicity not reported nOH impacted 8 areas for CG: physical health, wellbeing, daily activities, work, social and leisure, relationships, finances, and independence	⊗ Merges PD, MSA, DLB, PAF ⊗ No DLB subgroup analysis ⊗ DLB diagnostic criteria not stated ⊗ Disease severity not reported ⊗ Small sample, no Pt perspective ⊗ Cannot assess diversity ✓ Participant voice – quotes
Killen, Flynn, O'Brien et al. (2022)^55^	122	3	To explore the information and support needs of people with DLB + CG at diagnosis, to inform interventions to improve coping with stress and increase quality of life.	Retrospective analysis, older data Public survey from 2014 Hosted on Lewybody.org Self‐initiated, 80% UK participants Pt and CG informed survey items Asked: past support and information experiences, difficulties encountered that could benefit from information and support, and appropriate topics for future interventions.	Total *N* = 125, Unstated % DLB 98% CG 89% F; Race/ethnicity not reported Half reported no information or support following diagnosis. Most wanted more information was about hallucinations, cognitive fluctuation, psychiatric symptoms, sleep, memory, tremor and medications (>60% all). 90% wanted information to increase awareness, coping and data on what has worked for others.	⊗ Self‐report LBD or DLB – NOS ⊗ Self‐identified via website search ⊗ Diagnostic criteria not stated ⊗ Disease severity not reported ⊗ Data >10 years old ⊗ Sampling bias ⊗ No participant voice – no quotes ⊗ Quantitative data only ✓ One open question: Would you like to add any more information?
Stacy, Perazzo, Shatz, et al. (2022)^27^	20	0	To determine the needs, concerns, strategies, and advice of family caregivers of persons with LBD using an established needs and concerns framework	Recruited from cognitive disorders clinic LBD duration 0.5 to 5 years 45 min phone interviews Asked: typical day providing care, concerns, what helped, advice for newly diagnosed, needs for support program	Total *N* = 20; 100% LBD; 100% CG; 80% F; 90% white Concerns identified: difficulty getting diagnosis, what to expect, strategies to cope, need for support, symptom management, physical care, managing meds and appointments, mental health, respite, communication	✓ LBD (NOS PDD or DLB) ⊗ Diagnostic criteria not stated ⊗ Severity estimated from CDR – mild/mod ⊗ Wide LBD duration, Low diversity ⊗ No DLB subgroup analysis ✓ CG perspective and needs ⊗ No patient perspective ✓ Representative quotes
Kew, Juengst, Kelley, et al. (2022)^41^	8	0	To identify problems of English‐speaking CG of adults with ADRD (AD related dementias including AD and LBD) using the International Classification of Functioning Disability and Health (ICF)	Recruited from study on ADRD Used worksheet + focus group for data collection during problem‐solving training intervention Asked: current or anticipated problems, barriers to CG goals 11 prompts to explore common problem areas Problems discussed as group	Total *N* = 18; 44% LBD; 100% CG; 73% F; 70% white Focused on coverage of ICF: 402 concepts identified, 79% of which linked to ICF categories. Concerns were: (1) body structures, (2) body functions, (3) activities, (4) environmental factors, and (5) personal factors. Categories 1,2,3 most common. LBD CG faced more challenges than AD CG. Challenges not listed.	≅ LBD (NOS PDD or DLB) ⊗ Unclear diagnostic criteria ⊗ Disease severity mild to severe ✓ Dementia rated with FAST ⊗ No DLB subgroup analysis ⊗ Concepts/challenges not reported ✓ Diversity – 30% non‐white ⊗ Framework gap: < 80% concepts ⊗ Participant voice: Few quotes ⊗ No patient perspective
Brown, Aldridge, Pepper, et al. (2022)^42^	14	0	To explore family CG of people with LBD experiences of the LBD Admiral Nurse service	Phone or video interviews Asked: what help, support, & interventions Admiral Nurse offered, experiences; how useful services were; ways service could have been improved	Total *N* = 14; 100% LBD; 100% CG; 93% F; 100% white Themes: (1) CG valued specialty support program, which (2) enabled better management of challenges with practical guidance, (3) provided emotional benefits, and (4) helped CG feel supported.	≅ LBD (NOS PDD or DLB) ⊗ Diagnostic criteria not stated ⊗ Disease severity not stated ⊗ No DLB subgroup analysis ⊗ Lack of diversity ⊗ No patient perspective ⊗ Limited description of study methods
Yumoto and Suwa (2021)^45^	0	10	To explore visual hallucination related difficulties of people with DLB, coping methods, and relationship between symptoms/difficulties	60 min F2F interviews Participants with hallucinations + taking antidementia Rx Independent community dwelling	Total *N* = 10; 100% DLB; 100% Pt; 30% F; Race/ethnicity not reported Difficulties from hallucinations included feeling something strange is happening, discomfort, activity restrictions, inability to talk to family about it, and emotional distress. 70% did not use term hallucination.	✓ DLB specific study ✓ 2005 Consensus criteria for DLB ⊗ Severity mild to severe by ADL and MMSE ✓ Severity estimated MMSE and ADL score ✓ Diversity – Japanese study ✓ Includes Pt perspective ⊗ Small sample size
van de Beek, van Steenoven, van der Zande et al. (2020)^46^	0	73	To examine clinical characteristics, cognitive decline, and predictors for time to dementia in MCI‐LB vs. MCI‐AD	Amsterdam Dementia Cohort Semi‐structured medical history Clinical assessment with standardized instruments Asked: NPI, Disability Assessment for Dementia, Zarit, standardized tests. No qualitative interview	Total *N* = 197; 37% MCI‐LB; 100% Pt LB group 12% F; Race not reported Parkinsonism was the most frequent core feature in MCI‐LB (69%). LB fared worse on neuropsychiatric symptoms, non‐memory cognitive domains, and CG burden. LB group declined faster on attention	✓ 2017 Consensus criteria for MCI‐LB ✓ Severity = MCI‐LB; MMSE ≥25 ✓ Reports symptom frequencies ⊗ No open interview ⊗ No true qualitative data ⊗ No participant voice – no quotes ≅ Validated instruments only
Cagnin, Di Lorenzo, Marra et al. (2020)^60^	343	0	To investigate changes in behavioral and psychological symptoms of dementia (BPSD) during COVID‐19 quarantine in patients with various brain diseases leading to dementia and effects on CG	2020 nation‐wide phone survey Included CG of patient with dementia (AD, FTD, VD, DLB) Excluded MCI, psychiatric disorders Asked: patients’ BPSD changes (irritability, apathy, agitation, anxiety, depression, sleep disturbances, aggressiveness, wandering, appetite change, hallucinations, and delusions) and caregivers’ psychological symptoms	Total *N* = 4,913; 7% DLB; 100% CG DLB group = 66% F; Race not reported 59.6% had worsening or onset of new BPSD, which was higher for DLB (63.8%). DLB Pt with BPSD had > neuropsychiatric symptoms vs. other groups. Worsening was most frequently reported for: irritability (40.2%), apathy (34.5%), agitation (30.7%), anxiety (29%), depression (25.1%), sleep disorder (24%), delusions and hallucinations (10%). BPSD severity was similar across diseases.	⊗ Diagnostic criteria not stated ✓ Severity estimated CDRS moderate‐severe ✓ Disease duration 4.5 ± 3 ✓ DLB Subgroup analysis ⊗ Highly contextual –COVID‐19 lockdown ⊗ Recruiting not described ⊗ Limited demographics ⊗ Cannot assess diversity ⊗ No participant voice – no quotes ⊗ No patient perspective ⊗ No qualitative data ✓ Frequencies of symptoms
Coindreau, Chesnel, Babany et al. (2020)^53^	0	19	To explore Lower Urinary Tract Symptoms (LUTS) in people with LBD	Retrospective narrative chart review Neurology clinic in France Assessed: Clinical data on urinary frequency, urgency, incontinence, dysuria, fecal incontinence, sexual disorders, urinary retention. No interview with participant	Total *N* = 19; 100% DLB; 100% Pt 37% F; Race/ethnicity not reported 100% had overactive bladder, stress incontinence (94%), detrusor overactivity (93%), bladder pressure elevation (79%), voiding difficulties (16%), constipation (92%), and fecal incontinence (44%)	✓ DLB specific study ⊗ Diagnostic criteria not stated ⊗ Disease severity not reported ✓ Clinical data on symptoms ⊗ No participant voice ⊗ No patient perspective ⊗ No demographics ⊗ Cannot assess diversity ⊗ No qualitative data
Larsson, Holmbom‐Larsen, Torisson, et al. (2019)^54^	0	5	To explore the subjective experience of living with DLB from the patient perspective and explore feasibility of including Pt perspectives	In‐depth F2F interviews 60‐130 min w/ community dwelling Pt From memory clinic in Sweden DLB 1.5‐7 years; mild/moderate No structured interview guide Asked: “Tell me about yourself;” good day/bad day, symptoms, functional impacts, activities, QoL, what would make life better	Total *N* = 5; 100% DLB; 100% Pt; 0% F; Race/ethnicity not reported Themes focus on: (1) disease impact, i.e., symptom experience and restricted activities; (2) self‐perception and coping strategies; (3) importance of others. Patients reported wide range of symptoms and prioritized physical complaints over cognitive, in contrast to CG perspective in other studies.	✓ DLB specific study ✓ 2005 DLB consensus criteria ✓ MMSE ⊗ Very small sample ⊗ Lack of diversity ✓ Lived experience of DLB ✓ Includes Pt perspective ✓ Patient driven discussion ⊗ No symptom frequencies ✓ Participant voice – quotes
Vatter, Mcdonald, Stanmore, et al. (2018)^43^	12	0	To explore the changes in intimate relationships in PD‐related dementia (PD‐MCI, PDD, DLB), as perceived by spouses providing care to partners	CG Recruited from INVEST study CG duration 1‐10 years F2F interviews, 30‐97 minutes Interview guide informed by the Personal Assessment of Intimacy in Relationships (PAIR) scale Asked: relationship satisfaction and experiences	Total *N* = 12; 100% LBD; 25% DLB; 100% CG; 100% F; 100% white Areas impacted were relationship satisfaction, intimacy, communication, care responsibilities, emotional distancing, frustration, resentment, anger, sadness, fear of future. Three themes related to impacts experienced by CG: (1) altered relationship; (2) care partner challenges; and (3) acceptance and adjustment.	≅ LBD ‐ Few DLB participants; *n* = 3 ≅ Used unspecified diagnostic criteria NOS ✓ Severity moderate‐severe by MoCA ⊗ Disease duration 3‐18 years ⊗ No DLB subgroup analysis ⊗ Small sample size ⊗ Lack of diversity ✓ Patient/Public review panel ✓ Representative quotes ✓ Thematic saturation @10
Donaghy, Barnett, Olsen, et al. (2017)^47^	0	72	To identify symptoms that might aid early diagnosis of Lewy body disease in cases of mild cognitive impairment (MCI) by comparing symptoms of MCI‐LB and DLB vs AD groups and controls.	Clinical Interview + symptom checklist adapted from LBDA Comparisons: MCI‐LB vs. MCI‐AD and DLB vs. AD; healthy controls Recruited from specialty clinic Control: MMSE>26 36 MCI‐LB; 36 DLB Asked: +/‐ each symptom Reports symptom prevalence	Total *N* = 124; 58% DLB/MCI‐LB; 100% Pt; 33% F; Race/ethnicity not reported Symptoms > in DLB and MCI‐LB: fluctuating attention/ concentration, rigidity/stiffness, shuffling, handwriting changes, drooling, frequent falls, posture changes, weak voice, REM sleep behavior disorder, spatial misperception, loss of smell, slowness, visual hallucinations.	✓ 2005 Consensus criteria for DLB, MCI‐LB ✓ 2 Clinicians agreed on diagnosis ✓ CDRS; MMSE, SPECT imaging ✓ Severity mild‐moderate ✓ Subgroup analysis ✓ Systematic evaluation symptoms ⊗ Prevalence w/o bothersomeness ⊗ No participant voice ⊗ No Pt perspective ⊗ Cannot assess diversity
Jackson, Newbronner, Chamberlain et al., (2017)^83^	51	0	To estimate the prevalence of DLB and PDD and care staff's knowledge using a survey of care home managers and interviews with key staff members	Two semi‐structured interview guides, one for managers and nurses; and one for care staff Asked: prevalence of LBD, nature of challenges caring for LBD for staff, extent of staff knowledge and skills, and training Asked: experience of caring for LBD, information available to staff; staff training specific to dementia and LBD	Total *N* = 68 care homes for older people in England& Scotland. Interviews were with 15 nurses, 34 care workers/assistants, 2 activity coordinators caring for LBD. LBD is often under recognized and not diagnosed. Staff felt not well trained or equipped to provide specific care for LBD. Lack of staff awareness about benefits of obtaining a formal diagnosis.	≅ Paid CG of LBD (NOS as PDD or DLB) ⊗ Only 23% were CG of LBD patients ⊗ Diagnostic criteria not used ⊗ Disease severity not reported ⊗ Demographics not clearly stated ⊗ Cannot assess diversity of CG ⊗ Participant voice – few quotes ✓ Example interview questions ✓ Acceptable 48% survey response rate ⊗ No Pt perspective
Watermeyer, Hindle, Roberts, et al. (2016)^56^	26	29	To explore the ability of people with mild to moderate PDD and DLB to set goals for Cognitive Rehab (CR), types of goals, and performance on goals	From: memory clinic, Wales F2F Interviews, 26 CG, 29 Pt Asked: how cognitive symptoms affected ADLs, enjoyment of activities, and social activities. Selected personal goals Eval: goal progress/satisfaction	Total *N* = 54; 100% LBD; 46% CG of LBD; 21% F Pt, 81% F CG; Race not reported Goals were: use technology (email), engage in leisure activities; manage medications; manage self (ADL), orient self to time/place, remember where things are, have social life, manage anxiety.	≅ LBD (NOS PDD or DLB) ✓ DLB consensus criteria (NOS) ✓ Disease severity mild to moderate ⊗ % DLB participants low (4) vs PDD (25) ⊗ % PDD vs. DLB CG not stated ⊗ No DLB subgroup analysis ⊗ Cannot assess diversity ✓ ACE III domains ✓ Participant voice – quotes
Galvin, Duda, Kaufer, et al. (2010)^58^	962	0	To address issues of challenges, burdens, and frustrations facing LBD caregivers in obtaining a diagnoses and care for the patient.	Web‐based survey Unclear if open response Hosted on LBDA website 15 min to complete 83% completion Mean 6.6 yrs from LBD diagnosis Asked: survey not available	Total *N* = 962; 100% LBD; 100% CG; 88% F; Race/ethnicity not reported Presenting symptom were cognitive (48%), motor (39%), or mixed 13%. Multiple visits (>6) and multiple doctors (3.3 ± 1.5) needed to establish LBD diagnosis; 31% took > 2 years. Initial diagnosis was PD, AD, FTD, MDD in 78%.	≅ LBD (NOS PDD or DLB) ⊗ Diagnostic criteria not stated ✓ Severity moderate, severe, after‐death ⊗ Response rate unknown ✓ Single survey per IP address ✓ Presenting symptom frequencies ⊗ Limited demographics ⊗ Cannot assess diversity ⊗ Survey questions not posted ⊗ No participant voice – no quotes
Kashihara, Ohno, Kawada, et al. (2008)^48^	19	19	To explore frequencies of nocturnal vocalization in patients with PAF vs. iPD vs. DLB, defined as three phenotypes of single LB disorder	Clinical interviews w/Pt and spouses iPD vs. PAF, DLB, controls Asked: sleepiness, issues falling asleep, awakenings, excessive daytime vocalizations, dream enactment, vivid dreams, restless legs Comparison by group, age, duration Controls: acute stroke patients	Total *N* = 275 dyads; 7% DLB; 50% CG; LBD group 38% F; Race not reported Sleep problems, vivid dreams, and nocturnal vocalization were more frequent in patients with PAF, IPD and DLB vs. controls. Difficulty falling asleep was similar to stroke controls. Awakenings higher in iPD group. Daytime sleepiness was higher in iPD and DLB.	✓ 2005 DLB consensus criteria ⊗ Small DLB subgroup ✓ DLB Subgroup analysis ✓ Very uneven group sizes ✓ Compared H&Y ✓ Eval w/myocardial scintigraphy ✓ Diversity ‐ Japanese study ✓ Participant voice – no quotes
Bradshaw, Saling, Hopwood, et al. (2004)^49^	13	13	To explore qualitative features of fluctuating cognition (FC) as described by CG of patients with DLB vs. AD, and clinical utility of two recent FC rating scales	Clinical interview +survey: Pt + CG Matched: Pt age, education, severity 13 DLB (69% M) vs. 12 AD (33% M) Asked: Earliest symptoms, onset, course, presenting cognitive complaint as perceived by CG Used one day fluctuation scale and Clinician assessment of fluctuation	Total *N* = 25; 55% Probable DLB; 50% CG; CG gender and race/ethnicity not reported DLB CG cited lapse in awareness or attention, lost ability to engage in meaningful cognitive or physical activity with short lived alterations in cognitive and functional abilities vs. AD CG reporting confusion and more persistent alterations	✓ 1996 DLB consensus criteria (modified) ✓ Mild to moderate DLB ✓ UPDRS, CDRS, MMSE ✓ Dyads and Matched groups ✓ Standardized interview for FC ✓ Assessed frequency and severity ⊗ Small sample size ✓ Subgroup analysis ✓ Patient voice: provides quotes ⊗ Cannot assess diversity
Ballard, McKeith, Harrison, et al. (1997)^50^	‐	42	To evaluate and compare presence and psychopathological features of visual hallucinations between clinically diagnosed DLB and AD.	Clinical interviews with patients Referred: psychiatry (69%) Asked: fluctuations, impaired consciousness, visual hallucinations, psychotic symptoms, falls, mood Postmortem confirmation, 25% of sample at time of report	Total *N* = 73; 58% DLB; 100% Pt; 57% DLB group; Race not reported 93% DLB experienced at least one type of visual hallucination, and 56% had multiple, which was significantly more common than in the AD group.	⊗ Clinical diagnosis DLB or AD ⊗ Disease severity not reported ≅ 1992, 1996 DLB criteria ⊗ Limited demographics ⊗ Cannot assess diversity ⊗ No participant voice

*Notes*: *Specific to DLB or LBD sample size.

Abbreviations: √, Strength of study; ⊗, Limitation of study; ≅ Equivocal; AD, Alzheimer's disease (YO‐AD, Young onset AD); CDRS, Clinical Dementia Rating Scale; CG, Caregiver; DLB, Dementia with Lewy bodies; FAST, Functional Assessment Staging scale; IPD, Idiopathic Parkinson's disease; LBD, Lewy body dementia; F2F, Face to face; FTD, Frontotemporal dementia; MDD, Major depressive disorder; MMSE, Mini Mental State Exam; MoCA, Montreal Cognitive Assessment; MSA, Multiple system atrophy; NOS, Not otherwise specified; PAF, Pure Autonomic failure; PCA, Posterior‐cortical atrophy; PDD, Parkinson's disease dementia; PPA, Primary progressive aphasia; Pt, Patient; RDS, Raredementiasupport.org; RSBD, REM sleep behavior disorder; VD, Vascular dementia.

**TABLE 2B:** Comparison of aims, approaches, and findings from same sample studies.

Author	CG (*N*)	Pt (*N*)	Aim	Approach	Results	Adequacy/Limitations
**Armstrong et al. Study #1**					Total *N* = 30; 100% DLB; 100% CG; 90% F; Race/ethnicity not reported	
Armstrong, Alliance, Corsentino, et al. (2022)^57^	30	0	To explore end‐of‐life (EOL) experiences of informal caregivers of individuals with DLB who died within the prior 5 years	*Three studies in same sample: CG recruited via LBDA website using survey 60 interested out of 400 surveyed; 30 enrolled 30 min phone interviews Conducted post‐mortem Evaluated End of Life (EOL) Semi‐structured Asked: general experiences + specific aspects of EOL Mostly female CG (90%)	Themes: (1) caregivers drive care, (2) DLB symptoms affect EOL for CG, (3) CG role, (4) death + post‐death, and (5) supports for CG. DLB features that impacted CG included: fluctuations, hallucinations, delusions, REM, Autonomic symptoms, Physical limitations, falls.	✓ DLB specific study ⊗ DLB criteria not stated ⊗ Self‐identified CG sample ⊗ Severity not reported ⊗ Demographics not collected ⊗ Cannot assess diversity ⊗ No patient perspective ⊗ Recall bias: 5 yr postmortem ⊗ Brief interview: 30 min ⊗ Sampling bias: Low response ⊗ Recruited from website ✓ Open approach to exploring phenomenon ✓ Caregiver perspective ✓ Representative quotes ✓ Addresses rigor
Armstrong, Alliance, Corsentino, et al. (2020)^59^			To explore barriers to quality end‐of‐life care as perceived by caregivers of individuals with DLB who died within the prior 5 years		Barriers: systems and physician issues, DLB issues, diagnostic inaccuracy, lack of clinician knowledge, prescribing errors, difficulty accessing resources, behavioral changes, and Medicare hospice criteria.	
Armstrong, Alliance, Taylor, et al. (2019)^52^			To explore experiences at end of life as perceived by caregivers of individuals with DLB who died within the prior 5 years		Themes: lack of knowledge about what to expect, end‐of‐life trajectory + symptoms, advance care planning, hospice, right‐to‐die, medications, end of life + death experience, and activities that enhanced end of life, lack of communication, difficulty predicting death.	
**Armstrong et al. Study #2**					Total *N* = 45; 100% DLB; 56% CG; 88% F CG, 10% F Pt; 85% white	
Armstrong, Gamez, Alliance, et al. (2021)^26^	25	20	To investigate (1) aspects of care that are helpful and (2) unmet needs, to guide clinical care	*Two studies in same sample:✓ Clinician judged mild/moderate DLB Excluded MCI and prodromal DLB 30 min phone interviews Mostly dyadic Recruited from LBDA center of excellence Asked: helpful aspects of care, unmet needs, unaddressed symptoms, research priorities.	Themes: Participants valued clinician time, diagnosis, education, symptom management, communication, and caring staff. Needs included education for Pt, CG, non‐specialist clinicians and community providers, scheduling difficulties, CG support, financial concerns, advance care planning, local resources, and effective treatments for symptoms	✓ DLB specific study ⊗ Disease severity unclear ⊗ Self‐identified sample ⊗ Low diversity ⊗ Saturation not reached ⊗ Brief interview: 30 min ⊗ Study closed early due to recruitment challenges ✓ Open approach ✓ Interview guide developed with DLB CG input ✓ Dyadic approach ✓ Representative quotes ✓ Addresses rigor ✓ 2017 DLB consensus criteria ✓ Severity – Mild moderate
Armstrong, Gamez, Alliance, et al. (2020)^51^			To investigate research needs and top priorities for research on DLB symptoms, daily challenges, caregiving/family life, and diagnosis		Themes: Research priorities for Pt and CG were DLB symptoms, therapies to prevent, cure, or delay progression, impact on daily function, quality of life, caregiving, and improving education.	

Abbreviations: √, Strength of study; ⊗, Limitation of study; ≅ Equivocal; ACE III, Addenbrooke's Cognitive Examination III,  AD, Alzheimer's disease (YO‐AD, Young onset AD); CG, Caregiver; DLB, Dementia with Lewy bodies; iPD, Idiopathic Parkinson's disease; LBD, Lewy body dementia; F2F, Face to face; FTD, Frontotemporal dementia; PAF, Pure Autonomic failure; PCA, Posterior‐cortical atrophy; PDD, Parkinson's disease dementia; PPA, Primary progressive aphasia; Pt, Patient; RDS, Raredementiasupport.org; VD, Vascular dementia.

**TABLE 3:** Comparison of key characteristics across studies.

	Groups											LBD/DLB Disease Characteristics							Participant/Informant		Study Focus		Concepts of Interest											Single Symptom Focus					
Reference	DLB	LBD	AD	FTD	MSA	PAF	PD‐MCI	PDD	PPA	PCA	Healthy control	MCI‐LB/ Prodromal	Mild	Moderate	Severe	After death	Neuropsychiatric	Severity not reported	Caregivers	People with DLB/LBD	CG experiences	Patient experiences	CG burden/needs	Patient needs	Intimate relationships	Living with LBD/DLB	End of life experiences	View of Clinical care	Barriers to Care	Goal setting	Research priorities	COVID‐19 lockdown	Burdensome symptoms	Aphasia	Fluctuating cognition	Orthostatic Hypotension	Nocturnal vocalizations	Sleep disturbances	Visual hallucinations
Matterson, Wilson‐Menzfeld^44^	x*													x					x		x	x				x									x				
Yumoto and Suwa^28^	x*													x	x				x	x	x	x				x							x						
Volkmer, Cross^81^		x	x	x						x						x		x	x		x													x					
Harding, Rossi‐Harries^29^	x*		x	x				x	x									x	x	x	x	x										x							
Gallop, Pham^82^	x		x		x	x		x										x	x		x															x			
Killen, Flynn^55^	x*																	x	x	x	x	x	x	x		x													
Stacy, Perazzo^27^		x																x	x		x		x																
Kew, Juengst^41^		x	x										x	x	x				x		x		x																
Brown, Aldridge^42^	x*																	x	x		x							x											
Yumoto and Suwa^45^	x*												x	x	x		x			x		x																	x
van de Beek, van Steenoven^46^		x									x	x								x		x											x						
Cagnin, Di Lorenzo^60^	x*		x	x										x	x		x		x		x		x									x	x		x				
Coindreau, Chesnel^53^	x*																			x		x		x									x						
Larsson, Holmbom‐Larsen^54^	x*												x	x						x		x				x							x						
Vatter, McDonald^43^	x						x	x						x	x				x		x	x			x														
Donaghy, Barnett^47^	x*										x	x	x	x					x	x		x											x						
Jackson, Newbronner^83^	x	x						x										x	x		x		x	x		x		x	x				x		x			x	x
Watermeyer, Hindle^56^	x							x					x	x					x	x		x								x									
Galvin, Duda^58^		x												x	x	x		x	x		x		x																
Kashihara, Ohno^48^	x*					x		x			x							x	x	x		x															x	x	
Bradshaw, Saling^49^	x*		x										x	x					x			x													x				
Ballard, McKeith^50^	x*		x														x		x	x		x																	x
Armstrong, Alliance^57^	x*															x			x		x						x						x						
Armstrong, Alliance^59^	x*															x			x		x						x		x										
Armstrong, Alliance^52^	x*															x			x		x						x												
Armstrong, Gamez^26^	x*												x	x					x	x	x	x	x	x				x											
Armstrong, Gamez^51^	x*												x	x					x	x	x	x									x								

*Notes*: *x = study included a DLB specific analysis either as primary or subgroup analysis.

Abbreviations: DLB, Dementia with Lewy bodies; LBD, Lewy body dementia, which includes PDD and DLB; AD, Alzheimer's dementia; FTD, Frontotemporal dementia; MSA, Multiple system atrophy; PAF, Pure autonomic failure; PDD, Parkinson's disease dementia; PPA, Primary progressive aphasia; PCA, Posterior cortical atrophy; MCI‐LB, Mild cognitive impairment ‐ Lewy body type

**TABLE 4:** GRADE‐CERQual evidence synthesis.

Themes	Coherence of Findings	Adequacy of Data	Relevance
Symptoms & Impacts	Patient: Symptoms: Cognitive, psychiatric, and sleep symptoms were common, as were motor symptoms and common Parkinsonian features. One study reported patients prioritized physical over cognitive complaints. One study noted urinary and GI symptoms Impacts: Patients experienced a range of impacts: difficulty with physical functioning, activity restriction, social isolation, trouble communicating, pain, frustration, and struggling to cope Awareness: Some reported patients were not always aware of symptoms, while others reported good awareness Informal Caregiver: Symptoms: CG also reported cognitive, psychiatric, and sleep symptoms and motor symptoms and common Parkinsonian features Pt + CG impacts; burden of care: In addition to those noted by patients, CG reported relationship and personal impacts (CG burden, emotional distancing, sadness, financial burden, difficulty adjusting, impact on work, CG well‐being). CG burden was assessed most at end‐of‐life	Sixteen studies.^28^, ^29^, ^44^, ^45^, ^46^, ^47^, ^49^, ^50^, ^53^, ^54^, ^57^, ^58^, ^60^, ^81^, ^82^, ^83^ Small sample sizes with few studies that comprehensively evaluated symptoms from the patient or caregiver perspective.^28^, ^47^ Highly heterogeneous: inconsistent populations of interest ranging from LBD, DLB, to MCI‐LB with wide range of disease stages within and across studies. Lack of coherence in samples limits the ability to draw conclusions about groups or phenotypes. Consistent evidence for cognitive, psychiatric, and sleep symptoms from both Pt and CG perspectives^28^, ^29^, ^44^, ^45^, ^46^, ^47^, ^49^, ^50^, ^54^, ^58^, ^60^ Some evidence for motor symptoms and other parkinsonian features from both Pt and CG perspectives from five studies^28^, ^46^, ^47^, ^54^, ^58^ Limited data for bladder symptoms^53^ Evidence for a wide range of impacts, which were rarely systematically reported for patients^28^, ^45^, ^49^, ^54^, ^81^ and somewhat better explored for CGs^29^, ^41^, ^43^, ^44^, ^57^, ^60^, ^81^, ^82^	Findings are highly relevant to understanding the lived experience, types of symptoms experienced in Lewy body dementia, and the impact of symptoms on daily functioning for both the patient and the caregiver.
2. Needs and priorities: Education, support, & research	Patient: Need for greater education about disease symptoms and progression, support for CG, care planning, resources. Research to support better treatment and quality of life. Person specific goals to improve ADL, maintain social life, manage anxiety and symptoms Informal Caregiver: In addition to patient needs and values note above, more information on specific symptoms and symptom management, coping strategies, managing care, meds, appointments, disease trajectories, and EOL Paid Caregiver (care home): Education and training for LBD specific care, greater awareness	Six unique studies, two from the same sample.^26^, ^27^, ^42^, ^51^, ^52^, ^55^, ^56^, ^57^ Four studies with robust qualitative sample sizes >30 participants. Greatest evidence of need for support, especially for caregivers^26^, ^27^, ^42^, ^51^, ^52^, ^55^, ^56^, ^57^ Consistent evidence that more education is needed regarding symptoms + disease trajectory^26^, ^27^, ^52^, ^55^ Limited evidence on research priorities.^51^ Limited evidence for goal setting.^56^	Relevant to identifying needs of patients and caregivers for support, education, and underlying priorities and values with respect to research.
3. Challenges with Clinical Care & Diagnosis	Informal Caregiver: Two studies focusing on difficulties with diagnosis and clinical care, including diagnostic inaccuracy and inefficiency, lack of knowledge, medication problems, specialist access	Two studies with very small sample size.^58^, ^59^ Caregiver perspective only. No patient perspectives reported. Inadequate data to draw robust conclusions.	Relevant to the diagnostic process and clinical care experiences.
4. Effect of Covid lockdown	Patient as perceived by Informal Caregiver: Isolation during COVID‐19 lockdown resulted in worsening of cognitive, behavioral, and psychiatric symptoms for Pt and adversely affected CG. Specific to context of isolation effect.	Two studies^29^, ^60^ looked at the impact of specific contextual situation. Inadequate data to draw robust conclusions.	Context specific (COVID‐19), less relevant to day‐to‐day living.

Abbreviations: AD, Alzheimer's disease; ADL, activities of daily living; CG, caregiver; DLB, dementia with Lewy bodies; EOL, end of life; GI, gastrointestinal; LBD, Lewy body dementia; MCI‐LB, mild cognitive impairment with Lewy bodies; Pt, patient.

## 3 RESULTS

### 3.1 Study characteristics

Our searches produced a total of 704 articles after duplicate removal, from which 678 were excluded (Figure 1). A total of 27 studies met inclusion criteria and underwent full text review and data extraction. Of the 27 studies, 24 were from unique samples. As shown in Tables 2A and 2B, most of the studies (70%) were published in the past 5 years. Most studies were UK or United States based (70%), with the remainder stemming from European countries, Australia, or Japan. The most common data collection approach was brief phone interviews (*N* = 9), followed by surveys with free response options (*N* = 7), face‐to‐face interviews (*N* = 6), and clinical interview/histories (*N* = 5). Sample sizes for most of the studies were small, including as few as 2 DLB participants, and only 11/27 studies (40%) included samples sizes > 30 LBD/DLB participants. The two studies with the largest sample sizes (> 100 participants) were phone or web surveys.

### 3.2 Study populations, perspectives, and phenomena of interest

The studies included data on a total of 2007 individual participants comprising 1668 caregivers (84.1%) and 319 patients/people with LBD (15.9%) as shown in Table 1. Of the caregiver participants, the majority were female (76%), and the patients were mostly male (64%). Study characteristics, methodology, demographics, and thematic findings are presented in Table 2A for unique sample studies and in Table 2B for same sample studies. Only five studies (19%) reported race or ethnicity.^26^, ^27^, ^41^, ^42^, ^43^ All but four studies included caregiver informants (85%), less than half of the studies included patient informants, and one third of studies including both patients and caregiver participants (Table 3). More studies focused on caregiver experiences than patient experiences (67% vs. 59%). Less than half of studies specified diagnostic criteria used for participant inclusion.^26^, ^28^, ^44^, ^45^, ^46^, ^47^, ^48^, ^49^, ^50^, ^51^ Disease characteristics were often incompletely reported. In studies that reported LBD or DLB characteristics, disease severity and stage varied widely, including two studies with prodromal DLB participants (i.e., mild cognitive impairment [MCI‐LB]) and three studies exploring experiences of caregivers of people with DLB who had died within the prior 5 years. In one third of studies, disease severity was not explicitly stated (9/27), and other studies combined data from early through advanced disease in the reports. Only 7/27 (26%) of the studies were comprised of 100% DLB patients or caregivers.^28^, ^44^, ^45^, ^51^, ^52^, ^53^, ^54^ The rest included small DLB subgroups mixed with other dementia populations or focused on LBD in general without reporting the PDD versus DLB distribution of the sample.  Of the studies that included DLB participants (patients and/or caregivers), 18/22 studies (82%) included a DLB‐specific analysis either as primary or subgroup analysis. The rest included small DLB subgroups mixed with other dementia populations or focused on LBD in general without reporting the PDD versus DLB distribution of the sample. Cognitive assessments were heterogeneous (e.g., Mini‐Mental State Examination, Montreal Cognitive Assessment, Clinical Dementia Rating scale), if used in the studies. The most frequent phenomena of interest were burdensome symptoms (*N* = 15 studies) often with a single symptom focus, caregiver burden (*N* = 7), and general lived experiences of LBD/DLB (*N* = 5).

### 3.3 Synthesis of thematic findings and GRADE‐CERQual

A map of thematic categories is shown in Supplements B and C. Main themes and assessment of coherence of findings, adequacy of data and relevance to understanding the lived experience of DLB are presented in Table 4 as ascertained by GRADE‐CERQual approach. Three broad thematic areas were identified: (1) symptoms and impacts for patients and caregivers; (2) caregiver perceptions of challenges with clinical care and diagnostic uncertainty; and (3) the needs and priorities for education, support, and research for patients and caregivers.

As shown in Table 4, more than half of studies explored symptoms and/or functional impacts (16/27; 59%). Most focused on selected symptoms (e.g., behavioral symptoms, visual hallucinations, cognitive fluctuations, hypotension, urinary dysfunction). Only two studies conducted comprehensive symptom assessments.^28^, ^47^ Greatest agreement was found for presence and impact of cognitive‐, psychiatric‐, and sleep‐related symptoms with strong coherence between patient and caregiver groups. Multiple studies noted the presence of parkinsonian motor symptoms (e.g., stiffness, slowness, gait/balance changes). There was limited and inconsistent evidence regarding the accuracy and awareness of patients or caregivers to self‐report symptoms.^28^, ^45^, ^54^ Impacts attributed to LBD/DLB encompassed a wide range of cognitive, physical, and psychosocial functional changes and impairments, which affected both the patient and the caregiver. In general, patient perspectives focused on personal implications of disease (e.g., activities of daily living, social restrictions), whereas caregiver perspectives included the impact on the patient *and* the impact on the caregiver, resulting in a more extensive and diverse range of impacts for caregivers.

A total of eight studies evaluated participants’ needs and priorities, most commonly the need for greater education, caregiver support, and areas of future research in DLB.^26^, ^27^, ^42^, ^51^, ^52^, ^55^, ^56^, ^57^ Educational needs spanned broad areas, including disease‐specific knowledge, what to expect with regard to progression, coping strategies, and planning for the future. The studies focused on needs and priorities had a strong caregiver perspective, and half of these studies included some patient representation (mostly < 4 patients). Only two reports focused on patient perspectives regarding needs and priorities.^26^, ^51^ Two studies explored challenges experienced with clinical care, most often the difficulty with obtaining diagnoses and effective clinical management, particularly when the disease was managed by non‐specialists.^58^, ^59^ Finally, two studies explored the impact of isolation attributed to the COVID‐19 lockdown on the patient and caregiver.^29^, ^60^

## 4 DISCUSSION

This study provides the first qualitative evidence synthesis of perspectives and experiences of people living with DLB and their caregivers. The studies in this review illustrated the complex and heterogenous nature of DLB clinical symptoms, the profound functional and psychosocial impact symptoms have on people with DLB and caregivers, and important gaps in education, care, and research. Coupled with a current lack of effective and safe therapeutics, the symptoms, impacts, and priorities identified here underscore important directions for outcome measure development and clinical trials. Our findings provide additional weight to confront unmet needs in DLB and can contribute to initiatives from government (e.g., AD and Related Dementia Summits, National Alzheimer's Project Act), regulatory, and advocacy agencies to address what matters to patients and families.^19^, ^61^, ^62^, ^63^, ^64^

In this review, patient and family perspectives were broadly aligned with current clinical knowledge of DLB^12^ reflecting the diverse cognitive, non‐motor, and motor manifestations with high degrees of inter‐individual variation.^4^, ^65^ Qualitative data supported cardinal features of DLB diagnostic criteria (i.e., core and supportive), with cognitive decline, fluctuating attention and cognition, visual hallucinations, REM sleep behavior disorder (RBD), and motor parkinsonism most reported. However, other symptoms such as drooling, loss of smell, and weak voice were also noted.^28^, ^47^, ^58^ Multiple reports identified a need for education on symptoms, progression, and management—including strategies to manage behaviors, improve daily functioning, and support caregivers.^26^, ^51^, ^52^, ^57^ Symptoms had marked impacts across multiple dimensions, including physical (bodily discomfort, trouble moving), psychological (frustration, depression, psychological pain), social/communication (isolation, speech issues), and functional effects (confusion, restrictions, inability to engage in meaningful activities). Perspectives of symptoms were similar for patients and caregivers, but caregivers expressed additional impacts, particularly with regard to relationships (communication, intimacy, frustration, anger, sadness) and coping (stress, fear of the future, lack of support). This extends previous findings from quantitative studies of quality of life, caregiver burden and stress, and economic and health‐care costs^6^, ^7^, ^66^, ^67^ by adding a critical human component to understanding “what matters,” which will be essential to driving patient‐centered care. It has been proposed that reframing physician–patient interactions by asking “what matters to you” rather than “what is the matter” could increase awareness of a person's values, preferences, and interests, supporting patient‐centered care, shared decision making, and improved outcomes.^68^

**FIGURE 2:**
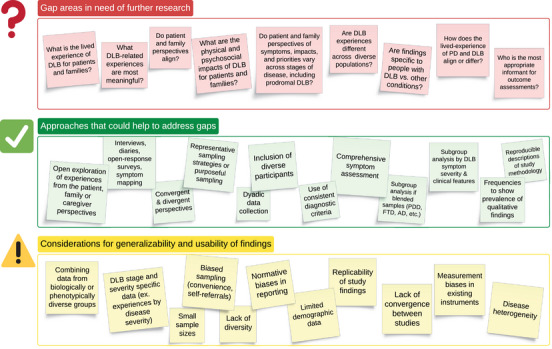
Considerations for future qualitative research in DLB to advance symptom science. AD, Alzheimer's disease; DLB, dementia with Lewy bodies; PD, Parkinson's disease; PDD, Parkinson's disease dementia.

This report draws attention to major gaps in DLB symptom science and a need for rigorous, systematic qualitative research in this area. Figure 2 presents areas in need of further research and key considerations. While most studies were unified by their focus on the lived experience of people with DLB and caregivers, orientation and methodologies varied, with several recurrent weaknesses as discussed below.

First, many of the reviewed studies did not describe criteria for or characteristics of the DLB groups. Different versions of the McKeith diagnostic criteria (1996–2017) were used, with some inconsistently referenced. Similarly, many studies did not report DLB severity, possibly due to lack of DLB‐specific clinical staging system, such as is available for AD (e.g., Global Deterioration Scale, Clinician Dementia Rating scale) and PD (e.g., Hoehn and Yahr scale). Most studies focused on moderate to advanced DLB and several were conducted at end of life.^52^, ^57^, ^59^ In contrast, only two studies included prodromal or MCI‐LB populations^46^, ^47^ and thus, little is known about prodromal or early‐stage DLB perspectives and experiences. Data regarding prodromal DLB experiences is needed to inform clinical trials, outcome measures, and regulatory pathways for disease‐modifying therapeutics that are fit for purpose. With advances in biomarkers and understanding of prodromal DLB (i.e., MCI‐LB, psychiatric onset, delirium onset, RBD, and other presentations) and the publication of prodromal DLB research criteria in 2020, research in this area may expand. Moreover, with proposed biological frameworks and staging systems for synucleinopathies including both PD and DLB, identifying similarities and differences in symptoms and impacts of early PD versus early DLB will be important—especially if these groups are to be combined in biologically based clinical trials.^69^, ^70^, ^71^ While preliminary evidence points to overlap in symptoms between DLB and PDD, few studies have reported experiences with sufficient depth and rigor to draw conclusions with any degree of confidence. Thus, comparison of perspectives of people with clinically manifested DLB versus PD/PDD across functional stages of the proposed biological staging schema is needed, as symptoms and perspectives could vary.

Second, race and ethnicity were rarely reported in the studies, thereby limiting ability to determine sample diversity. To date, little research has been done in diverse ethnoracial groups affected by DLB, but some suggest that prevalence rates, ease of diagnosis, co‐pathologies, treatments, and caregiver burden differ by racial and ethnic groups, globally, and within countries such as the United States.^72^, ^73^, ^74^ Not surprisingly, given the epidemiology of LBD (including DLB), caregiver participants were predominantly females, whereas people with DLB were mainly males. Sex differences have been noted regarding symptomatology (e.g., greater frequency of parkinsonism and RBD in males, hallucinations in females) and diagnosis (e.g., women with DLB tend to be older with greater cognitive impairment at first visit, delayed diagnoses, and co‐existing AD‐related pathology). Reasons for sex differences in DLB are not fully understood but may implicate sex hormones and other factors.^75^, ^76^ Further attention will be needed to evaluate similarities and differences across these and other diverse populations.

Third, most of the qualitative research identified in our systematic review was conducted from the caregiver perspective. Only four studies elicited information directly from people with DLB,^45^, ^46^, ^53^, ^54^ and only one included MCI‐LB participants (37% of the total study sample).^46^ While conducting qualitative studies that include people with dementia can be challenging (e.g., due to cognitive decline, reduced insight, changes in judgment, communication difficulties, concerns about capacity and consent) and people with dementia represent a vulnerable population in research, it is paramount to recognize that people with dementia can provide meaningful and valuable accounts of their experiences. Previous qualitative research has demonstrated people with AD can describe what is important to their quality of life, their coping mechanisms, and their cognitive symptoms.^77^, ^78^ Case study narratives in DLB have also eloquently demonstrated first‐person experiences.^79^, ^80^ While the caregiver input is crucial to understanding the lived experience, inclusion of patient perspectives is important and should be sought when possible, particularly in earlier stages of disease. Research comparing individual and dyadic perspectives would also shed light on shared versus unique experiences and could be useful for clinical outcome assessments (COAs), as there are roles for both direct patient‐reported and informant‐reported measures in clinical trials. At present, there is no consensus on COA development (e.g., determining what is meaningful for patients versus what is meaningful for caregivers) or implementation (e.g., when to use a patient‐reported vs. informant‐reported outcome, how to optimally handle discordancy between patients and caregiver reports, and thresholds for relying on caregiver input in dementia) in LBD trials. Further work will be needed to determine best approaches and most appropriate informants for outcomes assessments in DLB trials.

Strengths of this report include synthesis of qualitative evidence of lived experience perspectives of DLB, descriptions of recent and timely literature, and application of Grade‐CERQual to address the adequacy of data. A comprehensive search strategy was used without time limits, which included LBD and DLB to maximize key characterizations, and conducted independent reviews of the searches and relevant literature. Single person case studies were excluded due to lack of generalizability. We aimed to maximize our review of qualitative literature thereby including interviews, focus groups, and where appropriate, free‐text surveys and chart reviews. Limitations of our review include a small sample size of studies, heterogeneity of the studies, few patient perspectives or dyadic reports, restriction to the English language, and a lack of ethnoracial diversity. Only a small number of studies met inclusion criteria and thematic evidence from this report should be interpreted cautiously and treated as preliminary evidence. Studies were highly heterogeneous with small sample sizes, often with inconsistent populations of interests and disease stages, and predominantly caregiver viewpoints, which limits firm conclusions about patient perspectives, different groups or phenotypes, and various care and research topics. Future efforts should seek to substantiate current findings as well as expand conceptual understanding. This information will be needed to assess the extent of symptomatic alignment across DLB, PDD, and PD and guide selection of key concepts and outcome measures for clinical trials that reflect patient and family priorities for disease modification and symptom management. Thus, we advocate for research to identify the scope of symptoms, including prevalence, bothersomeness, and qualitative characteristics of symptom experiences from early or prodromal to advanced DLB and across demographically diverse and global populations.

## 5 CONCLUSIONS

The findings presented here contribute to understanding the relevance of symptoms and impacts from the patient and caregiver lived experiences in DLB. Bringing the patient and caregiver voice directly into research and care is essential for optimizing clinical trial and therapeutic development, driving patient‐centered care, and thereby improving outcomes across the full disease spectrum.

## AUTHOR CONTRIBUTIONS

*Conception or design of the work*: Jennifer R. Mammen, Jennifer G. Goldman. *Acquisition, analysis, or interpretation of data for the work*: Jennifer G. Goldman, Mirinda Tyo. *Drafting the work and critical review*: Jennifer R. Mammen, Jennifer G. Goldman, Mirinda Tyo. *Final approval of submission*: All authors.

## CONFLICT OF INTEREST STATEMENT

The authors have no conflicts of interest related to the present study. Financial disclosures: Dr. Goldman has received grants/research support from Acadia, Lewy Body Dementia Association, Michael J. Fox Foundation for Parkinson's Research; honoraria from the International Parkinson Disease and Movement Disorders Society, Parkinson's Foundation, Parkinson Study Group; and consulting fees from CervoMed, GE healthcare, KeifeRx, InMuneBio, PaxMedica, SAGE. Dr. Mammen has received grants/research support and/or consulting fees from NIH/NINR, FDA/CDER, Michael J. Fox Foundation for Parkinson's Research and honoraria from Lundbeck. Dr. Tyo has received grants/research support from Michael J. Fox Foundation for Parkinson's Research and FDA/CDER. Xiao is an employee at Michael J. Fox Foundation for Parkinson's Research. Author disclosures are available in the Supporting Information

## CONSENT STATEMENT

Systematic review—Not applicable.

## Supporting information



Supporting Information

Supporting Information

Supporting Information
