# Supplementary material for: Examining the lived experience of dementia with Lewy bodies through qualitative research: A systematic review
Source: Alzheimers Dement. 2025 May 15;21(5):e70217. doi: 10.1002/alz.70217 (PMC12079417; doi:10.1002/alz.70217)
Supplement: Supplementary file 2 — Supporting Information [file ALZ-21-e70217-s003.pdf]

Final audit trail for DLB Qualitative landscape assessment

Included in Review (Qualitative with DLB/LBD focus)

[1] [2] [3] [4] [5] [6] [7] [8] [9] [10] [11] [12] [13] [14] [15] [16] [17] [18] [19] [20] [21] [22] [23] [24] [25] [26] [27]

Primary Search – Pubmed

- Included = [3-16, 18-27]
- Review articles = 137. [28-164]
- Commentary/Editorial = 14. [165-178]
- State of science report = 9. [179-187]
- Guidelines = 4. [188-191]
- Case studies = 30. [192-221]
- Quantitative w/ some DLB focus = 96 (Possible Tier 2/3). [222-317]
- Epidemiology = 17. [318-334]
- Biomarker & genetics = 90. [335-424]
- Diagnostic Imaging = 28. [425-452]
- Efficacy trials = 15. [453-467]
- Instrument development = 21. [468-488]
- Animal models = 10. [489-498]
- Protocol/methods papers = 8. [499-506]
- No DLB focus/participants = 79. [507-585]
- Off topic = 82. [586-667]

Secondary Searches

Web of Science

- Duplicates =173. [245, 259, 261, 272, 273, 286, 311, 315, 668-832]
- Quantitative = 17. [744, 833-848]
- Included = 2. [17, 849]

Psych Info

- Duplicates = 98. [259, 311, 315, 669, 672, 679, 681, 682, 696, 708, 710, 711, 714, 724, 743, 745, 747, 752, 760, 765, 779, 780, 782, 790, 796, 797, 800, 801, 805, 807, 811, 814-816, 840, 846, 850-911]
- Quantitative = 19. [912-929]
- Dissertation = 1. [930]
- Included = 0

CINHAL

- Duplicates = 66. [259, 261, 315, 669, 672, 696, 708, 716, 723, 736, 752, 763, 765, 768, 779, 782, 796, 797, 800, 804, 807, 808, 814-816, 822, 874, 887, 889, 891, 897, 900, 909, 931-963]
- Quantitative = 2. [946, 964]
- Included = 0

Reference list screen of related articles

The following articles were screened for reference lists: [1, 2, 40-42, 74, 88, 98, 133, 156, 157, 186, 209, 499, 502, 505, 930, 965]

- New studies identified from screen included = 1. [965]

References

[1] Matterson E, Wilson-Menzfeld G, Olsen K, Taylor JP, Elder GJ (2024) Understanding the nature and impact of cognitive fluctuations and sleep disturbances in dementia with Lewy bodies: A qualitative caregiver study. *SAGE Open Med* **12**, 20503121241271827. doi:10.1177/20503121241271827

[2] Yumoto A, Suwa S (2024) Daily Life Experiences of People with Dementia with Lewy Bodies: A Qualitative Study. *Sage Open* **14**, 21582440241287659. doi:10.1177/21582440241287659

[3] Volkmer A, Cross L, Highton L, Jackson C, Smith C, Brotherhood E, Harding EV, Mummery C, Rohrer J, Weil R, Yong K, Crutch S, Hardy CJD (2024) 'Communication is difficult': Speech, language and communication needs of people with young onset or rarer forms of non-language led dementia. *Int J Lang Commun Disord* **59**, 1553-1577. doi:10.1111/1460-6984.13018

[4] Harding E, Rossi-Harries S, Gerritzen EV, Zimmerman N, Hoare Z, Proctor D, Brotherhood E, Crutch S, Suárez-González A (2023) "I felt like I had been put on the shelf and forgotten about" - lasting lessons about the impact of COVID-19 on people affected by rarer dementias. *BMC Geriatr* **23**, 392. doi:10.1186/s12877-023-03992-1

[5] Gallop K, Pham N, Maclaine G, Saunders E, Black B, Acaster S (2023) Exploring the impact of caring for an individual with neurogenic orthostatic hypotension: a qualitative study. *Neurodegener Dis Manag* **13**, 191-201. doi:10.2217/nmt-2022-0016

[6] Killen A, Flynn D, O'Brien N, Taylor JP (2022) The feasibility and acceptability of a psychosocial intervention to support people with dementia with Lewy bodies and family care partners. *Dementia (London)* **21**, 77-93. doi:10.1177/14713012211028501

[7] Stacy KE, Perazzo J, Shatz R, Bakas T (2022) Needs and Concerns of Lewy Body Disease Family Caregivers: A Qualitative Study. *West J Nurs Res* **44**, 227-238. doi:10.1177/01939459211050957

[8] Kew CL, Juengst SB, Kelley B, Osborne CL (2022) Linking Problems Reported by Care Partners of Individuals With Alzheimer's Disease and Lewy Body Dementia to the International Classification of Functioning Disability and Health. *Gerontol Geriatr Med* **8**, 23337214221086810. doi:10.1177/23337214221086810

[9] Brown LJE, Aldridge Z, Pepper A, Leroi I, Dening KH (2022) 'It's just incredible the difference it has made': family carers' experiences of a specialist Lewy body dementia Admiral Nurse service. *Age Ageing* **51**. doi:10.1093/ageing/afac207

[10] Yumoto A, Suwa S (2021) Difficulties and associated coping methods regarding visual hallucinations caused by dementia with Lewy bodies. *Dementia (London)* **20**, 291-307. doi:10.1177/1471301219879541

[11] van de Beek M, van Steenoven I, van der Zande JJ, Barkhof F, Teunissen CE, van der Flier WM, Lemstra AW (2020) Prodromal Dementia With Lewy Bodies: Clinical Characterization and Predictors of Progression. *Mov Disord* **35**, 859-867. doi:10.1002/mds.27997

[12] Cagnin A, Di Lorenzo R, Marra C, Bonanni L, Cupidi C, Laganà V, Rubino E, Vacca A, Provero P, Isella V, Vanacore N, Agosta F, Appollonio I, Caffarra P, Pettenuzzo I, Sambati R, Quaranta D, Guglielmi V, Logroscino G, Filippi M, Tedeschi G, Ferrarese C, Rainero I, Bruni AC (2020) Behavioral and Psychological Effects of Coronavirus Disease-19 Quarantine in Patients With Dementia. *Front Psychiatry* **11**, 578015. doi:10.3389/fpsyt.2020.578015

[13] Coindreau V, Chesnel C, Babany F, Declemy A, Savard E, Charlanes A, Lebreton F, Amarenco G (2020) [Urinary tract symptoms in Lewy body dementia: About 19 cases]. *Prog Urol* **30**, 267-272. doi:10.1016/j.purol.2020.02.007

[14] Larsson V, Holmbom-Larsen A, Torisson G, Strandberg EL, Londos E (2019) Living with dementia with Lewy bodies: an interpretative phenomenological analysis. *BMJ Open* **9**, e024983. doi:10.1136/bmjopen-2018-024983

[15] Vatter S, McDonald KR, Stanmore E, Clare L, McCormick SA, Leroi I (2018) A qualitative study of female caregiving spouses' experiences of intimate relationships as cognition declines in Parkinson's disease. *Age Ageing* **47**, 604-610. doi:10.1093/ageing/afy049

[16] Donaghy PC, Barnett N, Olsen K, Taylor JP, McKeith IG, O'Brien JT, Thomas AJ (2017) Symptoms associated with Lewy body disease in mild cognitive impairment. *Int J Geriatr Psychiatry* **32**, 1163-1171. doi:10.1002/gps.4742

[17] Jackson GA, Newbronner L, Chamberlain R, Borthwick R, Yardley C, Boyle K (2017) Caring for people with dementia with Lewy bodies and Parkinson's dementia in UK care homes - A mixed methods study. *European Geriatric Medicine* **8**, 146-152. doi:10.1016/j.eurger.2017.01.003

[18] Watermeyer TJ, Hindle JV, Roberts J, Lawrence CL, Martyr A, Lloyd-Williams H, Brand A, Gutting P, Hoare Z, Edwards RT, Clare L (2016) Goal Setting for Cognitive Rehabilitation in Mild to Moderate Parkinson's Disease Dementia and Dementia with Lewy Bodies. *Parkinsons Dis* **2016**, 8285041. doi:10.1155/2016/8285041

[19] Galvin JE, Duda JE, Kaufer DI, Lippa CF, Taylor A, Zarit SH (2010) Lewy body dementia: the caregiver experience of clinical care. *Parkinsonism Relat Disord* **16**, 388-392. doi:10.1016/j.parkreldis.2010.03.007

[20] Kashihara K, Ohno M, Kawada S, Imamura T (2008) Frequent nocturnal vocalization in pure autonomic failure. *J Int Med Res* **36**, 489-495. doi:10.1177/147323000803600313

[21] Bradshaw J, Saling M, Hopwood M, Anderson V, Brodtmann A (2004) Fluctuating cognition in dementia with Lewy bodies and Alzheimer's disease is qualitatively distinct. *J Neurol Neurosurg Psychiatry* **75**, 382-387. doi:10.1136/jnnp.2002.002576

[22] Ballard C, McKeith I, Harrison R, O'Brien J, Thompson P, Lowery K, Perry R, Ince P (1997) A detailed phenomenological comparison of complex visual hallucinations in dementia with Lewy bodies and Alzheimer's disease. *Int Psychogeriatr* **9**, 381-388. doi:10.1017/s1041610297004523

[23] Armstrong MJ, Alliance S, Corsentino P, Lunde A, Taylor A (2022) Informal caregiver experiences at the end-of-life of individuals living with dementia with Lewy bodies: An interview study. *Dementia (London)* **21**, 287-303. doi:10.1177/14713012211038428

[24] Armstrong MJ, Alliance S, Corsentino P, Maixner SM, Paulson HL, Taylor A (2020) Caregiver-Reported Barriers to Quality End-of-Life Care in Dementia With Lewy Bodies: A Qualitative Analysis. *Am J Hosp Palliat Care* **37**, 728-737. doi:10.1177/1049909119897241
[25] Armstrong MJ, Alliance S, Taylor A, Corsentino P, Galvin JE (2019) End-of-life experiences in dementia with Lewy bodies: Qualitative interviews with former caregivers. *PLoS One* **14**, e0217039. doi:10.1371/journal.pone.0217039
[26] Armstrong MJ, Gamez N, Alliance S, Majid T, Taylor AS, Kurasz AM, Patel B, Smith GE (2021) Clinical Care and Unmet Needs of Individuals With Dementia With Lewy Bodies and Caregivers: An Interview Study. *Alzheimer Dis Assoc Disord* **35**, 327-334. doi:10.1097/wad.0000000000000459
[27] Armstrong MJ, Gamez N, Alliance S, Majid T, Taylor A, Kurasz AM, Patel B, Smith G (2020) Research priorities of caregivers and individuals with dementia with Lewy bodies: An interview study. *PLoS One* **15**, e0239279. doi:10.1371/journal.pone.0239279
[28] (2009) [Sleep behavior disorder (RBD) in synucleionopaties]. *Glas Srp Akad Nauka Med*, 7-15.
[29] Aarsland D (2020) Epidemiology and Pathophysiology of Dementia-Related Psychosis. *J Clin Psychiatry* **81**. doi:10.4088/jcp.Ad19038br1c
[30] Agnati LF, Baldelli E, Andreoli N, Woods AS, Vellani V, Marcellino D, Guidolin D, Fuxe K (2008) On the key role played by altered protein conformation in Parkinson's disease. *J Neural Transm (Vienna)* **115**, 1285-1299. doi:10.1007/s00702-008-0072-1
[31] Aiba I (2012) [Corticobasal syndrome: recent advances and future directions]. *Brain Nerve* **64**, 462-473.
[32] Arnulf I (2012) REM sleep behavior disorder: motor manifestations and pathophysiology. *Mov Disord* **27**, 677-689. doi:10.1002/mds.24957
[33] Arsland D (2002) [Dementia with Lewy bodies]. *Tidsskr Nor Laegeforen* **122**, 525-529.
[34] Attreed A, Morand LR, Pond DC, Sturmberg JP (2024) The Clinical Role of Heart Rate Variability Assessment in Cognitively Impaired Patients and Its Applicability in Community Care Settings: A Systematic Review of the Literature. *Cureus* **16**, e61703. doi:10.7759/cureus.61703
[35] Banerjee S, Argáez C (2021) CADTH Health Technology Review In *Patient Navigation Programs for People With Dementia* Canadian Agency for Drugs and Technologies in Health
Copyright © 2021 Canadian Agency for Drugs and Technologies in Health., Ottawa (ON).
[36] Baquero M, Martín N (2015) Depressive symptoms in neurodegenerative diseases. *World J Clin Cases* **3**, 682-693. doi:10.12998/wjcc.v3.i8.682
[37] Barton JJS (2021) Motion perception and its disorders. *Handb Clin Neurol* **178**, 257-275. doi:10.1016/b978-0-12-821377-3.00013-1
[38] Belbeze J, Gallarda T (2020) Very-late-onset psychotic symptoms: psychosis or dementia? A phenomenological approach. A systematic review. *Geriatr Psychol Neuropsychiatr Vieil* **18**, 77-86. doi:10.1684/pnv.2020.0868

[39] Bellomo G, De Luca CMG, Paoletti FP, Gaetani L, Moda F, Parnetti L (2022) α-Synuclein Seed Amplification Assays for Diagnosing Synucleinopathies: The Way Forward. *Neurology* **99**, 195-205. doi:10.1212/wnl.0000000000200878
[40] Bentley A, Morgan T, Salifu Y, Walshe C (2021) Exploring the experiences of living with Lewy body dementia: An integrative review. *J Adv Nurs* **77**, 4632-4645. doi:10.1111/jan.14932
[41] Bhidayasiri R, Jitkritsadakul O, Colosimo C (2014) Nocturnal manifestations of atypical parkinsonian disorders. *J Parkinsons Dis* **4**, 223-236. doi:10.3233/jpd-130280
[42] Binnekade TT, Van Kooten J, Lobbezoo F, Rhebergen D, Van der Wouden JC, Smalbrugge M, Scherder EJA (2017) Pain Experience in Dementia Subtypes: A Systematic Review. *Curr Alzheimer Res* **14**, 471-485. doi:10.2174/1567205013666160602234109
[43] Blanc F, Verny M (2017) Prodromal stage of disease (dementia) with Lewy bodies, how to diagnose in practice? *Geriatr Psychol Neuropsychiatr Vieil* **15**, 196-204. doi:10.1684/pnv.2017.0675
[44] Boeve BF, Silber MH, Ferman TJ (2004) REM sleep behavior disorder in Parkinson's disease and dementia with Lewy bodies. *J Geriatr Psychiatry Neurol* **17**, 146-157. doi:10.1177/0891988704267465
[45] Bombois S, Derambure P, Pasquier F, Monaca C (2010) Sleep disorders in aging and dementia. *J Nutr Health Aging* **14**, 212-217. doi:10.1007/s12603-010-0052-7
[46] Bradfield NI (2023) Mild Cognitive Impairment: Diagnosis and Subtypes. *Clin EEG Neurosci* **54**, 4-11. doi:10.1177/15500594211042708
[47] Braithwaite SP, Stock JB, Mouradian MM (2012) α-Synuclein phosphorylation as a therapeutic target in Parkinson's disease. *Rev Neurosci* **23**, 191-198. doi:10.1515/revneuro-2011-0067
[48] Bramich S, King A, Kuruvilla M, Naismith SL, Noyce A, Alty J (2022) Isolated REM sleep behaviour disorder: current diagnostic procedures and emerging new technologies. *J Neurol* **269**, 4684-4695. doi:10.1007/s00415-022-11213-9
[49] Brás IC, Outeiro TF (2021) Alpha-Synuclein: Mechanisms of Release and Pathology Progression in Synucleinopathies. *Cells* **10**. doi:10.3390/cells10020375
[50] Burghaus L, Eggers C, Timmermann L, Fink GR, Diederich NJ (2012) Hallucinations in neurodegenerative diseases. *CNS Neurosci Ther* **18**, 149-159. doi:10.1111/j.1755-5949.2011.00247.x
[51] Burn DJ (2005) Update on dementia with Lewy bodies. *Curr Neurol Neurosci Rep* **5**, 339-344. doi:10.1007/s11910-005-0057-5
[52] Candela S, Giubilei F, Orzi F (2013) Heterogeneous pathologies associated with dementia in Parkinsonism share a prion-like spreading mechanism. *Arch Ital Biol* **151**, 169-178.
[53] Caviness JN (2007) Parkinsonism & related disorders. Myoclonus. *Parkinsonism Relat Disord* **13 Suppl 3**, S375-384. doi:10.1016/s1353-8020(08)70033-6
[54] Chen HH, Hu CJ (2006) Genetic characteristics of dementia in Taiwan. *Acta Neurol Taiwan* **15**, 161-169.

[55] Chin KS, Teodorczuk A, Watson R (2019) Dementia with Lewy bodies: Challenges in the diagnosis and management. *Aust N Z J Psychiatry* **53**, 291-303. doi:10.1177/0004867419835029
[56] Chiu MJ (2007) [Memory and memory disorders]. *Acta Neurol Taiwan* **16**, 242-250.
[57] Chiu SY, Wyman-Chick KA, Ferman TJ, Bayram E, Holden SK, Choudhury P, Armstrong MJ (2023) Sex differences in dementia with Lewy bodies: Focused review of available evidence and future directions. *Parkinsonism Relat Disord* **107**, 105285. doi:10.1016/j.parkreldis.2023.105285
[58] Cipriani G, Danti S, Vedovello M, Nuti A, Lucetti C (2014) Understanding delusion in dementia: a review. *Geriatr Gerontol Int* **14**, 32-39. doi:10.1111/ggi.12105
[59] Clayton DF, George JM (1999) Synucleins in synaptic plasticity and neurodegenerative disorders. *J Neurosci Res* **58**, 120-129.
[60] Cooper CA, Chahine LM (2016) Biomarkers in Prodromal Parkinson Disease: a Qualitative Review. *J Int Neuropsychol Soc* **22**, 956-967. doi:10.1017/s1355617716000503
[61] Cressot C, Vrillon A, Lilamand M, Francisque H, Méauzoone A, Hourregue C, Dumurgier J, Marlinge E, Paquet C, Cognat E (2024) Psychosis in Neurodegenerative Dementias: A Systematic Comparative Review. *J Alzheimers Dis* **99**, 85-99. doi:10.3233/jad-231363
[62] Cummings J (2021) The Role of Neuropsychiatric Symptoms in Research Diagnostic Criteria for Neurodegenerative Diseases. *Am J Geriatr Psychiatry* **29**, 375-383. doi:10.1016/j.jagp.2020.07.011
[63] Das T, Hwang JJ, Poston KL (2019) Episodic recognition memory and the hippocampus in Parkinson's disease: A review. *Cortex* **113**, 191-209. doi:10.1016/j.cortex.2018.11.021
[64] Defebvre L (2006) Myoclonus and extrapyramidal diseases. *Neurophysiol Clin* **36**, 319-325. doi:10.1016/j.neucli.2006.11.003
[65] Defebvre L (2007) [Extrapyramidal disorders: interest of myoclonus analysis]. *Rev Neurol (Paris)* **163**, 1115-1121. doi:10.1016/s0035-3787(07)74188-7
[66] Desmarais P, Massoud F, Filion J, Nguyen QD, Bajsarowicz P (2016) Quetiapine for Psychosis in Parkinson Disease and Neurodegenerative Parkinsonian Disorders: A Systematic Review. *J Geriatr Psychiatry Neurol* **29**, 227-236. doi:10.1177/0891988716640378
[67] Dethy S, Hambye AS (2008) [1231-FP-CIT (DaTSCAN) scintigraphy in the differential diagnosis of movement disorders]. *Rev Med Brux* **29**, 238-247.
[68] Devenyi RA, Hamedani AG (2024) Visual dysfunction in dementia with Lewy bodies. *Curr Neurol Neurosci Rep* **24**, 273-284. doi:10.1007/s11910-024-01349-8
[69] Dietrichs E, Kvikstad V (2008) [Essential tremor]. *Tidsskr Nor Laegeforen* **128**, 2210-2213.
[70] Donaghy PC, McKeith IG (2014) The clinical characteristics of dementia with Lewy bodies and a consideration of prodromal diagnosis. *Alzheimers Res Ther* **6**, 46. doi:10.1186/alzrt274

[71] Dudchenko NG, Vasenina EE (2020) [Fluctuation of cognitive functions in dementia with Lewy bodies]. *Zh Nevrol Psikhiatr Im S S Korsakova* **120**, 89-95. doi:10.17116/jnevro202012010289
[72] Engedal K (2002) [Diagnosis and treatment of dementia]. *Tidsskr Nor Laegeforen* **122**, 520-524.
[73] Esmaeeli S, Murphy K, Swords GM, Ibrahim BA, Brown JW, Llano DA (2019) Visual hallucinations, thalamocortical physiology and Lewy body disease: A review. *Neurosci Biobehav Rev* **103**, 337-351. doi:10.1016/j.neubiorev.2019.06.006
[74] Eversfield CL, Orton LD (2019) Auditory and visual hallucination prevalence in Parkinson's disease and dementia with Lewy bodies: a systematic review and meta-analysis. *Psychol Med* **49**, 2342-2353. doi:10.1017/s0033291718003161
[75] Farlow M (2002) A clinical overview of cholinesterase inhibitors in Alzheimer's disease. *Int Psychogeriatr* **14 Suppl 1**, 93-126. doi:10.1017/s1041610203008688
[76] Fernandez HH, Wu CK, Ott BR (2003) Pharmacotherapy of dementia with Lewy bodies. *Expert Opin Pharmacother* **4**, 2027-2037. doi:10.1517/14656566.4.11.2027
[77] Ferrer I, Martinez A, Blanco R, Dalfó E, Carmona M (2011) Neuropathology of sporadic Parkinson disease before the appearance of parkinsonism: preclinical Parkinson disease. *J Neural Transm (Vienna)* **118**, 821-839. doi:10.1007/s00702-010-0482-8
[78] Foley P, Riederer P (1999) Pathogenesis and preclinical course of Parkinson's disease. *J Neural Transm Suppl* **56**, 31-74. doi:10.1007/978-3-7091-6360-3_2
[79] Gallant NL, Russill CL, Taylor NC, Nakonechny S, Kohlert A, Ewing K (2024) Time perception among people living with and without dementia: A scoping review. *Dementia (London)*, 14713012241257299. doi:10.1177/14713012241257299
[80] Gerlach M, Riederer P (1996) Animal models of Parkinson's disease: an empirical comparison with the phenomenology of the disease in man. *J Neural Transm (Vienna)* **103**, 987-1041. doi:10.1007/bf01291788
[81] Goldstein DS (2020) "Sick-but-not-dead": multiple paths to catecholamine deficiency in Lewy body diseases. *Stress* **23**, 633-637. doi:10.1080/10253890.2020.1765158
[82] Goldstein DS (2020) The "Sick-but-not-Dead" Phenomenon Applied to Catecholamine Deficiency in Neurodegenerative Diseases. *Semin Neurol* **40**, 502-514. doi:10.1055/s-0040-1713874
[83] Grossberg GT (2002) The ABC of Alzheimer's disease: behavioral symptoms and their treatment. *Int Psychogeriatr* **14 Suppl 1**, 27-49. doi:10.1017/s1041610203008652
[84] Hansen N (2021) Current Nosology of Neural Autoantibody-Associated Dementia. *Front Aging Neurosci* **13**, 711195. doi:10.3389/fnagi.2021.711195
[85] Haw C, Harwood D, Hawton K (2009) Dementia and suicidal behavior: a review of the literature. *Int Psychogeriatr* **21**, 440-453. doi:10.1017/s1041610209009065
[86] Hinkle JT, Pontone GM (2020) Lewy Body Degenerations as Neuropsychiatric Disorders. *Psychiatr Clin North Am* **43**, 361-381. doi:10.1016/j.psc.2020.02.003

[87] Ince PG, Perry EK, Morris CM (1998) Dementia with Lewy bodies. A distinct non-Alzheimer dementia syndrome? *Brain Pathol* **8**, 299-324. doi:10.1111/j.1750-3639.1998.tb00156.x
[88] Inskip M, Mavros Y, Sachdev PS, Fiatarone Singh MA (2016) Exercise for Individuals with Lewy Body Dementia: A Systematic Review. *PLoS One* **11**, e0156520. doi:10.1371/journal.pone.0156520
[89] Ishikawa A, Takahashi H (1998) Clinical and neuropathological aspects of autosomal recessive juvenile parkinsonism. *J Neurol* **245**, P4-9. doi:10.1007/pl00007745
[90] Jellinger KA (2012) Cerebral correlates of psychotic syndromes in neurodegenerative diseases. *J Cell Mol Med* **16**, 995-1012. doi:10.1111/j.1582-4934.2011.01311.x
[91] Johnson JCS, McWhirter L, Hardy CJD, Crutch SJ, Marshall CR, Mummery CJ, Rohrer JD, Rossor MN, Schott JM, Weil RS, Fox NC, Warren JD (2021) Suspecting dementia: canaries, chameleons and zebras. *Pract Neurol*. doi:10.1136/practneurol-2021-003019
[92] Jreige M, Kurian GK, Perriraz J, Potheegadoo J, Bernasconi F, Stampacchia S, Blanke O, Alessandra G, Lejay N, Chiabotti PS, Rouaud O, Nicod Lalonde M, Schaefer N, Treglia G, Allali G, Prior JO (2023) The diagnostic performance of functional dopaminergic scintigraphic imaging in the diagnosis of dementia with Lewy bodies: an updated systematic review. *Eur J Nucl Med Mol Imaging* **50**, 1988-2035. doi:10.1007/s00259-023-06154-y
[93] Jurek L, Herrmann M, Bonze M, Brunet S, Padovan C, Dorey JM (2018) Behavioral and psychological symptoms in Lewy body disease: a review. *Geriatr Psychol Neuropsychiatr Vieil* **16**, 87-95. doi:10.1684/pnv.2018.0723
[94] Kalra S, Bergeron C, Lang AE (1996) Lewy body disease and dementia. A review. *Arch Intern Med* **156**, 487-493.
[95] Kaufer DI (2004) Pharmacologic treatment expectations in the management of dementia with Lewy bodies. *Dement Geriatr Cogn Disord* **17 Suppl 1**, 32-39. doi:10.1159/000074680
[96] Kaufmann H, Biaggioni I (2003) Autonomic failure in neurodegenerative disorders. *Semin Neurol* **23**, 351-363. doi:10.1055/s-2004-817719
[97] Killinger BA, Kordower JH (2019) Spreading of alpha-synuclein - relevant or epiphenomenon? *J Neurochem* **150**, 605-611. doi:10.1111/jnc.14779
[98] Kindell J, Keady J, Sage K, Wilkinson R (2017) Everyday conversation in dementia: a review of the literature to inform research and practice. *Int J Lang Commun Disord* **52**, 392-406. doi:10.1111/1460-6984.12298
[99] Kinoshita T, Hanabusa H (2010) Issues facing home-based medical support services. *Psychogeriatrics* **10**, 90-94. doi:10.1111/j.1479-8301.2010.00315.x
[100] Kirk A (2007) Target symptoms and outcome measures: cognition. *Can J Neurol Sci* **34 Suppl 1**, S42-46. doi:10.1017/s0317167100005552
[101] Korczyn AD (2013) Mild cognitive impairment in Parkinson's disease. *J Neural Transm (Vienna)* **120**, 517-521. doi:10.1007/s00702-013-1006-0

[102] Lai YY, Siegel JM (2003) Physiological and anatomical link between Parkinson-like disease and REM sleep behavior disorder. *Mol Neurobiol* **27**, 137-152. doi:10.1385/mn:27:2:137

[103] Law ZK, Todd C, Mehraram R, Schumacher J, Baker MR, LeBeau FEN, Yarnall A, Onofrj M, Bonanni L, Thomas A, Taylor JP (2020) The Role of EEG in the Diagnosis, Prognosis and Clinical Correlations of Dementia with Lewy Bodies-A Systematic Review. *Diagnostics (Basel)* **10**. doi:10.3390/diagnostics10090616

[104] Lawn T, Ffytche D (2021) Cerebellar involvement in hallucinations may transcend clinical conditions and perceptual modalities. *Cortex* **143**, 290-294. doi:10.1016/j.cortex.2021.07.010

[105] Lee DR, Taylor JP, Thomas AJ (2012) Assessment of cognitive fluctuation in dementia: a systematic review of the literature. *Int J Geriatr Psychiatry* **27**, 989-998. doi:10.1002/gps.2823

[106] Leu-Semenescu S, Arnulf I (2010) [Disruptive nocturnal behavior in elderly subjects: could it be a parasomnia?]. *Psychol Neuropsychiatr Vieil* **8**, 97-109. doi:10.1684/pnv.2010.0210

[107] Levy JA, Chelune GJ (2007) Cognitive-behavioral profiles of neurodegenerative dementias: beyond Alzheimer's disease. *J Geriatr Psychiatry Neurol* **20**, 227-238. doi:10.1177/0891988707308806

[108] Lewitt PA (2012) Norepinephrine: the next therapeutics frontier for Parkinson's disease. *Transl Neurodegener* **1**, 4. doi:10.1186/2047-9158-1-4

[109] LoBue C, Munro C, Schaffert J, Didehbani N, Hart J, Batjer H, Cullum CM (2019) Traumatic Brain Injury and Risk of Long-Term Brain Changes, Accumulation of Pathological Markers, and Developing Dementia: A Review. *J Alzheimers Dis* **70**, 629-654. doi:10.3233/jad-190028

[110] Luk KC, Lee VM (2014) Modeling Lewy pathology propagation in Parkinson's disease. *Parkinsonism Relat Disord* **20 Suppl 1**, S85-87. doi:10.1016/s1353-8020(13)70022-1

[111] Maclin JMA, Wang T, Xiao S (2019) Biomarkers for the diagnosis of Alzheimer's disease, dementia Lewy body, frontotemporal dementia and vascular dementia. *Gen Psychiatr* **32**, e100054. doi:10.1136/gpsych-2019-100054

[112] Manford M, Andermann F (1998) Complex visual hallucinations. Clinical and neurobiological insights. *Brain* **121 (Pt 10)**, 1819-1840. doi:10.1093/brain/121.10.1819

[113] Marsili L, Bologna M, Kojovic M, Berardelli A, Espay AJ, Colosimo C (2019) Dystonia in atypical parkinsonian disorders. *Parkinsonism Relat Disord* **66**, 25-33. doi:10.1016/j.parkreldis.2019.07.030

[114] Matar E, Shine JM, Halliday GM, Lewis SJG (2020) Cognitive fluctuations in Lewy body dementia: towards a pathophysiological framework. *Brain* **143**, 31-46. doi:10.1093/brain/awz311

[115] Miquel-Rio L, Sarriés-Serrano U, Pavia-Collado R, Meana JJ, Bortolozzi A (2023) The Role of α-Synuclein in the Regulation of Serotonin System: Physiological and Pathological Features. *Biomedicines* **11**. doi:10.3390/biomedicines11020541

[116] Mitra K, Gangopadhaya PK, Das SK (2003) Parkinsonism plus syndrome--a review. *Neurol India* **51**, 183-188.

[117] Moreira PI, Siedlak SL, Aliev G, Zhu X, Cash AD, Smith MA, Perry G (2005) Oxidative stress mechanisms and potential therapeutics in Alzheimer disease. *J Neural Transm (Vienna)* **112**, 921-932. doi:10.1007/s00702-004-0242-8
[118] Mori E (2000) [Dementia with Lewy bodies]. *Nihon Ronen Igakkai Zasshi* **37**, 772-776. doi:10.3143/geriatrics.37.772
[119] Morris SK, Olichney JM, Corey-Bloom J (1998) Psychosis in Dementia With Lewy Bodies. *Semin Clin Neuropsychiatry* **3**, 51-60.
[120] Nelson PT, Lee EB, Cykowski MD, Alafuzoff I, Arfanakis K, Attems J, Brayne C, Corrada MM, Dugger BN, Flanagan ME, Ghetti B, Grinberg LT, Grossman M, Grothe MJ, Halliday GM, Hasegawa M, Hokkanen SRK, Hunter S, Jellinger K, Kawas CH, Keene CD, Kouri N, Kovacs GG, Leverenz JB, Latimer CS, Mackenzie IR, Mao Q, McAleese KE, Merrick R, Montine TJ, Murray ME, Myllykangas L, Nag S, Neltner JH, Newell KL, Rissman RA, Saito Y, Sajjadi SA, Schwetye KE, Teich AF, Thal DR, Tomé SO, Troncoso JC, Wang SJ, White CL, 3rd, Wisniewski T, Yang HS, Schneider JA, Dickson DW, Neumann M (2023) LATE-NC staging in routine neuropathologic diagnosis: an update. *Acta Neuropathol* **145**, 159-173. doi:10.1007/s00401-022-02524-2
[121] Neupane S, De Cecco E, Aguzzi A (2023) The Hidden Cell-to-Cell Trail of α-Synuclein Aggregates. *J Mol Biol* **435**, 167930. doi:10.1016/j.jmb.2022.167930
[122] Nicholson KA (2009) Carers' narratives: finding dementia with Lewy bodies experiences. *Australas J Ageing* **28**, 177-181. doi:10.1111/j.1741-6612.2009.00370.x
[123] Nishio Y (2018) [Visual Impairment and False Perceptions in Dementia with Lewy Bodies]. *Brain Nerve* **70**, 889-904. doi:10.11477/mf.1416201098
[124] O'Dowd S, Schumacher J, Burn DJ, Bonanni L, Onofrj M, Thomas A, Taylor JP (2019) Fluctuating cognition in the Lewy body dementias. *Brain* **142**, 3338-3350. doi:10.1093/brain/awz235
[125] Oken RJ (1996) Lewy body diseases: possible new directions in prophylaxis and therapy. *Med Hypotheses* **46**, 222-224. doi:10.1016/s0306-9877(96)90244-3
[126] Onofrj M, Russo M, Delli Pizzi S, De Gregorio D, Inserra A, Gobbi G, Sensi SL (2023) The central role of the Thalamus in psychosis, lessons from neurodegenerative diseases and psychedelics. *Transl Psychiatry* **13**, 384. doi:10.1038/s41398-023-02691-0
[127] Onofrj M, Thomas A, Bonanni L (2007) New approaches to understanding hallucinations in Parkinson's disease: phenomenology and possible origins. *Expert Rev Neurother* **7**, 1731-1750. doi:10.1586/14737175.7.12.1731
[128] Onyike CU (2016) Psychiatric Aspects of Dementia. *Continuum (Minneap Minn)* **22**, 600-614. doi:10.1212/con.0000000000000302
[129] Pacheco C, Aguayo LG, Opazo C (2012) An extracellular mechanism that can explain the neurotoxic effects of α-synuclein aggregates in the brain. *Front Physiol* **3**, 297. doi:10.3389/fphys.2012.00297
[130] Pahwa R, Lyons KE (2010) Early diagnosis of Parkinson's disease: recommendations from diagnostic clinical guidelines. *Am J Manag Care* **16 Suppl Implications**, S94-99.

[131] Petit H, Pasquier F (1996) [Role of Alzheimer's type dementia among dementias of the elderly]. *Bull Acad Natl Med* **180**, 1715-1725; discussion 1725-1719.
[132] Pezzoli S, Cagnin A, Bandmann O, Venneri A (2017) Structural and Functional Neuroimaging of Visual Hallucinations in Lewy Body Disease: A Systematic Literature Review. *Brain Sci* **7**. doi:10.3390/brainsci7070084
[133] Prentice JL, Schaeffer MJ, Wall AK, Callahan BL (2021) A Systematic Review and Comparison of Neurocognitive Features of Late-Life Attention-Deficit/Hyperactivity Disorder and Dementia With Lewy Bodies. *J Geriatr Psychiatry Neurol* **34**, 466-481. doi:10.1177/0891988720944251
[134] Puschmann A, Bhidayasiri R, Weiner WJ (2012) Synucleinopathies from bench to bedside. *Parkinsonism Relat Disord* **18 Suppl 1**, S24-27. doi:10.1016/s1353-8020(11)70010-4
[135] Rahman MM, Mim SA, Islam MR, Parvez A, Islam F, Uddin MB, Rahaman MS, Shuvo PA, Ahmed M, Greig NH, Kamal MA (2022) Exploring the Recent Trends in Management of Dementia and Frailty: Focus on Diagnosis and Treatment. *Curr Med Chem* **29**, 5289-5314. doi:10.2174/0929867329666220408102051
[136] Rolinski M, Fox C, Maidment I, McShane R (2012) Cholinesterase inhibitors for dementia with Lewy bodies, Parkinson's disease dementia and cognitive impairment in Parkinson's disease. *Cochrane Database Syst Rev* **2012**, Cd006504. doi:10.1002/14651858.CD006504.pub2
[137] Rongve A, Aarsland D (2006) Management of Parkinson's disease dementia : practical considerations. *Drugs Aging* **23**, 807-822. doi:10.2165/00002512-200623100-00004
[138] Rosness TA, Haugen PK, Engedal K (2011) [Early onset dementia]. *Tidsskr Nor Laegeforen* **131**, 1194-1197. doi:10.4045/tidsskr.09.0845
[139] Russo M, Carrarini C, Dono F, Rispoli MG, Di Pietro M, Di Stefano V, Ferri L, Bonanni L, Sensi SL, Onofrj M (2019) The Pharmacology of Visual Hallucinations in Synucleinopathies. *Front Pharmacol* **10**, 1379. doi:10.3389/fphar.2019.01379
[140] Scherder EJ, Sergeant JA, Swaab DF (2003) Pain processing in dementia and its relation to neuropathology. *Lancet Neurol* **2**, 677-686. doi:10.1016/s1474-4422(03)00556-8
[141] Schrag A, Schott JM (2006) Epidemiological, clinical, and genetic characteristics of early-onset parkinsonism. *Lancet Neurol* **5**, 355-363. doi:10.1016/s1474-4422(06)70411-2
[142] Shah KP, Jain SB, Wadhwa R (2024) Capgras Syndrome In *StatPearls* StatPearls Publishing
Copyright © 2024, StatPearls Publishing LLC., Treasure Island (FL).
[143] Shulman KI, Hull IM, DeKoven S, Amodeo S, Mainland BJ, Herrmann N (2015) Cognitive Fluctuations and the Lucid Interval in Dementia: Implications for Testamentary Capacity. *J Am Acad Psychiatry Law* **43**, 287-292.
[144] Sian-Hulsmann J, Monoranu C, Strobel S, Riederer P (2015) Lewy Bodies: A Spectator or Salient Killer? *CNS Neurol Disord Drug Targets* **14**, 947-955. doi:10.2174/1871527314666150317225659

[145] Silber MH (2020) Parasomnias Occurring in Non-Rapid Eye Movement Sleep. *Continuum (Minneap Minn)* **26**, 946-962. doi:10.1212/con.0000000000000877
[146] Skingley A, McCue J, Vella-Burrows T (2020) Using music interventions in the care of people with dementia. *Nurs Stand* **35**, 55-60. doi:10.7748/ns.2020.e11560
[147] Snow RE, Arnold SE (1996) Psychosis in Neurodegenerative Disease. *Semin Clin Neuropsychiatry* **1**, 282-293. doi:10.1053/scnp00100282
[148] Soliman H, Coffin B, Gourcerol G (2021) Gastroparesis in Parkinson Disease: Pathophysiology, and Clinical Management. *Brain Sci* **11**. doi:10.3390/brainsci11070831
[149] Spiegel J (2010) Diagnostic and Pathophysiological Impact of Myocardial MIBG Scintigraphy in Parkinson's Disease. *Parkinsons Dis* **2010**, 295346. doi:10.4061/2010/295346
[150] Steiner B, Wolf S, Kempermann G (2006) Adult neurogenesis and neurodegenerative disease. *Regen Med* **1**, 15-28. doi:10.2217/17460751.1.1.15
[151] Swedish Council on Health Technology A (2008) SBU Systematic Reviews In *Dementia -- Caring, Ethics, Ethnical and Economical Aspects: A Systematic Review* Swedish Council on Health Technology Assessment (SBU)
Copyright © 2008 by the Swedish Council on Health Technology Assessment., Stockholm.
[152] Takamatsu Y, Fujita M, Ho GJ, Wada R, Sugama S, Takenouchi T, Waragai M, Masliah E, Hashimoto M (2018) Motor and Nonmotor Symptoms of Parkinson's Disease: Antagonistic Pleiotropy Phenomena Derived from α-Synuclein Evolvability? *Parkinsons Dis* **2018**, 5789424. doi:10.1155/2018/5789424
[153] Tan LP, Herrmann N, Mainland BJ, Shulman K (2015) Can clock drawing differentiate Alzheimer's disease from other dementias? *Int Psychogeriatr* **27**, 1649-1660. doi:10.1017/s1041610215000939
[154] Tarakad A, Jankovic J (2018) Essential Tremor and Parkinson's Disease: Exploring the Relationship. *Tremor Other Hyperkinet Mov (N Y)* **8**, 589. doi:10.7916/d8md0gvr
[155] Thomas M, Le WD (2004) Minocycline: neuroprotective mechanisms in Parkinson's disease. *Curr Pharm Des* **10**, 679-686. doi:10.2174/1381612043453162
[156] Toepper M, Falkenstein M (2019) Driving Fitness in Different Forms of Dementia: An Update. *J Am Geriatr Soc* **67**, 2186-2192. doi:10.1111/jgs.16077
[157] Ukai K (2019) Tactile hallucinations in dementia with Lewy bodies. *Psychogeriatrics* **19**, 435-439. doi:10.1111/psyg.12407
[158] Verny M, Blanc F (2019) [Lewy body dementia: therapeutic propositions according to evidence based medicine and practice]. *Geriatr Psychol Neuropsychiatr Vieil* **17**, 189-197. doi:10.1684/pnv.2019.0803
[159] Vertes AC, Beato MR, Sonne J, Khan Suheb MZ (2024) Parkinson-Plus Syndrome In *StatPearls* StatPearls Publishing
Copyright © 2024, StatPearls Publishing LLC., Treasure Island (FL).
[160] Watson GS, Leverenz JB (2010) Profile of cognitive impairment in Parkinson's disease. *Brain Pathol* **20**, 640-645. doi:10.1111/j.1750-3639.2010.00373.x
[161] Williams-Gray CH, Foltynie T, Lewis SJ, Barker RA (2006) Cognitive deficits and psychosis in Parkinson's disease: a review of pathophysiology and therapeutic options. *CNS Drugs* **20**, 477-505. doi:10.2165/00023210-200620060-00004

[162] Winslow AR, Rubinsztein DC (2011) The Parkinson disease protein α-synuclein inhibits autophagy. *Autophagy* **7**, 429-431. doi:10.4161/auto.7.4.14393

[163] Younce JR, Davis AA, Black KJ (2019) A Systematic Review and Case Series of Ziprasidone for Psychosis in Parkinson's Disease. *J Parkinsons Dis* **9**, 63-71. doi:10.3233/jpd-181448

[164] Zahodne LB, Fernandez HH (2008) Pathophysiology and treatment of psychosis in Parkinson's disease: a review. *Drugs Aging* **25**, 665-682. doi:10.2165/00002512-200825080-00004

[165] Agius LM (2003) Are tau and alpha synuclein filamentous inclusions in neurons and glia analogous to accumulation of myosin/actin filaments in myofiber hypertrophy? *Med Hypotheses* **60**, 413-417. doi:10.1016/s0306-9877(02)00416-4

[166] Amjad FS, Beinart SC (2021) Management of Neurogenic Orthostatic Hypotension in Neurodegenerative Disorders: A Collaboration Between Cardiology and Neurology. *Neurol Ther* **10**, 427-434. doi:10.1007/s40120-021-00270-3

[167] Atamanalp SS, Peksoz R, Disci E, Kartal M (2024) Comments on "Sigmoid volvulus management, only endoscopic devolvulation?". *Rev Esp Enferm Dig* **116**, 337-338. doi:10.17235/reed.2023.9751/2023

[168] Ballard C, O'Brien J, Tovee M (2002) Qualitative performance characteristics differentiate dementia with Lewy bodies and Alzheimer's disease. *J Neurol Neurosurg Psychiatry* **72**, 565-566. doi:10.1136/jnnp.72.5.565

[169] Brzezicki MA, Kobetić MD (2018) Letter to the Editor Regarding: Practical Treatment of Lewy Body Disease in the Clinic: Patient and Physician Perspectives. *Neurol Ther* **7**, 161-163. doi:10.1007/s40120-018-0098-8

[170] Collerton D, Perry E (2011) Dreaming and hallucinations - continuity or discontinuity? Perspectives from dementia with Lewy bodies. *Conscious Cogn* **20**, 1016-1020. doi:10.1016/j.concog.2011.03.024

[171] Foffani G (2024) To be or not to be hallucinating: Implications of hypnagogic/hypnopompic experiences and lucid dreaming for brain disorders. *PNAS Nexus* **3**, pgad442. doi:10.1093/pnasnexus/pgad442

[172] Jenner P, Morris HR, Robbins TW, Goedert M, Hardy J, Ben-Shlomo Y, Bolam P, Burn D, Hindle JV, Brooks D (2013) Parkinson's disease--the debate on the clinical phenomenology, aetiology, pathology and pathogenesis. *J Parkinsons Dis* **3**, 1-11. doi:10.3233/jpd-130175

[173] Jennum P, Christensen JA, Zoetmulder M (2016) Neurophysiological basis of rapid eye movement sleep behavior disorder: informing future drug development. *Nat Sci Sleep* **8**, 107-120. doi:10.2147/nss.S99240

[174] Kurlan R, Richard IH, Papka M, Marshall F (2000) Movement disorders in Alzheimer's disease: more rigidity of definitions is needed. *Mov Disord* **15**, 24-29. doi:10.1002/1531-8257(200001)15:1<24::aid-mds1006>3.0.co;2-x

[175] Manni R, Terzaghi M (2015) Sleep-disordered breathing in dementia with Lewy bodies. *Curr Neurol Neurosci Rep* **15**, 7. doi:10.1007/s11910-015-0523-7

[176] Morrow CB, Pontone GM (2024) Exploring Psychosis in Neurodegenerative Dementia: Connecting Symptoms to Neurobiology. *J Alzheimers Dis* **99**, 101-103. doi:10.3233/jad-240328

[177] Tian Y, Meng L, Zhang Z (2020) What is strain in neurodegenerative diseases? *Cell Mol Life Sci* **77**, 665-676. doi:10.1007/s00018-019-03298-9
[178] Tsukada H, Fujii H, Aihara K, Tsuda I (2015) Computational model of visual hallucination in dementia with Lewy bodies. *Neural Netw* **62**, 73-82. doi:10.1016/j.neunet.2014.09.001
[179] Agarwal K, Backler W, Bayram E, Bloom L, Boeve BF, Cha JH, Denslow M, Ferman TJ, Galasko D, Galvin JE, Gomperts SN, Irizarry MC, Kantarci K, Kaushik H, Kietlinski M, Koenig A, Leverenz JB, McKeith I, McLean PJ, Montine TJ, Moose SO, O'Brien JT, Panier V, Ramanathan S, Ringel MS, Scholz SW, Small J, Sperling RA, Taylor A, Taylor JP, Ward RA, Witten L, Hyman BT (2024) Lewy body dementia: Overcoming barriers and identifying solutions. *Alzheimers Dement* **20**, 2298-2308. doi:10.1002/alz.13674
[180] Alcolea D, Clarimón J, Carmona-Iragui M, Illán-Gala I, Morenas-Rodríguez E, Barroeta I, Ribosa-Nogué R, Sala I, Sánchez-Saudinós MB, Videla L, Subirana A, Benejam B, Valldeneu S, Fernández S, Estellés T, Altuna M, Santos-Santos M, García-Losada L, Bejanin A, Pegueroles J, Montal V, Vilaplana E, Belbin O, Dols-Icardo O, Sirisi S, Querol-Vilaseca M, Cervera-Carles L, Muñoz L, Núñez R, Torres S, Camacho MV, Carrió I, Giménez S, Delaby C, Rojas-Garcia R, Turon-Sans J, Pagonabarraga J, Jiménez A, Blesa R, Fortea J, Lleó A (2019) The Sant Pau Initiative on Neurodegeneration (SPIN) cohort: A data set for biomarker discovery and validation in neurodegenerative disorders. *Alzheimers Dement (N Y)* **5**, 597-609. doi:10.1016/j.trci.2019.09.005
[181] Collerton D, Barnes J, Diederich NJ, Dudley R, Ffytche D, Friston K, Goetz CG, Goldman JG, Jardri R, Kulisevsky J, Lewis SJG, Nara S, O'Callaghan C, Onofrj M, Pagonabarraga J, Parr T, Shine JM, Stebbins G, Taylor JP, Tsuda I, Weil RS (2023) Understanding visual hallucinations: A new synthesis. *Neurosci Biobehav Rev* **150**, 105208. doi:10.1016/j.neubiorev.2023.105208
[182] Goldman JG, Boeve BF, Armstrong MJ, Galasko DR, Galvin JE, Irwin DJ, Leverenz JB, Marder K, Abler V, Biglan K, Irizarry MC, Keller B, Lai R, Munsie L, Belleville M, Chaney O, Richard I, Taylor A, Graham T (2021) Lewy Body Dementia Association's Industry Advisory Council: proceedings of the second annual meeting. *Alzheimers Res Ther* **13**, 124. doi:10.1186/s13195-021-00868-7
[183] Isik AT, Danyeli AE, Kaya D, Soysal P, Karabay N, Gokden M (2020) The importance of brain banking for dementia practice: the first experience of Turkey. *Cell Tissue Bank* **21**, 367-375. doi:10.1007/s10561-020-09835-2
[184] Lee JM, Derkinderen P, Kordower JH, Freeman R, Munoz DG, Kremer T, Zago W, Hutten SJ, Adler CH, Serrano GE, Beach TG (2017) The Search for a Peripheral Biopsy Indicator of α-Synuclein Pathology for Parkinson Disease. *J Neuropathol Exp Neurol* **76**, 2-15. doi:10.1093/jnen/nlw103
[185] Mirra SS (1997) Neuropathological assessment of Alzheimer's disease: the experience of the Consortium to Establish a Registry for Alzheimer's Disease. *Int Psychogeriatr* **9 Suppl 1**, 263-268; discussion 269-272. doi:10.1017/s1041610297004985

[186] Petersen RC, Weintraub S, Sabbagh M, Karlawish J, Adler CH, Dilworth-Anderson P, Frank L, Huling Hummel C, Taylor A (2023) A New Framework for Dementia Nomenclature. *JAMA Neurol* **80**, 1364-1370. doi:10.1001/jamaneurol.2023.3664

[187] Verghese J, Malik R, Zwerling J (2016) Montefiore-Einstein Center for the Aging Brain: Preliminary Data. *J Am Geriatr Soc* **64**, 2374-2377. doi:10.1111/jgs.14473

[188] (2018) National Institute for Health and Care Excellence: Guidelines In *Dementia: Assessment, management and support for people living with dementia and their carers* National Institute for Health and Care Excellence (NICE)
Copyright © NICE 2018., London.

[189] Howell M, Avidan AY, Foldvary-Schaefer N, Malkani RG, During EH, Roland JP, McCarter SJ, Zak RS, Carandang G, Kazmi U, Ramar K (2023) Management of REM sleep behavior disorder: an American Academy of Sleep Medicine clinical practice guideline. *J Clin Sleep Med* **19**, 759-768. doi:10.5664/jcsm.10424

[190] Monteiro A, Velon AG, Rodrigues AM, Oliveira A, Valadas A, Nóbrega C, Cruto C, Neutel D, Simões do Couto F, Morgado J, Cerejeira J, Ruano L, Gago M, Grunho M, Tábuas-Pereira M, Taipa R, Moiron Simões R, Araújo R, Barreto R, Rocha S, Massano J (2020) [Portuguese Consensus on the Diagnosis and Management of Lewy Body Dementia (PORTUCALE)]. *Acta Med Port* **33**, 844-854. doi:10.20344/amp.13696

[191] Seraji-Bzorgzad N, Paulson H, Heidebrink J (2019) Neurologic examination in the elderly. *Handb Clin Neurol* **167**, 73-88. doi:10.1016/b978-0-12-804766-8.00005-4

[192] Abadir A, Dalton R, Zheng W, Pincavitch J, Tripathi R (2022) Neuroleptic Sensitivity in Dementia with Lewy Body and Use of Pimavanserin in an Inpatient Setting: A Case Report. *Am J Case Rep* **23**, e937397. doi:10.12659/ajcr.937397

[193] Abbate C, Trimarchi PD, Inglese S, Viti N, Cantatore A, De Agostini L, Pirri F, Marino L, Bagarolo R, Mari D (2014) Preclinical polymodal hallucinations for 13 years before dementia with Lewy bodies. *Behav Neurol* **2014**, 694296. doi:10.1155/2014/694296

[194] Abe K, Chiba Y (2019) A case of treatable dementia with Lewy bodies remarkably improved by immunotherapy. *J Neuroimmunol* **330**, 35-37. doi:10.1016/j.jneuroim.2019.02.003

[195] Aprahamian I, Yassuda MS, Martinelli JE (2015) Somatoform and Conversion Disorder Preceding Lewy Body Dementia: A Newly Described Phenomenological Manifestation of the Disease. *J Am Geriatr Soc* **63**, 1967-1969. doi:10.1111/jgs.13636

[196] Carlisle TC, Birlea M, Restrepo D, Filley CM (2023) Headache-Associated Phantosmia as a Harbinger of Lewy Body Dementia. *J Neuropsychiatry Clin Neurosci* **35**, 92-97. doi:10.1176/appi.neuropsych.21110265

[197] Denson MA, Wszolek ZK, Pfeiffer RF, Wszolek EK, Paschall TM, McComb RD (1997) Familial parkinsonism, dementia, and Lewy body disease: study of family G. *Ann Neurol* **42**, 638-643. doi:10.1002/ana.410420415

[198] Fiacconi CM, Barkley V, Finger EC, Carson N, Duke D, Rosenbaum RS, Gilboa A, Köhler S (2014) Nature and extent of person recognition impairments associated with Capgras syndrome in Lewy body dementia. *Front Hum Neurosci* **8**, 726. doi:10.3389/fnhum.2014.00726

[199] Fujishiro H (2014) Effects of gabapentin enacarbil on restless legs syndrome and leg pain in dementia with Lewy bodies. *Psychogeriatrics* **14**, 132-134. doi:10.1111/psyg.12043
[200] Hamdy RC, Kinser A, Kendall-Wilson T, Depelteau A, Copeland R, Whalen K, Culp J (2018) Visual Hallucinations and Paranoid Delusions. *Gerontol Geriatr Med* **4**, 2333721418777086. doi:10.1177/2333721418777086
[201] Heyman I, Brorsson A, Persson T, Londos E (2023) Pacemaker Implants and Their Influence on the Daily Life of Patients with Dementia with Lewy Bodies: A Qualitative Case Study. *Neurol Ther* **12**, 1359-1373. doi:10.1007/s40120-023-00513-5
[202] Ishikawa A, Takahashi H, Tanaka H, Hayashi T, Tsuji S (1997) Clinical features of familial diffuse Lewy body disease. *Eur Neurol* **38 Suppl 1**, 34-38. doi:10.1159/000113459
[203] Ishimaru D, Kanemoto H, Hotta M, Nagata Y, Koizumi F, Satake Y, Taomoto D, Ikeda M (2024) Case report: Environmental adjustment for visual hallucinations in dementia with Lewy bodies based on photo assessment of the living environment. *Front Psychiatry* **15**, 1283156. doi:10.3389/fpsyt.2024.1283156
[204] Lindeberg S, Müller N, Samuelsson C (2023) Conversations in dementia with Lewy bodies: Resources and barriers in communication. *Int J Lang Commun Disord* **58**, 419-432. doi:10.1111/1460-6984.12799
[205] Londos E (2018) Practical Treatment of Lewy Body Disease in the Clinic: Patient and Physician Perspectives. *Neurol Ther* **7**, 13-22. doi:10.1007/s40120-017-0090-8
[206] Londos E, Hansson O, Rosén I, Englund E (2019) Extreme sleep pattern in Lewy body dementia: a hypothalamic matter? *BMJ Case Rep* **12**. doi:10.1136/bcr-2018-228177
[207] Morita S, Miwa H, Kondo T (2004) [A patient with probable dementia with Lewy bodies, who showed catatonia induced by donepezil: a case report]. *No To Shinkei* **56**, 881-884.
[208] Okuma Y, Tanaka S, Nomura Y, Mori H, Yan H, Shirai T, Kondo T, Segawa M, Mizuno Y (1996) [A 63-year-old woman with muscle weakness, myotonia, and parkinsonism]. *No To Shinkei* **48**, 287-297.
[209] Poletti M, Lucetti C, Del Dotto P, Borelli P, Baldacci F, Logi C, Bonuccelli U (2012) The "closing-in" phenomenon in Parkinson's disease dementia and lewy-body dementia. *J Neuropsychiatry Clin Neurosci* **24**, E38-39. doi:10.1176/appi.neuropsych.11110327
[210] Rasmussen KG, Jr., Russell JC, Kung S, Rummans TA, Rae-Stuart E, O'Connor MK (2003) Electroconvulsive therapy for patients with major depression and probable Lewy body dementia. *J ect* **19**, 103-109. doi:10.1097/00124509-200306000-00009
[211] Richard IH, Justus AW, Greig NH, Marshall F, Kurlan R (2002) Worsening of motor function and mood in a patient with Parkinson's disease after pharmacologic challenge with oral rivastigmine. *Clin Neuropharmacol* **25**, 296-299. doi:10.1097/00002826-200211000-00002
[212] Rothenberg KG, McRae SG, Dominguez-Colman LM, Shutes-David A, Tsuang DW (2023) Pimavanserin Treatment for Psychosis in Patients with Dementia with Lewy Bodies: A Case Series. *Am J Case Rep* **24**, e939806. doi:10.12659/ajcr.939806

[213] Suzuki A, Ikebe S, Komatsuzaki Y, Takanashi M, Mori H, Hattori N, Mizuno Y (2001) [A 64-year-old man with parkinsonism as an initial symptom followed by dementia associated with marked abnormal behaviours]. *No To Shinkei* **53**, 1075-1087.
[214] Takanashi M, Urabe T, Ohta S, Hamano Y, Mori H, Shirai T, Kondo T, Mizuno Y (1999) [A 73-year-old woman with familial Parkinson's disease]. *No To Shinkei* **51**, 1087-1096.
[215] Ukai K (2022) A proposal for a new concept in psychiatric phenomenology: The "Yume-Utsutsu" (dreamy or half-asleep) phenomenon in patients with dementia with Lewy bodies. *PCN Rep* **1**, e50. doi:10.1002/pcn5.50
[216] van der Meer S, Keizer B (2020) [Euthanasia in patients with dementia]. *Ned Tijdschr Geneeskd* **164**.
[217] von Siebenthal A, Descloux V, Borgognon C, Massardi T, Zumbach S (2021) Evolution of Capgras syndrome in neurodegenerative disease: the multiplication phenomenon. *Neurocase* **27**, 160-164. doi:10.1080/13554794.2021.1905850
[218] Watanabe M, Araki W, Takao C, Maeda C, Tominaga R, Kimura Y, Nayanar G, Tu TTH, Asada T, Toyofuku A (2023) A case with burning mouth syndrome followed by dementia with Lewy bodies: a case report. *Front Psychiatry* **14**, 1329171. doi:10.3389/fpsyt.2023.1329171
[219] Yamagata B, Kobayashi H, Yamamoto H, Mimura M (2014) Visual text hallucinations of thoughts in an alexic woman. *J Neurol Sci* **339**, 226-228. doi:10.1016/j.jns.2014.01.036
[220] Yamamura Y, Arihiro K, Kohriyama T, Nakamura S (1993) [Early-onset parkinsonism with diurnal fluctuation--clinical and pathological studies]. *Rinsho Shinkeigaku* **33**, 491-496.
[221] Yoshimura N, Yoshimura I, Asada M, Hayashi S, Fukushima Y, Sato T, Kudo H (1988) Juvenile Parkinson's disease with widespread Lewy bodies in the brain. *Acta Neuropathol* **77**, 213-218. doi:10.1007/bf00687434
[222] Armstrong MJ, Dai Y, Sovich K, LaBarre B, Paulson HL, Maixner SM, Fields JA, Lunde AM, Forsberg LK, Boeve BF, Manning CA, Galvin JE, Taylor AS, Li Z (2024) Caregiver Experiences and Burden in Moderate-Advanced Dementia With Lewy Bodies. *Neurol Clin Pract* **14**, e200292. doi:10.1212/cpj.0000000000200292
[223] Armstrong MJ, LaBarre B, Sovich K, Maixner SM, Paulson HL, Manning C, Fields JA, Lunde A, Forsberg L, Boeve BF, Galvin JE, Taylor AS, Li Z (2024) Patient- and proxy-reported quality of life in advanced dementia with Lewy bodies. *Alzheimers Dement* **20**, 2719-2730. doi:10.1002/alz.13745
[224] Atee M, Morris T, Macfarlane S, Cunningham C (2021) Pain in Dementia: Prevalence and Association With Neuropsychiatric Behaviors. *J Pain Symptom Manage* **61**, 1215-1226. doi:10.1016/j.jpainsymman.2020.10.011
[225] Ballard C, Patel A, Oyebode F, Wilcock G (1996) Cognitive decline in patients with Alzheimer's disease, vascular dementia and senile dementia of Lewy body type. *Age Ageing* **25**, 209-213. doi:10.1093/ageing/25.3.209
[226] Ballard CG, Aarsland D, McKeith I, O'Brien J, Gray A, Cormack F, Burn D, Cassidy T, Starfeldt R, Larsen JP, Brown R, Tovee M (2002) Fluctuations in attention: PD

dementia vs DLB with parkinsonism. *Neurology* **59**, 1714-1720. doi:10.1212/01.wnl.0000036908.39696.fd
[227] Ballard CG, O'Brien J, Lowery K, Ayre GA, Harrison R, Perry R, Ince P, Neill D, McKeith IG (1998) A prospective study of dementia with Lewy bodies. *Age Ageing* **27**, 631-636. doi:10.1093/ageing/27.5.631
[228] Beagle AJ, Darwish SM, Ranasinghe KG, La AL, Karageorgiou E, Vossel KA (2017) Relative Incidence of Seizures and Myoclonus in Alzheimer's Disease, Dementia with Lewy Bodies, and Frontotemporal Dementia. *J Alzheimers Dis* **60**, 211-223. doi:10.3233/jad-170031
[229] Blanke O, Bernasconi F, Potheegadoo J (2023) Phantom Boarder Relates to Experimentally-Induced Presence Hallucinations in Parkinson's Disease. *Mov Disord Clin Pract* **10**, 617-624. doi:10.1002/mdc3.13684
[230] Bräuer S, Rossi M, Sajapin J, Henle T, Gasser T, Parchi P, Brockmann K, Falkenburger BH (2023) Kinetic parameters of alpha-synuclein seed amplification assay correlate with cognitive impairment in patients with Lewy body disorders. *Acta Neuropathol Commun* **11**, 162. doi:10.1186/s40478-023-01653-3
[231] Brodaty H, Connors MH, Xu J, Woodward M, Ames D (2015) The course of neuropsychiatric symptoms in dementia: a 3-year longitudinal study. *J Am Med Dir Assoc* **16**, 380-387. doi:10.1016/j.jamda.2014.12.018
[232] Cagnin A, Bussè C, Gardini S, Jelcic N, Guzzo C, Gnoato F, Mitolo M, Ermani M, Caffarra P (2015) Clinical and Cognitive Phenotype of Mild Cognitive Impairment Evolving to Dementia with Lewy Bodies. *Dement Geriatr Cogn Dis Extra* **5**, 442-449. doi:10.1159/000441184
[233] Cagnin A, Bussè C, Jelcic N, Gnoato F, Mitolo M, Caffarra P (2015) High specificity of MMSE pentagon scoring for diagnosis of prodromal dementia with Lewy bodies. *Parkinsonism Relat Disord* **21**, 303-305. doi:10.1016/j.parkreldis.2014.12.007
[234] Calil V, Silveira de Souza A, Sudo FK, Santiago-Bravo G, Assunção N, Drummond C, Rodrigues F, Soares R, Oliveira N, Teldeschi A, Bernardes G, Lima G, Lima C, Lima MA, Mattos P (2021) Anosognosia for memory in dementia with Lewy bodies compared with Alzheimer's disease. *Int J Geriatr Psychiatry* **36**, 1059-1064. doi:10.1002/gps.5521
[235] Chandra SR, Issac TG, Abbas MM (2015) Apraxias in neurodegenerative dementias. *Indian J Psychol Med* **37**, 42-47. doi:10.4103/0253-7176.150817
[236] Chen G, Liu S, Wu H, Gan J, Wang X, Ji Y (2023) Analysis of clinical characteristics of mirror and TV signs in Alzheimer's disease and dementia with Lewy bodies. *J Int Med Res* **51**, 3000605231156098. doi:10.1177/03000605231156098
[237] Chiu PY, Hung GU, Wei CY, Tzeng RC, Pai MC (2020) Freezing of Speech Single Questionnaire as a Screening Tool for Cognitive Dysfunction in Patients With Dementia With Lewy Bodies. *Front Aging Neurosci* **12**, 65. doi:10.3389/fnagi.2020.00065
[238] Chiu PY, Teng PR, Wei CY, Wang CW, Tsai CT (2018) Gender difference in the association and presentation of visual hallucinations in dementia with Lewy bodies: a cross-sectional study. *Int J Geriatr Psychiatry* **33**, 193-199. doi:10.1002/gps.4706

[239] Cotta Ramusino M, Perini G, Vaghi G, Dal Fabbro B, Capelli M, Picascia M, Franciotta D, Farina L, Ballante E, Costa A (2021) Correlation of Frontal Atrophy and CSF Tau Levels With Neuropsychiatric Symptoms in Patients With Cognitive Impairment: A Memory Clinic Experience. *Front Aging Neurosci* **13**, 595758. doi:10.3389/fnagi.2021.595758

[240] de Boysson C, Belleville S, Phillips NA, Johns EK, Goupil D, Souchay C, Bouchard R, Chertkow H (2011) False recognition in Lewy-body disease and frontotemporal dementia. *Brain Cogn* **75**, 111-118. doi:10.1016/j.bandc.2010.10.011

[241] Doubleday EK, Snowden JS, Varma AR, Neary D (2002) Qualitative performance characteristics differentiate dementia with Lewy bodies and Alzheimer's disease. *J Neurol Neurosurg Psychiatry* **72**, 602-607. doi:10.1136/jnnp.72.5.602

[242] Elder GJ, Colloby SJ, Lett DJ, O'Brien JT, Anderson KN, Burn DJ, McKeith IG, Taylor JP (2016) Depressive symptoms are associated with daytime sleepiness and subjective sleep quality in dementia with Lewy bodies. *Int J Geriatr Psychiatry* **31**, 765-770. doi:10.1002/gps.4389

[243] Falque A, Jordanis M, Landré L, Loureiro de Sousa P, Mondino M, Furcieri E, Blanc F (2022) Neural basis of impaired narrative discourse comprehension in prodromal and mild dementia with lewy bodies. *Front Aging Neurosci* **14**, 939973. doi:10.3389/fnagi.2022.939973

[244] Ferman TJ, Arvanitakis Z, Fujishiro H, Duara R, Parfitt F, Purdy M, Waters C, Barker W, Graff-Radford NR, Dickson DW (2013) Pathology and temporal onset of visual hallucinations, misperceptions and family misidentification distinguishes dementia with Lewy bodies from Alzheimer's disease. *Parkinsonism Relat Disord* **19**, 227-231. doi:10.1016/j.parkreldis.2012.10.013

[245] Fernández-Arcos A, Morenas-Rodríguez E, Santamaria J, Sánchez-Valle R, Lladó A, Gaig C, Lleó A, Iranzo A (2019) Clinical and video-polysomnographic analysis of rapid eye movement sleep behavior disorder and other sleep disturbances in dementia with Lewy bodies. *Sleep* **42**. doi:10.1093/sleep/zsz086

[246] Fleisher JE, Moshkovich A, Levin M, Akram F, Hess SP, Suresh M, Bentley CD, Manak T, Mattke M, Miller A, Pennsyl CE, Pierce CM, Voss M, Taylor A, Schroeder K, Chodosh J (2023) Family Caregiver Comorbidities in Lewy Body Dementia Versus Alzheimer Disease and Associated Disorders. *Alzheimer Dis Assoc Disord* **37**, 42-49. doi:10.1097/wad.0000000000000535

[247] Gallop K, Pham N, Maclaine G, Saunders E, Black B, Hubig L, Acaster S (2023) Health-related quality-of-life and burden for caregivers of individuals with neurogenic orthostatic hypotension. *Neurodegener Dis Manag* **13**, 35-45. doi:10.2217/nmt-2022-0015

[248] Galvin JE, Malcom H, Johnson D, Morris JC (2007) Personality traits distinguishing dementia with Lewy bodies from Alzheimer disease. *Neurology* **68**, 1895-1901. doi:10.1212/01.wnl.0000263131.80945.ad

[249] Gibson LL, Grinberg LT, Ffytche D, Leite REP, Rodriguez RD, Ferretti-Rebustini REL, Pasqualucci CA, Nitrini R, Jacob-Filho W, Aarsland D, Suemoto CK (2023) Neuropathological correlates of neuropsychiatric symptoms in dementia. *Alzheimers Dement* **19**, 1372-1382. doi:10.1002/alz.12765

[250] Golden EC, Josephs KA (2015) Minds on replay: musical hallucinations and their relationship to neurological disease. *Brain* **138**, 3793-3802. doi:10.1093/brain/awv286
[251] Hamilton JM, Salmon DP, Galasko D, Raman R, Emond J, Hansen LA, Masliah E, Thal LJ (2008) Visuospatial deficits predict rate of cognitive decline in autopsy-verified dementia with Lewy bodies. *Neuropsychology* **22**, 729-737. doi:10.1037/a0012949
[252] Harciarek M, Kertesz A (2008) The prevalence of misidentification syndromes in neurodegenerative diseases. *Alzheimer Dis Assoc Disord* **22**, 163-169. doi:10.1097/WAD.0b013e3181641341
[253] Hashimoto M, Sakamoto S, Ikeda M (2015) Clinical features of delusional jealousy in elderly patients with dementia. *J Clin Psychiatry* **76**, 691-695. doi:10.4088/JCP.14m09018
[254] Hirono N, Kobayashi H, Mori E (1998) [Caregiver burden in dementia: evaluation with a Japanese version of the Zarit caregiver burden interview]. *No To Shinkei* **50**, 561-567.
[255] Hirono N, Mori E, Imamura T, Shimomura T, Hashimoto M (1998) [Neuropsychiatric features in Dementia with Lewy bodies and Alzheimer's disease]. *No To Shinkei* **50**, 45-49.
[256] Huang WC, Chang MC, Wang WF, Jhang KM (2021) A Comparison of Caregiver Burden for Different Types of Dementia: An 18-Month Retrospective Cohort Study. *Front Psychol* **12**, 798315. doi:10.3389/fpsyg.2021.798315
[257] Iaccarino L, Marelli S, Iannaccone S, Magnani G, Ferini-Strambi L, Perani D (2016) Severe Brain Metabolic Decreases Associated with REM Sleep Behavior Disorder in Dementia with Lewy Bodies. *J Alzheimers Dis* **52**, 989-997. doi:10.3233/jad-151000
[258] Kanemoto H, Sato S, Satake Y, Koizumi F, Taomoto D, Kanda A, Wada T, Yoshiyama K, Ikeda M (2021) Impact of Behavioral and Psychological Symptoms on Caregiver Burden in Patients With Dementia With Lewy Bodies. *Front Psychiatry* **12**, 753864. doi:10.3389/fpsyt.2021.753864
[259] Kawano Y, Terada S, Takenoshita S, Hayashi S, Oshima Y, Miki T, Yokota O, Yamada N (2020) Patient affect and caregiver burden in dementia. *Psychogeriatrics* **20**, 189-195. doi:10.1111/psyg.12487
[260] Lee DR, McKeith I, Mosimann U, Ghosh-Nodyal A, Thomas AJ (2013) Examining carer stress in dementia: the role of subtype diagnosis and neuropsychiatric symptoms. *Int J Geriatr Psychiatry* **28**, 135-141. doi:10.1002/gps.3799
[261] Leggett AN, Zarit S, Taylor A, Galvin JE (2011) Stress and burden among caregivers of patients with Lewy body dementia. *Gerontologist* **51**, 76-85. doi:10.1093/geront/gnq055
[262] Leroi I, Vatter S, Carter LA, Smith SJ, Orgeta V, Poliakoff E, Silverdale MA, Raw J, Ahearn DJ, Taylor C, Rodda J, Abdel-Ghany T, McCormick SA (2019) Parkinson's-adapted cognitive stimulation therapy: a pilot randomized controlled clinical trial. *Ther Adv Neurol Disord* **12**, 1756286419852217. doi:10.1177/1756286419852217
[263] Lin W, Xie YC, Cheng PY, Dong LY, Hung GU, Chiu PY (2018) Association of visual hallucinations with very mild degenerative dementia due to dementia with Lewy bodies. *PLoS One* **13**, e0205909. doi:10.1371/journal.pone.0205909

[264] Liu S, Jin Y, Shi Z, Huo YR, Guan Y, Liu M, Liu S, Ji Y (2017) The effects of behavioral and psychological symptoms on caregiver burden in frontotemporal dementia, Lewy body dementia, and Alzheimer's disease: clinical experience in China. *Aging Ment Health* **21**, 651-657. doi:10.1080/13607863.2016.1146871

[265] Liu S, Liu J, Wang XD, Shi Z, Zhou Y, Li J, Yu T, Ji Y (2018) Caregiver burden, sleep quality, depression, and anxiety in dementia caregivers: a comparison of frontotemporal lobar degeneration, dementia with Lewy bodies, and Alzheimer's disease. *Int Psychogeriatr* **30**, 1131-1138. doi:10.1017/s1041610217002630

[266] Lomax CL, Brown RG, Howard RJ (2004) Measuring disability in patients with neurodegenerative disease using the 'Yesterday Interview'. *Int J Geriatr Psychiatry* **19**, 1058-1064. doi:10.1002/gps.1210

[267] Lowery K, Mynt P, Aisbett J, Dixon T, O'Brien J, Ballard C (2000) Depression in the carers of dementia sufferers: a comparison of the carers of patients suffering from dementia with Lewy bodies and the carers of patients with Alzheimer's disease. *J Affect Disord* **59**, 61-65. doi:10.1016/s0165-0327(99)00123-8

[268] Marquié M, Castilla-Martí M, Valero S, Martínez J, Sánchez D, Hernández I, Rosende-Roca M, Vargas L, Mauleón A, Rodríguez-Gómez O, Abdelnour C, Gil S, Santos-Santos MA, Alegret M, Espinosa A, Ortega G, Pérez-Cordón A, Sanabria Á, Roberto N, Moreno-Grau S, de Rojas I, Simó R, Ciudin A, Hernández C, Orellana A, Monté-Rubio G, Benaque A, Ruiz A, Tárraga L, Boada M (2019) Visual impairment in aging and cognitive decline: experience in a Memory Clinic. *Sci Rep* **9**, 8698. doi:10.1038/s41598-019-45055-9

[269] Matar E, Phillips JR, Martens KAE, Halliday GM, Lewis SJG (2019) Impaired Color Discrimination-A Specific Marker of Hallucinations in Lewy Body Disorders. *J Geriatr Psychiatry Neurol* **32**, 257-264. doi:10.1177/0891988719845501

[270] Mendes A, Noblet V, Mondino M, Loureiro de Sousa P, Manji S, Archenault A, Casanovas M, Bousiges O, Philippi N, Baloglu S, Rauch L, Cretin B, Demuynck C, Martin-Hunyadi C, Blanc F (2021) Association of cerebral microbleeds with cerebrospinal fluid Alzheimer-biomarkers and clinical symptoms in early dementia with Lewy bodies. *Int J Geriatr Psychiatry* **36**, 851-857. doi:10.1002/gps.5485

[271] Montesinos R, Custodio B, Malaga M, Chambergo-Michilot D, Verastegui-Aranda G, Agüero K, Alejos-Zirena J, Andamayo-Villalba L, Seminario GW, Custodio N (2024) Influence of Behavioral and Psychological Symptoms on Caregiver Burden for Different Types of Dementia: Clinical Experience in Lima, Peru. *Dement Geriatr Cogn Disord*, 1-8. doi:10.1159/000539335

[272] Mori E, Ikeda M, Iseki E, Katayama S, Nagahama Y, Ohdake M, Takase T (2024) Efficacy and safety of donepezil in patients with dementia with Lewy bodies: results from a 12-week multicentre, randomised, double-blind, and placebo-controlled phase IV study. *Psychogeriatrics* **24**, 542-554. doi:10.1111/psyg.13091

[273] Mori E, Ikeda M, Ohdake M (2024) Donepezil for dementia with Lewy bodies: meta-analysis of multicentre, randomised, double-blind, placebo-controlled phase II, III, and, IV studies. *Psychogeriatrics* **24**, 589-596. doi:10.1111/psyg.13101

[274] Mosimann UP, Rowan EN, Partington CE, Collerton D, Littlewood E, O'Brien JT, Burn DJ, McKeith IG (2006) Characteristics of visual hallucinations in Parkinson disease

dementia and dementia with lewy bodies. *Am J Geriatr Psychiatry* **14**, 153-160. doi:10.1097/01.Jgp.0000192480.89813.80
[275] Murayama N, Masubuchi Y, Kimura A, Uchiyama K, Yamagata M, Ota K, Iseki E (2023) A simple method to evaluate the pentagon copy test of the Mini-Mental State Examination for the differentiation of dementia with Lewy bodies. *Appl Neuropsychol Adult*, 1-7. doi:10.1080/23279095.2023.2200948
[276] Onofrj M, Bonanni L, Manzoli L, Thomas A (2010) Cohort study on somatoform disorders in Parkinson disease and dementia with Lewy bodies. *Neurology* **74**, 1598-1606. doi:10.1212/WNL.0b013e3181df09dd
[277] Onofrj M, Thomas A, Tiraboschi P, Wenning G, Gambi F, Sepede G, Di Giannantonio M, Di Carmine C, Monaco D, Maruotti V, Ciccocioppo F, D'Amico MC, Bonanni L (2011) Updates on Somatoform Disorders (SFMD) in Parkinson's Disease and Dementia with Lewy Bodies and discussion of phenomenology. *J Neurol Sci* **310**, 166-171. doi:10.1016/j.jns.2011.07.010
[278] Onofrj M, Varanese S, Bonanni L, Taylor JP, Antonini A, Valente EM, Petrucci S, Stocchi F, Thomas A, Perfetti B (2013) Cohort study of prevalence and phenomenology of tremor in dementia with Lewy bodies. *J Neurol* **260**, 1731-1742. doi:10.1007/s00415-013-6853-y
[279] Pai MC, Yang CJ, Fan SY (2021) Time Perception in Prodromal Alzheimer's Dementia and in Prodromal Dementia With Lewy Bodies. *Front Psychiatry* **12**, 728344. doi:10.3389/fpsyt.2021.728344
[280] Palermo G, Frosini D, Corsi A, Giuntini M, Mazzucchi S, Del Prete E, Bonuccelli U, Ceravolo R (2019) Freezing of gait and dementia in parkinsonism: A retrospective case-control study. *Brain Behav* **9**, e01247. doi:10.1002/brb3.1247
[281] Park J, Galvin JE (2021) Pre-Loss Grief in Caregivers of Older Adults with Dementia with Lewy Bodies. *J Alzheimers Dis* **82**, 1847-1859. doi:10.3233/jad-210616
[282] Perini G, Carlini A, Pomati S, Alberoni M, Mariani C, Nemni R, Farina E (2016) Misidentification Delusions: Prevalence in Different Types of Dementia and Validation of a Structured Questionnaire. *Alzheimer Dis Assoc Disord* **30**, 331-337. doi:10.1097/wad.0000000000000141
[283] Perri R, Fadda L, Caltagirone C, Carlesimo GA (2013) Word list and story recall elicit different patterns of memory deficit in patients with Alzheimer's disease, frontotemporal dementia, subcortical ischemic vascular disease, and Lewy body dementia. *J Alzheimers Dis* **37**, 99-107. doi:10.3233/jad-130347
[284] Phillipps C, Kemp J, Jacob C, Veronneau A, Albasser T, Philippi N, Cretin B, Bernard F, Blanc F (2016) Comparative study of false memory in dementia with Lewy bodies and Alzheimer's disease. *Geriatr Psychol Neuropsychiatr Vieil* **14**, 332-340. doi:10.1684/pnv.2016.0620
[285] Puntambekar I, J AF (2023) Neuropsychological correlates of visual hallucinatory phenomena in Lewy body disease. *Int J Geriatr Psychiatry* **38**, e5950. doi:10.1002/gps.5950
[286] Querry M, Botzung A, Cretin B, Demuynck C, Muller C, Ravier A, Schorr B, Mondino M, Sanna L, de Sousa PL, Philippi N, Blanc F (2024) Neuroanatomical substrates of

depression in dementia with Lewy bodies and Alzheimer's disease. *Geroscience*. doi:10.1007/s11357-024-01190-4
[287] Rainero I, Bruni AC, Marra C, Cagnin A, Bonanni L, Cupidi C, Laganà V, Rubino E, Vacca A, Di Lorenzo R, Provero P, Isella V, Vanacore N, Agosta F, Appollonio I, Caffarra P, Bussè C, Sambati R, Quaranta D, Guglielmi V, Logroscino G, Filippi M, Tedeschi G, Ferrarese C (2020) The Impact of COVID-19 Quarantine on Patients With Dementia and Family Caregivers: A Nation-Wide Survey. *Front Aging Neurosci* **12**, 625781. doi:10.3389/fnagi.2020.625781
[288] Rigby T, Ashwill RT, Johnson DK, Galvin JE (2019) Differences in the Experience of Caregiving Between Spouse and Adult Child Caregivers in Dementia With Lewy Bodies. *Innov Aging* **3**, igz027. doi:10.1093/geroni/igz027
[289] Rigby T, Johnson DK, Taylor A, Galvin JE (2021) Comparison of the Caregiving Experience of Grief, Burden, and Quality of Life in Dementia with Lewy Bodies, Alzheimer's Disease, and Parkinson's Disease Dementia. *J Alzheimers Dis* **80**, 421-432. doi:10.3233/jad-201326
[290] Rongve A, Boeve BF, Aarsland D (2010) Frequency and correlates of caregiver-reported sleep disturbances in a sample of persons with early dementia. *J Am Geriatr Soc* **58**, 480-486. doi:10.1111/j.1532-5415.2010.02733.x
[291] Roqué M, Salvà A, Vellas B (2013) Malnutrition in community-dwelling adults with dementia (NutriAlz Trial). *J Nutr Health Aging* **17**, 295-299. doi:10.1007/s12603-012-0401-9
[292] Sabatini S, Martyr A, Ukoumunne OC, Ballard C, Collins R, Pentecost C, Rusted JM, Quinn C, Anstey KJ, Kim S, Corbett A, Brooker H, Clare L (2022) Attitudes toward own aging and cognition among individuals living with and without dementia: findings from the IDEAL programme and the PROTECT study. *BMC Geriatr* **22**, 641. doi:10.1186/s12877-022-03336-5
[293] Schutte MJL, Linszen MMJ, Marschall TM, Ffytche DH, Koops S, van Dellen E, Heringa SM, Slooter AJC, Teunisse R, van den Heuvel OA, Lemstra AW, Foncke EMJ, Slotema CW, de Jong J, Rossell SL, Sommer IEC (2020) Hallucinations and other psychotic experiences across diagnoses: A comparison of phenomenological features. *Psychiatry Res* **292**, 113314. doi:10.1016/j.psychres.2020.113314
[294] Shinagawa S, Hashimoto M, Yamakage H, Toya S, Ikeda M (2024) Eating problems in people with dementia with Lewy bodies: Associations with various symptoms and the physician's understanding. *Int Psychogeriatr*, 1-11. doi:10.1017/s1041610224000346
[295] Soennesyn H, Oppedal K, Greve OJ, Fritze F, Auestad BH, Nore SP, Beyer MK, Aarsland D (2012) White matter hyperintensities and the course of depressive symptoms in elderly people with mild dementia. *Dement Geriatr Cogn Dis Extra* **2**, 97-111. doi:10.1159/000335497
[296] Suárez-González A, Serrano-Pozo A, Arroyo-Anlló EM, Franco-Macías E, Polo J, García-Solís D, Gil-Néciga E (2014) Utility of neuropsychiatric tools in the differential diagnosis of dementia with Lewy bodies and Alzheimer's disease: quantitative and qualitative findings. *Int Psychogeriatr* **26**, 453-461. doi:10.1017/s1041610213002068

[297] Tarolli CG, Zimmerman GA, Goldenthal S, Feldman B, Berk S, Siddiqi B, Kopil CM, Chowdhury S, Biglan KM, Dorsey ER, Adams JL (2020) Video research visits for atypical parkinsonian syndromes among Fox Trial Finder participants. *Neurol Clin Pract* **10**, 7-14. doi:10.1212/cpj.0000000000000680

[298] Terzaghi M, Arnaldi D, Rizzetti MC, Minafra B, Cremascoli R, Rustioni V, Zangaglia R, Pasotti C, Sinforiani E, Pacchetti C, Manni R (2013) Analysis of video-polysomnographic sleep findings in dementia with Lewy bodies. *Mov Disord* **28**, 1416-1423. doi:10.1002/mds.25523

[299] Thaipisuttikul P, Lobach I, Zweig Y, Gurnani A, Galvin JE (2013) Capgras syndrome in Dementia with Lewy Bodies. *Int Psychogeriatr* **25**, 843-849. doi:10.1017/s1041610212002189

[300] Thomas AJ, Burn DJ, Rowan EN, Littlewood E, Newby J, Cousins D, Pakrasi S, Richardson J, Sanders J, McKeith IG (2005) A comparison of the efficacy of donepezil in Parkinson's disease with dementia and dementia with Lewy bodies. *Int J Geriatr Psychiatry* **20**, 938-944. doi:10.1002/gps.1381

[301] Torboli D, Mioni G, Bussé C, Cagnin A, Vallesi A (2023) Subjective experience of time in dementia with Lewy bodies during COVID-19 lockdown. *Curr Psychol* **42**, 4653-4662. doi:10.1007/s12144-021-01811-7

[302] Tsuboi Y, Kochi K, Maruyama H, Matsumoto Y (2022) Zonisamide improves axial symptoms in dementia with Lewy bodies with parkinsonism: Post hoc analysis of clinical trials. *eNeurologicalSci* **26**, 100384. doi:10.1016/j.ensci.2021.100384

[303] Urwyler P, Nef T, Müri R, Archibald N, Makin SM, Collerton D, Taylor JP, Burn D, McKeith I, Mosimann UP (2016) Visual Hallucinations in Eye Disease and Lewy Body Disease. *Am J Geriatr Psychiatry* **24**, 350-358. doi:10.1016/j.jagp.2015.10.007

[304] Van Assche L, Van Aubel E, Van de Ven L, Bouckaert F, Luyten P, Vandenbulcke M (2019) The Neuropsychological Profile and Phenomenology of Late Onset Psychosis: A Cross-sectional Study on the Differential Diagnosis of Very-Late-Onset Schizophrenia-Like Psychosis, Dementia with Lewy Bodies and Alzheimer's Type Dementia with Psychosis. *Arch Clin Neuropsychol* **34**, 183-199. doi:10.1093/arclin/acy034

[305] van de Beek M, van Steenoven I, van der Zande JJ, Porcelijn I, Barkhof F, Stam CJ, Raijmakers P, Scheltens P, Teunissen CE, van der Flier WM, Lemstra AW (2021) Characterization of symptoms and determinants of disease burden in dementia with Lewy bodies: DEvELOP design and baseline results. *Alzheimers Res Ther* **13**, 53. doi:10.1186/s13195-021-00792-w

[306] van den Berg NS, Reesink FE, de Haan EHF, Kremer HPH, Spikman JM, Huitema RB (2021) Emotion Recognition and Traffic-Related Risk-Taking Behavior in Patients with Neurodegenerative Diseases. *J Int Neuropsychol Soc* **27**, 136-145. doi:10.1017/s1355617720000740

[307] Vatter S, McDonald KR, Stanmore E, Clare L, Leroi I (2018) Multidimensional Care Burden in Parkinson-Related Dementia. *J Geriatr Psychiatry Neurol* **31**, 319-328. doi:10.1177/0891988718802104

[308] Vöglein J, Kostova I, Arzberger T, Roeber S, Schmitz P, Simons M, Ruf V, Windl O, Herms J, Dieterich M, Danek A, Höglinger GU, Giese A, Levin J (2021) First symptom

guides diagnosis and prognosis in neurodegenerative diseases-a retrospective study of autopsy proven cases. *Eur J Neurol* **28**, 1801-1811. doi:10.1111/ene.14800

[309] Wajman JR, Cecchini MA, Bertolucci PHF, Mansur LL (2019) Quanti-qualitative components of the semantic verbal fluency test in cognitively healthy controls, mild cognitive impairment, and dementia subtypes. *Appl Neuropsychol Adult* **26**, 533-542. doi:10.1080/23279095.2018.1465426

[310] Watanabe H, Uchiyama M, Yokoi K, Mamiya Y, Narita W, Iizuka O, Baba T, Suzuki K, Mori E, Nishio Y (2023) Behavioral and neural correlates of pareidolic illusions in dementia with Lewy bodies. *Parkinsonism Relat Disord* **113**, 105513. doi:10.1016/j.parkreldis.2023.105513

[311] Webber TA, Kiselica AM, Mikula C, Woods SP (2022) Dispersion-Based Cognitive Intra-Individual Variability in Dementia With Lewy Bodies. *Neuropsychology* **36**, 719-729. doi:10.1037/neu0000856

[312] Weiner MF, Risser RC, Cullum CM, Honig L, White C, 3rd, Speciale S, Rosenberg RN (1996) Alzheimer's disease and its Lewy body variant: a clinical analysis of postmortem verified cases. *Am J Psychiatry* **153**, 1269-1273. doi:10.1176/ajp.153.10.1269

[313] Wu YT, Clare L, Hindle JV, Nelis SM, Martyr A, Matthews FE (2018) Dementia subtype and living well: results from the Improving the experience of Dementia and Enhancing Active Life (IDEAL) study. *BMC Med* **16**, 140. doi:10.1186/s12916-018-1135-2

[314] Yokoi K, Nishio Y, Uchiyama M, Shimomura T, Iizuka O, Mori E (2014) Hallucinators find meaning in noises: pareidolic illusions in dementia with Lewy bodies. *Neuropsychologia* **56**, 245-254. doi:10.1016/j.neuropsychologia.2014.01.017

[315] Yuuki S, Hashimoto M, Koyama A, Matsushita M, Ishikawa T, Fukuhara R, Honda K, Miyagawa Y, Ikeda M, Takebayashi M (2023) Comparison of caregiver burden between dementia with Lewy bodies and Alzheimer's disease. *Psychogeriatrics* **23**, 682-689. doi:10.1111/psyg.12978

[316] Zarkali A, Adams RA, Psarras S, Leyland LA, Rees G, Weil RS (2019) Increased weighting on prior knowledge in Lewy body-associated visual hallucinations. *Brain Commun* **1**, fcz007. doi:10.1093/braincomms/fcz007

[317] Zaslavsky O, Kaneshiro J, Chu F, Teng A, Domoto-Reilly K, Chen AT (2022) Virtual Intervention for Caregivers of Persons With Lewy Body Dementia: Pilot Quasi-Experimental Single-Arm Study. *JMIR Form Res* **6**, e37108. doi:10.2196/37108

[318] Alosco ML, Mez J, Tripodis Y, Kiernan PT, Abdolmohammadi B, Murphy L, Kowall NW, Stein TD, Huber BR, Goldstein LE, Cantu RC, Katz DI, Chaisson CE, Martin B, Solomon TM, McClean MD, Daneshvar DH, Nowinski CJ, Stern RA, McKee AC (2018) Age of first exposure to tackle football and chronic traumatic encephalopathy. *Ann Neurol* **83**, 886-901. doi:10.1002/ana.25245

[319] Asada T (2012) [Prevalence of dementia in Japan: past, present and future]. *Rinsho Shinkeigaku* **52**, 962-964. doi:10.5692/clinicalneurol.52.962

[320] Chaudhuri KR, Hu MT, Brooks DJ (2000) Atypical parkinsonism in Afro-Caribbean and Indian origin immigrants to the UK. *Mov Disord* **15**, 18-23. doi:10.1002/1531-8257(200001)15:1<18::aid-mds1005>3.0.co;2-z

[321] Collía A, Iranzo A, Serradell M, Muñoz-Lopetegi A, Mayà G, Santamaría J, Sánchez-Valle R, Gaig C (2023) Former participation in professional football as an occupation in patients with isolated REM sleep behavior disorder leading to a synucleinopathy: a case-control study. *J Neurol* **270**, 3234-3242. doi:10.1007/s00415-023-11591-8

[322] Croisile B, Tedesco A, Bernard E, Gavant S, Minssieux-Catrix G, Mollion H (2012) [Diagnostic profile of young-onset dementia before 65 years. Experience of a French Memory Referral Center]. *Rev Neurol (Paris)* **168**, 161-169. doi:10.1016/j.neurol.2011.09.003

[323] de Silva HA, Gunatilake SB, Smith AD (2003) Prevalence of dementia in a semi-urban population in Sri Lanka: report from a regional survey. *Int J Geriatr Psychiatry* **18**, 711-715. doi:10.1002/gps.909

[324] Guo X, Meng Q, Zuo M, Su Z, Gan J, Li XD, Zhu H, Gang B, Niu J, Liu S, Ji Y (2023) Costs of dementia with lewy bodies: A Chinese multicenter cross-sectional study. *Int J Geriatr Psychiatry* **38**, e5848. doi:10.1002/gps.5848

[325] Jhoo JH, Kim KW, Huh Y, Lee SB, Park JH, Lee JJ, Choi EA, Han C, Choo IH, Youn JC, Lee DY, Woo JI (2008) Prevalence of dementia and its subtypes in an elderly urban korean population: results from the Korean Longitudinal Study on Health And Aging (KLoSHA). *Dement Geriatr Cogn Disord* **26**, 270-276. doi:10.1159/000160960

[326] Kovacs GG, Alafuzoff I, Al-Sarraj S, Arzberger T, Bogdanovic N, Capellari S, Ferrer I, Gelpi E, Kövari V, Kretzschmar H, Nagy Z, Parchi P, Seilhean D, Soininen H, Troakes C, Budka H (2008) Mixed brain pathologies in dementia: the BrainNet Europe consortium experience. *Dement Geriatr Cogn Disord* **26**, 343-350. doi:10.1159/000161560

[327] Leech RW, Brumback RA, Poduslo SE, Schiffer R, Adesina A (2001) Dementia: the University of Oklahoma autopsy experience. *J Okla State Med Assoc* **94**, 507-511.

[328] López-Pousa S, Garre-Olmo J, Turon-Estrada A, Gelada-Batle E, Lozano-Gallego M, Hernández-Ferrándiz M, Morante-Muñoz V, Peralta-Rodríguez J, Cruz-Reina MM (2003) [Clinical incidence of dementia with Lewy bodies]. *Rev Neurol* **36**, 715-720.

[329] Nwabuobi L, Barbosa W, Sweeney M, Oyler S, Meisel T, Di Rocco A, Chodosh J, Fleisher JE (2019) Sex-related differences in homebound advanced Parkinson's disease patients. *Clin Interv Aging* **14**, 1371-1377. doi:10.2147/cia.S203690

[330] Rahkonen T, Eloniemi-Sulkava U, Rissanen S, Vatanen A, Viramo P, Sulkava R (2003) Dementia with Lewy bodies according to the consensus criteria in a general population aged 75 years or older. *J Neurol Neurosurg Psychiatry* **74**, 720-724. doi:10.1136/jnnp.74.6.720

[331] Santangelo A, Testai M, Albani S, Mamazza G, Pavano S, Zuccaro C, Atteritano M, Berretta M, Tomarchio M, Maugeri D (2010) The clinical and rehabilitative complexity in dementia with Lewy bodies (DLB): experience on a random sample of elderly patients dwelling in an RSA ("Residenza Sanitaria Assistita") of Catania. *Arch Gerontol Geriatr* **51**, e7-10. doi:10.1016/j.archger.2009.06.008

[332] Stevens T, Livingston G, Kitchen G, Manela M, Walker Z, Katona C (2002) Islington study of dementia subtypes in the community. *Br J Psychiatry* **180**, 270-276. doi:10.1192/bjp.180.3.270

[333] van Loenhoud AC, de Boer C, Wols K, Pijnenburg YA, Lemstra AW, Bouwman FH, Prins ND, Scheltens P, Ossenkoppele R, van der Flier WM (2019) High occurrence of transportation and logistics occupations among vascular dementia patients: an observational study. *Alzheimers Res Ther* **11**, 112. doi:10.1186/s13195-019-0570-4
[334] Vieregge P, Hagenah J, Heberlein I, Klein C, Ludin HP (1999) Parkinson's disease in twins: a follow-up study. *Neurology* **53**, 566-572. doi:10.1212/wnl.53.3.566
[335] Brockmann K, Schulte C, Deuschle C, Hauser AK, Heger T, Gasser T, Maetzler W, Berg D (2015) Neurodegenerative CSF markers in genetic and sporadic PD: Classification and prediction in a longitudinal study. *Parkinsonism Relat Disord* **21**, 1427-1434. doi:10.1016/j.parkreldis.2015.10.008
[336] Carli G, Caminiti SP, Galbiati A, Marelli S, Casoni F, Padovani A, Ferini-Strambi L, Perani D (2020) In-vivo signatures of neurodegeneration in isolated rapid eye movement sleep behaviour disorder. *Eur J Neurol* **27**, 1285-1295. doi:10.1111/ene.14215
[337] Chatterjee M, van Steenoven I, Huisman E, Oosterveld L, Berendse H, van der Flier WM, Del Campo M, Lemstra AW, van de Berg WDJ, Teunissen CE (2020) Contactin-1 Is Reduced in Cerebrospinal Fluid of Parkinson's Disease Patients and Is Present within Lewy Bodies. *Biomolecules* **10**. doi:10.3390/biom10081177
[338] Hernandez SM, Tikhonova EB, Karamyshev AL (2020) Protein-Protein Interactions in Alpha-Synuclein Biogenesis: New Potential Targets in Parkinson's Disease. *Front Aging Neurosci* **12**, 72. doi:10.3389/fnagi.2020.00072
[339] Huber M, Beyer L, Prix C, Schönecker S, Palleis C, Rauchmann BS, Morbelli S, Chincarini A, Bruffaerts R, Vandenberghe R, Van Laere K, Kramberger MG, Trost M, Grmek M, Garibotto V, Nicastro N, Frisoni GB, Lemstra AW, van der Zande J, Pilotto A, Padovani A, Garcia-Ptacek S, Savitcheva I, Ochoa-Figueroa MA, Davidsson A, Camacho V, Peira E, Arnaldi D, Bauckneht M, Pardini M, Sambuceti G, Vöglein J, Schnabel J, Unterrainer M, Perneczky R, Pogarell O, Buerger K, Catak C, Bartenstein P, Cumming P, Ewers M, Danek A, Levin J, Aarsland D, Nobili F, Rominger A, Brendel M (2020) Metabolic Correlates of Dopaminergic Loss in Dementia with Lewy Bodies. *Mov Disord* **35**, 595-605. doi:10.1002/mds.27945
[340] Kurzawa-Akanbi M, Hanson PS, Blain PG, Lett DJ, McKeith IG, Chinnery PF, Morris CM (2012) Glucocerebrosidase mutations alter the endoplasmic reticulum and lysosomes in Lewy body disease. *J Neurochem* **123**, 298-309. doi:10.1111/j.1471-4159.2012.07879.x
[341] Majumder S, Srivastava M, Alam P, Saha S, Kumari R, Chand AK, Asthana S, Sen S, Maiti TK (2024) Hotspot site microenvironment in the deubiquitinase OTUB1 drives its stability and aggregation. *J Biol Chem* **300**, 107315. doi:10.1016/j.jbc.2024.107315
[342] Oh Y (2019) Patient-specific pluripotent stem cell-based Parkinson's disease models showing endogenous alpha-synuclein aggregation. *BMB Rep* **52**, 349-359. doi:10.5483/BMBRep.2019.52.6.142
[343] Ouzounoglou E, Kalamatianos D, Emmanouilidou E, Xilouri M, Stefanis L, Vekrellis K, Manolakos ES (2014) In silico modeling of the effects of alpha-synuclein

oligomerization on dopaminergic neuronal homeostasis. *BMC Syst Biol* **8**, 54. doi:10.1186/1752-0509-8-54
[344] Sengupta U, Puangmalai N, Bhatt N, Garcia S, Zhao Y, Kayed R (2020) Polymorphic α-Synuclein Strains Modified by Dopamine and Docosahexaenoic Acid Interact Differentially with Tau Protein. *Mol Neurobiol* **57**, 2741-2765. doi:10.1007/s12035-020-01913-6
[345] Sinclair L, Brenton J, Liu AKL, MacLachlan R, Gentleman SM, Love S (2022) Possible Contribution of Altered Cholinergic Activity in the Visual Cortex in Visual Hallucinations in Parkinson's Disease. *J Neuropsychiatry Clin Neurosci* **34**, 168-176. doi:10.1176/appi.neuropsych.21040103
[346] Teraoka M, Nakaso K, Kusumoto C, Katano S, Tajima N, Yamashita A, Zushi T, Ito S, Matsura T (2012) Cytoprotective effect of chlorogenic acid against α-synuclein-related toxicity in catecholaminergic PC12 cells. *J Clin Biochem Nutr* **51**, 122-127. doi:10.3164/jcbn.D-11-00030
[347] Valcic M, Khoury MA, Kim J, Fornazzari L, Churchill NW, Ismail Z, De Luca V, Tsuang D, Schweizer TA, Munoz DG, Fischer CE (2022) Determining Whether Sex and Zygosity Modulates the Association between APOE4 and Psychosis in a Neuropathologically-Confirmed Alzheimer's Disease Cohort. *Brain Sci* **12**. doi:10.3390/brainsci12091266
[348] Xu S, Liu Y, Wang Q, Liu F, Xian Y, Xu F, Liu Y (2023) Gut microbiota in combination with blood metabolites reveals characteristics of the disease cluster of coronary artery disease and cognitive impairment: a Mendelian randomization study. *Front Immunol* **14**, 1308002. doi:10.3389/fimmu.2023.1308002
[349] Yang B, Yang Z, Liu H, Qi H (2023) Dynamic modelling and tristability analysis of misfolded α-synuclein degraded via autophagy in Parkinson's disease. *Biosystems* **233**, 105036. doi:10.1016/j.biosystems.2023.105036
[350] Ardley HC, Scott GB, Rose SA, Tan NG, Markham AF, Robinson PA (2003) Inhibition of proteasomal activity causes inclusion formation in neuronal and non-neuronal cells overexpressing Parkin. *Mol Biol Cell* **14**, 4541-4556. doi:10.1091/mbc.e03-02-0078
[351] Au R, Seshadri S, Knox K, Beiser A, Himali JJ, Cabral HJ, Auerbach S, Green RC, Wolf PA, McKee AC (2012) The Framingham Brain Donation Program: neuropathology along the cognitive continuum. *Curr Alzheimer Res* **9**, 673-686. doi:10.2174/156720512801322609
[352] Basu S, Song M, Adams L, Jeong I, Je G, Guhathakurta S, Jiang J, Boparai N, Dai W, Cardozo-Pelaez F, Tatulian SA, Han KY, Elliott J, Baum J, McLean PJ, Dickson DW, Kim YS (2023) Transcriptional mutagenesis of α-synuclein caused by DNA oxidation in Parkinson's disease pathogenesis. *Acta Neuropathol* **146**, 685-705. doi:10.1007/s00401-023-02632-7
[353] Beekman AT (2011) Neuropathological correlates of late-life depression. *Expert Rev Neurother* **11**, 947-949. doi:10.1586/ern.11.88
[354] Bertrand E, Lechowicz W, Szpak GM, Dymecki J (1997) Qualitative and quantitative analysis of locus coeruleus neurons in Parkinson's disease. *Folia Neuropathol* **35**, 80-86.

[355] Bétemps D, Verchère J, Brot S, Morignat E, Bousset L, Gaillard D, Lakhdar L, Melki R, Baron T (2014) Alpha-synuclein spreading in M83 mice brain revealed by detection of pathological α-synuclein by enhanced ELISA. *Acta Neuropathol Commun* **2**, 29. doi:10.1186/2051-5960-2-29

[356] Bhak G, Lee JH, Hahn JS, Paik SR (2009) Granular assembly of alpha-synuclein leading to the accelerated amyloid fibril formation with shear stress. *PLoS One* **4**, e4177. doi:10.1371/journal.pone.0004177

[357] Brayne C, Richardson K, Matthews FE, Fleming J, Hunter S, Xuereb JH, Paykel E, Mukaetova-Ladinska EB, Huppert FA, O'Sullivan A, Dening T (2009) Neuropathological correlates of dementia in over-80-year-old brain donors from the population-based Cambridge city over-75s cohort (CC75C) study. *J Alzheimers Dis* **18**, 645-658. doi:10.3233/jad-2009-1182

[358] Bukhatwa S, Zeng BY, Rose S, Jenner P (2010) A comparison of changes in proteasomal subunit expression in the substantia nigra in Parkinson's disease, multiple system atrophy and progressive supranuclear palsy. *Brain Res* **1326**, 174-183. doi:10.1016/j.brainres.2010.02.045

[359] Chan-Palay V, Asan E (1989) Alterations in catecholamine neurons of the locus coeruleus in senile dementia of the Alzheimer type and in Parkinson's disease with and without dementia and depression. *J Comp Neurol* **287**, 373-392. doi:10.1002/cne.902870308

[360] Chanthamontri C, Liu J, McLuckey SA (2009) Charge State Dependent Fragmentation of Gaseous α-Synuclein Cations via Ion Trap and Beam-Type Collisional Activation. *Int J Mass Spectrom* **283**, 9-16. doi:10.1016/j.ijms.2008.12.007

[361] Chatterjee D, D'Brant LY, Hiller BM, Marmion DJ, Sandoval IM, Luk KC, Manfredsson FP, Messer A, Kordower JH, Butler DC (2024) ENGINEERED NANOBODIES WITH PROGRAMMABLE TARGET ANTIGEN PROTEOLYSIS (PTAP) FUSIONS REGULATE INTRACELLULAR ALPHA-SYNUCLEIN IN VITRO AND IN VIVO. *Res Sq*. doi:10.21203/rs.3.rs-4088206/v1

[362] Chew KC, Ang ET, Tai YK, Tsang F, Lo SQ, Ong E, Ong WY, Shen HM, Lim KL, Dawson VL, Dawson TM, Soong TW (2011) Enhanced autophagy from chronic toxicity of iron and mutant A53T α-synuclein: implications for neuronal cell death in Parkinson disease. *J Biol Chem* **286**, 33380-33389. doi:10.1074/jbc.M111.268409

[363] Court JA, Ballard CG, Piggott MA, Johnson M, O'Brien JT, Holmes C, Cairns N, Lantos P, Perry RH, Jaros E, Perry EK (2001) Visual hallucinations are associated with lower alpha bungarotoxin binding in dementia with Lewy bodies. *Pharmacol Biochem Behav* **70**, 571-579. doi:10.1016/s0091-3057(01)00644-x

[364] D'Antonio F, Boccia M, Di Vita A, Suppa A, Fabbrini A, Canevelli M, Caramia F, Fiorelli M, Guariglia C, Ferracuti S, de Lena C, Aarsland D, Ffytche D (2022) Visual hallucinations in Lewy body disease: pathophysiological insights from phenomenology. *J Neurol* **269**, 3636-3652. doi:10.1007/s00415-022-10983-6

[365] Danics K, Visanji NP, Ichimata S, Mathur S, Sára-Klausz G, Kovacs GG (2023) Prevalence and Distribution of Lewy Pathology in a Homeless Population. *Can J Neurol Sci*, 1-7. doi:10.1017/cjn.2023.291

[366] Drolet RE, Cannon JR, Montero L, Greenamyre JT (2009) Chronic rotenone exposure reproduces Parkinson's disease gastrointestinal neuropathology. *Neurobiol Dis* **36**, 96-102. doi:10.1016/j.nbd.2009.06.017
[367] Dymecki J, Lechowicz W, Bertrand E, Szpak GM (1996) Changes in dopaminergic neurons of the mesocorticolimbic system in Parkinson's disease. *Folia Neuropathol* **34**, 102-106.
[368] Erskine D, Thomas AJ, Attems J, Taylor JP, McKeith IG, Morris CM, Khundakar AA (2017) Specific patterns of neuronal loss in the pulvinar nucleus in dementia with lewy bodies. *Mov Disord* **32**, 414-422. doi:10.1002/mds.26887
[369] Erskine D, Thomas AJ, Taylor JP, Savage MA, Attems J, McKeith IG, Morris CM, Khundakar AA (2017) Neuronal Loss and Α-Synuclein Pathology in the Superior Colliculus and Its Relationship to Visual Hallucinations in Dementia with Lewy Bodies. *Am J Geriatr Psychiatry* **25**, 595-604. doi:10.1016/j.jagp.2017.01.005
[370] Foley P, Riederer P (2000) Influence of neurotoxins and oxidative stress on the onset and progression of Parkinson's disease. *J Neurol* **247 Suppl 2**, Ii82-94. doi:10.1007/pl00007766
[371] Haglund M, Friberg N, Danielsson EJ, Norrman J, Englund E (2016) A methodological study of locus coeruleus degeneration in dementing disorders. *Clin Neuropathol* **35**, 287-294. doi:10.5414/np300930
[372] Hansen C, Angot E, Bergström AL, Steiner JA, Pieri L, Paul G, Outeiro TF, Melki R, Kallunki P, Fog K, Li JY, Brundin P (2011) α-Synuclein propagates from mouse brain to grafted dopaminergic neurons and seeds aggregation in cultured human cells. *J Clin Invest* **121**, 715-725. doi:10.1172/jci43366
[373] Hatsuta H, Takao M, Nakano Y, Nogami A, Uchino A, Sumikura H, Kanemaru K, Arai T, Itoh Y, Murayama S (2016) Reduction of Small Fibers of Thoracic Ventral Roots and Neurons of Intermediolateral Nucleus in Parkinson Disease and Dementia with Lewy Bodies. *J Parkinsons Dis* **6**, 325-334. doi:10.3233/jpd-150773
[374] Hirsch EC, Graybiel AM, Agid Y (1989) Selective vulnerability of pigmented dopaminergic neurons in Parkinson's disease. *Acta Neurol Scand Suppl* **126**, 19-22. doi:10.1111/j.1600-0404.1989.tb01778.x
[375] Hochstrasser H, Bauer P, Walter U, Behnke S, Spiegel J, Csoti I, Zeiler B, Bornemann A, Pahnke J, Becker G, Riess O, Berg D (2004) Ceruloplasmin gene variations and substantia nigra hyperechogenicity in Parkinson disease. *Neurology* **63**, 1912-1917. doi:10.1212/01.wnl.0000144276.29988.c3
[376] Hofer A, Berg D, Asmus F, Niwar M, Ransmayr G, Riemenschneider M, Bonelli SB, Steffelbauer M, Ceballos-Baumann A, Haussermann P, Behnke S, Krüger R, Prestel J, Sharma M, Zimprich A, Riess O, Gasser T (2005) The role of alpha-synuclein gene multiplications in early-onset Parkinson's disease and dementia with Lewy bodies. *J Neural Transm (Vienna)* **112**, 1249-1254. doi:10.1007/s00702-004-0263-3
[377] Ihara M, Yamasaki N, Hagiwara A, Tanigaki A, Kitano A, Hikawa R, Tomimoto H, Noda M, Takanashi M, Mori H, Hattori N, Miyakawa T, Kinoshita M (2007) Sept4, a component of presynaptic scaffold and Lewy bodies, is required for the suppression of alpha-synuclein neurotoxicity. *Neuron* **53**, 519-533. doi:10.1016/j.neuron.2007.01.019

[378] Imamura K, Hishikawa N, Ono K, Suzuki H, Sawada M, Nagatsu T, Yoshida M, Hashizume Y (2005) Cytokine production of activated microglia and decrease in neurotrophic factors of neurons in the hippocampus of Lewy body disease brains. *Acta Neuropathol* **109**, 141-150. doi:10.1007/s00401-004-0919-y
[379] Jellinger KA, Seppi K, Wenning GK, Poewe W (2002) Impact of coexistent Alzheimer pathology on the natural history of Parkinson's disease. *J Neural Transm (Vienna)* **109**, 329-339. doi:10.1007/s007020200027
[380] Kabiraj P, Marin JE, Varela-Ramirez A, Zubia E, Narayan M (2014) Ellagic acid mitigates SNO-PDI induced aggregation of Parkinsonian biomarkers. *ACS Chem Neurosci* **5**, 1209-1220. doi:10.1021/cn500214k
[381] Kempster PA, Williams DR, Selikhova M, Holton J, Revesz T, Lees AJ (2007) Patterns of levodopa response in Parkinson's disease: a clinico-pathological study. *Brain* **130**, 2123-2128. doi:10.1093/brain/awm142
[382] Khundakar AA, Hanson PS, Erskine D, Lax NZ, Roscamp J, Karyka E, Tsefou E, Singh P, Cockell SJ, Gribben A, Ramsay L, Blain PG, Mosimann UP, Lett DJ, Elstner M, Turnbull DM, Xiang CC, Brownstein MJ, O'Brien JT, Taylor JP, Attems J, Thomas AJ, McKeith IG, Morris CM (2016) Analysis of primary visual cortex in dementia with Lewy bodies indicates GABAergic involvement associated with recurrent complex visual hallucinations. *Acta Neuropathol Commun* **4**, 66. doi:10.1186/s40478-016-0334-3
[383] Lee EN, Lee SY, Lee D, Kim J, Paik SR (2003) Lipid interaction of alpha-synuclein during the metal-catalyzed oxidation in the presence of Cu2+ and H2O2. *J Neurochem* **84**, 1128-1142. doi:10.1046/j.1471-4159.2003.01612.x
[384] Lengacher NA, Tomlinson JJ, Jochum AK, Franz J, Hasan Ali O, Flatz L, Jochum W, Penninger J, Stadelmann C, Woulfe JM, Schlossmacher MG (2024) Neuropathological assessment of the olfactory bulb and tract in individuals with COVID-19. *Acta Neuropathol Commun* **12**, 70. doi:10.1186/s40478-024-01761-8
[385] Li JY, Englund E, Holton JL, Soulet D, Hagell P, Lees AJ, Lashley T, Quinn NP, Rehncrona S, Björklund A, Widner H, Revesz T, Lindvall O, Brundin P (2008) Lewy bodies in grafted neurons in subjects with Parkinson's disease suggest host-to-graft disease propagation. *Nat Med* **14**, 501-503. doi:10.1038/nm1746
[386] Libow LS, Frisina PG, Haroutunian V, Perl DP, Purohit DP (2009) Parkinson's disease dementia: a diminished role for the Lewy body. *Parkinsonism Relat Disord* **15**, 572-575. doi:10.1016/j.parkreldis.2009.02.003
[387] Martin ZS, Neugebauer V, Dineley KT, Kayed R, Zhang W, Reese LC, Taglialatela G (2012) α-Synuclein oligomers oppose long-term potentiation and impair memory through a calcineurin-dependent mechanism: relevance to human synucleopathic diseases. *J Neurochem* **120**, 440-452. doi:10.1111/j.1471-4159.2011.07576.x
[388] Martinez-Valbuena I, Amat-Villegas I, Valenti-Azcarate R, Carmona-Abellan MDM, Marcilla I, Tuñon MT, Luquin MR (2018) Interaction of amyloidogenic proteins in pancreatic β cells from subjects with synucleinopathies. *Acta Neuropathol* **135**, 877-886. doi:10.1007/s00401-018-1832-0

[389] McAleese KE, Walker L, Erskine D, Thomas AJ, McKeith IG, Attems J (2017) TDP-43 pathology in Alzheimer's disease, dementia with Lewy bodies and ageing. *Brain Pathol* **27**, 472-479. doi:10.1111/bpa.12424

[390] McNaught KS, Shashidharan P, Perl DP, Jenner P, Olanow CW (2002) Aggresome-related biogenesis of Lewy bodies. *Eur J Neurosci* **16**, 2136-2148. doi:10.1046/j.1460-9568.2002.02301.x

[391] Miki Y, Tanji K, Mori F, Utsumi J, Sasaki H, Kakita A, Takahashi H, Wakabayashi K (2016) Alteration of Upstream Autophagy-Related Proteins (ULK1, ULK2, Beclin1, VPS34 and AMBRA1) in Lewy Body Disease. *Brain Pathol* **26**, 359-370. doi:10.1111/bpa.12297

[392] Nelson PT, Schmitt FA, Jicha GA, Kryscio RJ, Abner EL, Smith CD, Van Eldik LJ, Markesbery WR (2010) Association between male gender and cortical Lewy body pathology in large autopsy series. *J Neurol* **257**, 1875-1881. doi:10.1007/s00415-010-5630-4

[393] Nemes Z, Devreese B, Steinert PM, Van Beeumen J, Fésüs L (2004) Cross-linking of ubiquitin, HSP27, parkin, and alpha-synuclein by gamma-glutamyl-epsilon-lysine bonds in Alzheimer's neurofibrillary tangles. *Faseb j* **18**, 1135-1137. doi:10.1096/fj.04-1493fje

[394] Oh CK, Dolatabadi N, Cieplak P, Diaz-Meco MT, Moscat J, Nolan JP, Nakamura T, Lipton SA (2022) S-Nitrosylation of p62 Inhibits Autophagic Flux to Promote α-Synuclein Secretion and Spread in Parkinson's Disease and Lewy Body Dementia. *J Neurosci* **42**, 3011-3024. doi:10.1523/jneurosci.1508-21.2022

[395] Paik SR, Shin HJ, Lee JH (2000) Metal-catalyzed oxidation of alpha-synuclein in the presence of Copper(II) and hydrogen peroxide. *Arch Biochem Biophys* **378**, 269-277. doi:10.1006/abbi.2000.1822

[396] Pal R, Miranda M, Narayan M (2011) Nitrosative stress-induced Parkinsonian Lewy-like aggregates prevented through polyphenolic phytochemical analog intervention. *Biochem Biophys Res Commun* **404**, 324-329. doi:10.1016/j.bbrc.2010.11.117

[397] Perry EK, Marshall E, Thompson P, McKeith IG, Collerton D, Fairbairn AF, Ferrier IN, Irving D, Perry RH (1993) Monoaminergic activities in Lewy body dementia: relation to hallucinosis and extrapyramidal features. *J Neural Transm Park Dis Dement Sect* **6**, 167-177. doi:10.1007/bf02260919

[398] Piscopo P, Marcon G, Piras MR, Crestini A, Campeggi LM, Deiana E, Cherchi R, Tanda F, Deplano A, Vanacore N, Tagliavini F, Pocchiari M, Giaccone G, Confaloni A (2008) A novel PSEN2 mutation associated with a peculiar phenotype. *Neurology* **70**, 1549-1554. doi:10.1212/01.wnl.0000310643.53587.87

[399] Popescu A, Lippa CF, Lee VM, Trojanowski JQ (2004) Lewy bodies in the amygdala: increase of alpha-synuclein aggregates in neurodegenerative diseases with tau-based inclusions. *Arch Neurol* **61**, 1915-1919. doi:10.1001/archneur.61.12.1915

[400] Priemer DS, Folkerth RD (2021) Dementia in the Forensic Setting: Diagnoses Obtained Using a Condensed Protocol at the Office of Chief Medical Examiner, New York City. *J Neuropathol Exp Neurol* **80**, 724-730. doi:10.1093/jnen/nlab059

[401] Pullen AH, Martin JE (1995) Ultrastructural abnormalities with inclusions in Onuf's nucleus in motor neuron disease (amyotrophic lateral sclerosis). *Neuropathol Appl Neurobiol* **21**, 327-340. doi:10.1111/j.1365-2990.1995.tb01067.x
[402] Rajput AH, Rozdilsky B, Rajput A (1991) Accuracy of clinical diagnosis in parkinsonism--a prospective study. *Can J Neurol Sci* **18**, 275-278. doi:10.1017/s0317167100031814
[403] Reynolds AD, Glanzer JG, Kadiu I, Ricardo-Dukelow M, Chaudhuri A, Ciborowski P, Cerny R, Gelman B, Thomas MP, Mosley RL, Gendelman HE (2008) Nitrated alpha-synuclein-activated microglial profiling for Parkinson's disease. *J Neurochem* **104**, 1504-1525. doi:10.1111/j.1471-4159.2007.05087.x
[404] Reynolds NP, Soragni A, Rabe M, Verdes D, Liverani E, Handschin S, Riek R, Seeger S (2011) Mechanism of membrane interaction and disruption by α-synuclein. *J Am Chem Soc* **133**, 19366-19375. doi:10.1021/ja2029848
[405] Roodveldt C, Bertoncini CW, Andersson A, van der Goot AT, Hsu ST, Fernández-Montesinos R, de Jong J, van Ham TJ, Nollen EA, Pozo D, Christodoulou J, Dobson CM (2009) Chaperone proteostasis in Parkinson's disease: stabilization of the Hsp70/alpha-synuclein complex by Hip. *Embo j* **28**, 3758-3770. doi:10.1038/emboj.2009.298
[406] Roodveldt C, Labrador-Garrido A, Gonzalez-Rey E, Lachaud CC, Guilliams T, Fernandez-Montesinos R, Benitez-Rondan A, Robledo G, Hmadcha A, Delgado M, Dobson CM, Pozo D (2013) Preconditioning of microglia by α-synuclein strongly affects the response induced by toll-like receptor (TLR) stimulation. *PLoS One* **8**, e79160. doi:10.1371/journal.pone.0079160
[407] Schrempf W, Katona I, Dogan I, Felbert VV, Wienecke M, Heller J, Maier A, Hermann A, Linse K, Brandt MD, Reichmann H, Schulz JB, Schiefer J, Oertel WH, Storch A, Weis J, Reetz K (2016) Reduced intraepidermal nerve fiber density in patients with REM sleep behavior disorder. *Parkinsonism Relat Disord* **29**, 10-16. doi:10.1016/j.parkreldis.2016.06.003
[408] Sinclair LI, Kumar A, Darreh-Shori T, Love S (2019) Visual hallucinations in Alzheimer's disease do not seem to be associated with chronic hypoperfusion of to visual processing areas V2 and V3 but may be associated with reduced cholinergic input to these areas. *Alzheimers Res Ther* **11**, 80. doi:10.1186/s13195-019-0519-7
[409] Snowden JS, Thompson JC, Stopford CL, Richardson AM, Gerhard A, Neary D, Mann DM (2011) The clinical diagnosis of early-onset dementias: diagnostic accuracy and clinicopathological relationships. *Brain* **134**, 2478-2492. doi:10.1093/brain/awr189
[410] Tanaka MT, Miki Y, Bettencourt C, Ozaki T, Tanji K, Mori F, Kakita A, Wakabayashi K (2022) Involvement of autophagic protein DEF8 in Lewy bodies. *Biochem Biophys Res Commun* **623**, 170-175. doi:10.1016/j.bbrc.2022.07.069
[411] Tanji K, Toki T, Tamo W, Imaizumi T, Matsumiya T, Mori F, Takahashi H, Satoh K, Wakabayashi K (2003) Glycogen synthase kinase-3beta phosphorylates synphilin-1 in vitro. *Neuropathology* **23**, 199-202. doi:10.1046/j.1440-1789.2003.00503.x
[412] Tsopelas C, Stewart R, Savva GM, Brayne C, Ince P, Thomas A, Matthews FE (2011) Neuropathological correlates of late-life depression in older people. *Br J Psychiatry* **198**, 109-114. doi:10.1192/bjp.bp.110.078816

[413] Tsuboi Y, Josephs KA, Boeve BF, Litvan I, Caselli RJ, Caviness JN, Uitti RJ, Bott AD, Dickson DW (2005) Increased tau burden in the cortices of progressive supranuclear palsy presenting with corticobasal syndrome. *Mov Disord* **20**, 982-988. doi:10.1002/mds.20478

[414] Valensin D, Dell'Acqua S, Kozlowski H, Casella L (2016) Coordination and redox properties of copper interaction with α-synuclein. *J Inorg Biochem* **163**, 292-300. doi:10.1016/j.jinorgbio.2016.04.012

[415] Verasdonck J, Bousset L, Gath J, Melki R, Böckmann A, Meier BH (2016) Further exploration of the conformational space of α-synuclein fibrils: solid-state NMR assignment of a high-pH polymorph. *Biomol NMR Assign* **10**, 5-12. doi:10.1007/s12104-015-9628-9

[416] Vieira SRL, Schapira AHV (2021) Glucocerebrosidase mutations: A paradigm for neurodegeneration pathways. *Free Radic Biol Med* **175**, 42-55. doi:10.1016/j.freeradbiomed.2021.08.230

[417] Wakabayashi K, Takahashi H, Oyanagi K, Ikuta F (1993) [Incidental occurrence of Lewy bodies in the brains of elderly patients--the relevance to aging and Parkinson's disease]. *No To Shinkei* **45**, 1033-1038.

[418] Wakabayashi K, Yoshimoto M, Fukushima T, Koide R, Horikawa Y, Morita T, Takahashi H (1999) Widespread occurrence of alpha-synuclein/NACP-immunoreactive neuronal inclusions in juvenile and adult-onset Hallervorden-Spatz disease with Lewy bodies. *Neuropathol Appl Neurobiol* **25**, 363-368. doi:10.1046/j.1365-2990.1999.00193.x

[419] Yuan YH, Yan WF, Sun JD, Huang JY, Mu Z, Chen NH (2015) The molecular mechanism of rotenone-induced α-synuclein aggregation: emphasizing the role of the calcium/GSK3β pathway. *Toxicol Lett* **233**, 163-171. doi:10.1016/j.toxlet.2014.11.029

[420] Zabrocki P, Bastiaens I, Delay C, Bammens T, Ghillebert R, Pellens K, De Virgilio C, Van Leuven F, Winderickx J (2008) Phosphorylation, lipid raft interaction and traffic of alpha-synuclein in a yeast model for Parkinson. *Biochim Biophys Acta* **1783**, 1767-1780. doi:10.1016/j.bbamcr.2008.06.010

[421] Zhang F, Ji LN, Tang L, Hu J, Hu HY, Xu HJ, He JH (2005) Structural evidence for alpha-synuclein fibrils using in situ atomic force microscopy. *Acta Biochim Biophys Sin (Shanghai)* **37**, 113-118.

[422] Zhang J, Park ES, Park HJ, Yan R, Grudniewska M, Zhang X, Oh S, Yang X, Baum J, Mouradian MM (2020) Apoptosis signal regulating kinase 1 deletion mitigates α-synuclein pre-formed fibril propagation in mice. *Neurobiol Aging* **85**, 49-57. doi:10.1016/j.neurobiolaging.2019.09.012

[423] Zhang Y, Ma H, Xie B, Han C, Wang C, Qing H, Deng Y (2013) Alpha-synuclein overexpression induced mitochondrial damage by the generation of endogenous neurotoxins in PC12 cells. *Neurosci Lett* **547**, 65-69. doi:10.1016/j.neulet.2013.05.012

[424] Zhou S, Meng Q, Li L, Hai L, Wang Z, Li Z, Sun Y (2021) Identification of a Qualitative Signature for the Diagnosis of Dementia With Lewy Bodies. *Front Genet* **12**, 758103. doi:10.3389/fgene.2021.758103

[425] Beretta L, Caminiti SP, Santangelo R, Magnani G, Ferrari-Pellegrini F, Caffarra P, Perani D (2019) Two distinct pathological substrates associated with MMSE-pentagons item deficit in DLB and AD. *Neuropsychologia* **133**, 107174. doi:10.1016/j.neuropsychologia.2019.107174
[426] Booij J, Dubroff J, Pryma D, Yu J, Agarwal R, Lakhani P, Kuo PH (2017) Diagnostic Performance of the Visual Reading of (123)I-Ioflupane SPECT Images With or Without Quantification in Patients With Movement Disorders or Dementia. *J Nucl Med* **58**, 1821-1826. doi:10.2967/jnumed.116.189266
[427] Brockhuis B, Sławek J, Wieczorek D, Ussorowska D, Derejko M, Romanowicz G, Marks W, Dubaniewicz M (2006) Cerebral blood flow changes in patients with dementia with Lewy bodies (DLB). A study of 6 cases. *Nucl Med Rev Cent East Eur* **9**, 114-118.
[428] Burkhard PR, Sanchez JC, Landis T, Hochstrasser DF (2001) CSF detection of the 14-3-3 protein in unselected patients with dementia. *Neurology* **56**, 1528-1533. doi:10.1212/wnl.56.11.1528
[429] Carli G, Meles SK, Reesink FE, de Jong BM, Pilotto A, Padovani A, Galbiati A, Ferini-Strambi L, Leenders KL, Perani D (2023) Comparison of univariate and multivariate analyses for brain [18F]FDG PET data in α-synucleinopathies. *Neuroimage Clin* **39**, 103475. doi:10.1016/j.nicl.2023.103475
[430] D'Antonio F, Teghil A, Boccia M, Bechi Gabrielli G, Giulietti G, Conti D, Suppa A, Fabbrini A, Fiorelli M, Caramia F, Bruno G, Guariglia C, Aarsland D, Ffytche D (2024) Distinct grey and white matter changes are associated with the phenomenology of visual hallucinations in Lewy Body Disease. *Sci Rep* **14**, 14748. doi:10.1038/s41598-024-65536-w
[431] Donnemiller E, Heilmann J, Wenning GK, Berger W, Decristoforo C, Moncayo R, Poewe W, Ransmayr G (1997) Brain perfusion scintigraphy with 99mTc-HMPAO or 99mTc-ECD and 123I-beta-CIT single-photon emission tomography in dementia of the Alzheimer-type and diffuse Lewy body disease. *Eur J Nucl Med* **24**, 320-325. doi:10.1007/bf01728771
[432] Firbank MJ, Collerton D, Morgan KD, Schumacher J, Donaghy PC, O'Brien JT, Thomas A, Taylor JP (2024) Functional connectivity in Lewy body disease with visual hallucinations. *Eur J Neurol* **31**, e16115. doi:10.1111/ene.16115
[433] Haglund M, Heyman I, Javanshiri K (2024) Progressive QTc prolongation and reduced heart rate variability in dementia with Lewy bodies compared to Alzheimer's disease. *Parkinsonism Relat Disord* **122**, 106947. doi:10.1016/j.parkreldis.2024.106947
[434] Hanyu H, Asano T, Sakamoto S, Kogure D, Iwamoto T, Takasaki M (1999) [Is hippocampal atrophy a specific change for Alzheimer's disease?]. *No To Shinkei* **51**, 947-951.
[435] Howard R, David A, Woodruff P, Mellers I, Wright J, Brammer M, Bullmore E, Williams S (1997) Seeing visual hallucinations with functional magnetic resonance imaging. *Dement Geriatr Cogn Disord* **8**, 73-77. doi:10.1159/000106610
[436] Jiménez-Hoyuela García JM, Campos Arillo V, Rebollo Aguirre AC, Gómez Doblas JJ, Gutiérrez Hurtado A (2005) [Early alteration of adrenergic cardiac function in

parkinsonisms with Lewy bodies]. *Rev Esp Med Nucl* **24**, 93-100. doi:10.1157/13071684
[437] Killinger BA, Marshall LL, Chatterjee D, Chu Y, Bras J, Guerreiro R, Kordower JH (2022) In situ proximity labeling identifies Lewy pathology molecular interactions in the human brain. *Proc Natl Acad Sci U S A* **119**. doi:10.1073/pnas.2114405119
[438] Klöppel S, Yang S, Kellner E, Reisert M, Heimbach B, Urbach H, Linn J, Weidauer S, Andres T, Bröse M, Lahr J, Lützen N, Meyer PT, Peter J, Abdulkadir A, Hellwig S, Egger K (2018) Voxel-wise deviations from healthy aging for the detection of region-specific atrophy. *Neuroimage Clin* **20**, 851-860. doi:10.1016/j.nicl.2018.09.013
[439] Labidi J, Warniez A, Derambure P, Lebouvier T, Pasquier F, Delval A, Betrouni N (2024) Qualitative versus quantitative assessment of electroencephalography in cognitive decline: Comparison in a clinical population. *Neurophysiol Clin* **54**, 102995. doi:10.1016/j.neucli.2024.102995
[440] Lee H, Brekelmans GJ, Roks G (2015) The EEG as a diagnostic tool in distinguishing between dementia with Lewy bodies and Alzheimer's disease. *Clin Neurophysiol* **126**, 1735-1739. doi:10.1016/j.clinph.2014.11.021
[441] Luzny J, Ivanova K (2016) DatSCAN In Differential Diagnostics of Lewy Body Disease. *Arch Iran Med* **19**, 449-452.
[442] Minoshima S, Mosci K, Cross D, Thientunyakit T (2021) Brain [F-18]FDG PET for Clinical Dementia Workup: Differential Diagnosis of Alzheimer's Disease and Other Types of Dementing Disorders. *Semin Nucl Med* **51**, 230-240. doi:10.1053/j.semnuclmed.2021.01.002
[443] Mukku SSR, Sivakumar PT, Nagaraj C, Mangalore S, Harbishettar V, Varghese M (2019) Clinical utility of 18F-FDG-PET/MRI brain in dementia: Preliminary experience from a geriatric clinic in South India. *Asian J Psychiatr* **44**, 99-105. doi:10.1016/j.ajp.2019.07.001
[444] Nara S, Fujii H, Tsukada H, Tsuda I (2022) Visual hallucinations in dementia with Lewy bodies originate from necrosis of characteristic neurons and connections in three-module perception model. *Sci Rep* **12**, 14172. doi:10.1038/s41598-022-18313-6
[445] Nardone R, Bratti A, Tezzon F (2006) Motor cortex inhibitory circuits in dementia with Lewy bodies and in Alzheimer's disease. *J Neural Transm (Vienna)* **113**, 1679-1684. doi:10.1007/s00702-006-0551-1
[446] Nosaka H, Onoguchi M, Tsushima H, Suda M, Kurata S, Onoma A, Murakawa R (2022) Influence of brain atrophy using semiquantitative analysis in [(123)I]FP-CIT single-photon emission computed tomography by a Monte Carlo simulation study. *Sci Rep* **12**, 168. doi:10.1038/s41598-021-04078-x
[447] Oliveira FPM, Walker Z, Walker RWH, Attems J, Castanheira JC, Silva Â, Oliveira C, Vaz S, Silva M, Costa DC (2021) (123)I-FP-CIT SPECT in dementia with Lewy bodies, Parkinson's disease and Alzheimer's disease: a new quantitative analysis of autopsy confirmed cases. *J Neurol Neurosurg Psychiatry*. doi:10.1136/jnnp-2020-324606
[448] Philippi N, Noblet V, Hamdaoui M, Soulier D, Botzung A, Ehrhard E, Cretin B, Blanc F (2020) The insula, a grey matter of tastes: a volumetric MRI study in dementia with Lewy bodies. *Alzheimers Res Ther* **12**, 79. doi:10.1186/s13195-020-00645-y

[449] Santra A, Sinha GK, Neogi R, Thukral RK (2014) (99m)Tc-hexamethyl propyleneamine oxime brain perfusion single photon emission computed tomography in characterization of dementia: an initial experience in Indian clinical practice. *World J Nucl Med* **13**, 120-127. doi:10.4103/1450-1147.139143
[450] Sinai A, Nassar M, Shornikov L, Constantinescu M, Zaaroor M, Schlesinger I (2024) Focused Ultrasound Thalamotomy for Tremor Relief in Atypical Parkisnsonism. *Parkinsons Dis* **2024**, 6643510. doi:10.1155/2024/6643510
[451] Suzuki K, Seth AK, Schwartzman DJ (2023) Modelling phenomenological differences in aetiologically distinct visual hallucinations using deep neural networks. *Front Hum Neurosci* **17**, 1159821. doi:10.3389/fnhum.2023.1159821
[452] Tateno M, Kobayashi S, Shirasaka T, Furukawa Y, Fujii K, Morii H, Yasumura S, Utsumi K, Saito T (2008) Comparison of the usefulness of brain perfusion SPECT and MIBG myocardial scintigraphy for the diagnosis of dementia with Lewy bodies. *Dement Geriatr Cogn Disord* **26**, 453-457. doi:10.1159/000165918
[453] Akbar U, Friedman JH (2022) Long-term outcomes with pimavanserin for psychosis in clinical practice. *Clin Park Relat Disord* **6**, 100143. doi:10.1016/j.prdoa.2022.100143
[454] Boeve BF, Silber MH, Ferman TJ (2003) Melatonin for treatment of REM sleep behavior disorder in neurologic disorders: results in 14 patients. *Sleep Med* **4**, 281-284. doi:10.1016/s1389-9457(03)00072-8
[455] Dale MC, Libretto SE, Patterson C, Anderson J, Choudhury T, McCafferty F, McWilliam C, Richardson M (2003) Clinical experience of galantamine in dementia: a series of case reports. *Curr Med Res Opin* **19**, 508-518. doi:10.1185/030079903125002054
[456] de Rus Jacquet A, Timmers M, Ma SY, Thieme A, McCabe GP, Vest JHC, Lila MA, Rochet JC (2017) Lumbee traditional medicine: Neuroprotective activities of medicinal plants used to treat Parkinson's disease-related symptoms. *J Ethnopharmacol* **206**, 408-425. doi:10.1016/j.jep.2017.02.021
[457] Friedman JH (2018) Pimavanserin for Psychotic Symptoms in People With Parkinsonism: A Second Chart Review. *Clin Neuropharmacol* **41**, 156-159. doi:10.1097/wnf.0000000000000296
[458] Ikeda M, Mori E, Kosaka K, Iseki E, Hashimoto M, Matsukawa N, Matsuo K, Nakagawa M (2013) Long-term safety and efficacy of donepezil in patients with dementia with Lewy bodies: results from a 52-week, open-label, multicenter extension study. *Dement Geriatr Cogn Disord* **36**, 229-241. doi:10.1159/000351672
[459] Ikeda M, Mori E, Orimo S, Yamada T, Konishi O (2023) Efficacy of Adjunctive Therapy with Zonisamide Versus Increased Dose of Levodopa for Motor Symptoms in Patients with Dementia with Lewy Bodies: The Randomized, Controlled, Non-Inferiority DUEL Study. *J Alzheimers Dis* **95**, 251-264. doi:10.3233/jad-230335
[460] Iwasaki K, Kosaka K, Mori H, Okitsu R, Furukawa K, Manabe Y, Yoshita M, Kanamori A, Ito N, Wada K, Kitayama M, Horiguchi J, Yamaguchi S, Takayama S, Fukuhara R, Ouma S, Nakano S, Hashimoto M, Kinoshita T (2012) Improvement in delusions and hallucinations in patients with dementia with Lewy bodies upon administration of

yokukansan, a traditional Japanese medicine. *Psychogeriatrics* **12**, 235-241. doi:10.1111/j.1479-8301.2012.00413.x
[461] Lanctôt KL, Herrmann N (2000) Donepezil for behavioural disorders associated with Lewy bodies: a case series. *Int J Geriatr Psychiatry* **15**, 338-345. doi:10.1002/(sici)1099-1166(200004)15:4<338::aid-gps119>3.0.co;2-u
[462] Leopold NA (2000) Risperidone treatment of drug-related psychosis in patients with parkinsonism. *Mov Disord* **15**, 301-304. doi:10.1002/1531-8257(200003)15:2<301::aid-mds1014>3.0.co;2-2
[463] Manabe Y, Ino T, Yamanaka K, Kosaka K (2016) Increased dosage of donepezil for the management of behavioural and psychological symptoms of dementia in dementia with Lewy bodies. *Psychogeriatrics* **16**, 202-208. doi:10.1111/psyg.12140
[464] McCormick SA, Vatter S, Carter LA, Smith SJ, Orgeta V, Poliakoff E, Silverdale MA, Raw J, Ahearn DJ, Taylor C, Rodda J, Abdel-Ghany T, Kwapong B, Leroi I (2019) Parkinson's-adapted cognitive stimulation therapy: feasibility and acceptability in Lewy body spectrum disorders. *J Neurol* **266**, 1756-1770. doi:10.1007/s00415-019-09329-6
[465] Mori E, Ikeda M, Kosaka K (2012) Donepezil for dementia with Lewy bodies: a randomized, placebo-controlled trial. *Ann Neurol* **72**, 41-52. doi:10.1002/ana.23557
[466] Pakrasi S, Thomas A, Mosimann UP, Cousins DA, Lett D, Burn DJ, O'Brien JT, McKeith IG (2006) Cholinesterase inhibitors in advanced Dementia with Lewy bodies: increase or stop? *Int J Geriatr Psychiatry* **21**, 719-721. doi:10.1002/gps.1547
[467] Walker Z, Grace J, Overshot R, Satarasinghe S, Swan A, Katona CL, McKeith IG (1999) Olanzapine in dementia with Lewy bodies: a clinical study. *Int J Geriatr Psychiatry* **14**, 459-466.
[468] Caffarra P, Gardini S, Dieci F, Copelli S, Maset L, Concari L, Farina E, Grossi E (2013) The qualitative scoring MMSE pentagon test (QSPT): a new method for differentiating dementia with Lewy Body from Alzheimer's disease. *Behav Neurol* **27**, 213-220. doi:10.3233/ben-120319
[469] Cahn-Weiner DA, Williams K, Grace J, Tremont G, Westervelt H, Stern RA (2003) Discrimination of dementia with lewy bodies from Alzheimer disease and Parkinson disease using the clock drawing test. *Cogn Behav Neurol* **16**, 85-92. doi:10.1097/00146965-200306000-00001
[470] Deramecourt V, Slade JY, Oakley AE, Perry RH, Ince PG, Maurage CA, Kalaria RN (2012) Staging and natural history of cerebrovascular pathology in dementia. *Neurology* **78**, 1043-1050. doi:10.1212/WNL.0b013e31824e8e7f
[471] Donnelly PS, Sweeney A, Wilson E, Passmore AP, McCorry NK, Boeri M, Kane JPM (2024) Developing a person-centered stated preference survey for dementia with Lewy bodies: value of a personal and public involvement process. *Front Dement* **3**, 1421556. doi:10.3389/frdem.2024.1421556
[472] Duro D, Tábuas-Pereira M, Freitas S, Santiago B, Botelho MA, Santana I (2018) Validity and Clinical Utility of Different Clock Drawing Test Scoring Systems in Multiple Forms of Dementia. *J Geriatr Psychiatry Neurol* **31**, 114-122. doi:10.1177/0891988718774432

[473] Foy CM, Nicholas H, Hollingworth P, Boothby H, Willams J, Brown RG, Al-Sarraj S, Lovestone S (2007) Diagnosing Alzheimer's disease--non-clinicians and computerised algorithms together are as accurate as the best clinical practice. *Int J Geriatr Psychiatry* **22**, 1154-1163. doi:10.1002/gps.1810

[474] Galvin JE, Cohen I, Greenfield KK, Walker M (2021) The Frontal Behavioral Battery: A Measure of Frontal Lobe Symptoms in Brain Aging and Neurodegenerative Disease. *J Alzheimers Dis* **83**, 721-739. doi:10.3233/jad-210446

[475] Kim HJ, Choi KH, Kim SH, Cummings JL, Yang DW (2016) Validation Study of the Korean Version of the Brief Clinical Form of the Neuropsychiatric Inventory. *Dement Geriatr Cogn Dis Extra* **6**, 214-221. doi:10.1159/000445828

[476] Lamarre AK, Rascovsky K, Bostrom A, Toofanian P, Wilkins S, Sha SJ, Perry DC, Miller ZA, Naasan G, Laforce R, Jr., Hagen J, Takada LT, Tartaglia MC, Kang G, Galasko D, Salmon DP, Farias ST, Kaur B, Olichney JM, Quitania Park L, Mendez MF, Tsai PH, Teng E, Dickerson BC, Domoto-Reilly K, McGinnis S, Miller BL, Kramer JH (2013) Interrater reliability of the new criteria for behavioral variant frontotemporal dementia. *Neurology* **80**, 1973-1977. doi:10.1212/WNL.0b013e318293e368

[477] Matsumura K, Ichino C, Kudou Y, Tachibana N, Imamura T (2009) [REM sleep behavior disorder (RBD) in dementia with lewy bodies (DLB)--a study using short sleep-disorder questionnaire for DLB (SDQ-DLB)]. *Brain Nerve* **61**, 189-195.

[478] McCann E, Lee S, Coleman F, O'Sullivan JD, Nestor PJ (2023) Pareidolias are a function of visuoperceptual impairment. *PLoS One* **18**, e0293942. doi:10.1371/journal.pone.0293942

[479] McKeith IG, Fairbairn AF, Bothwell RA, Moore PB, Ferrier IN, Thompson P, Perry RH (1994) An evaluation of the predictive validity and inter-rater reliability of clinical diagnostic criteria for senile dementia of Lewy body type. *Neurology* **44**, 872-877. doi:10.1212/wnl.44.5.872

[480] Mitolo M, Salmon DP, Gardini S, Galasko D, Grossi E, Caffarra P (2014) The new Qualitative Scoring MMSE Pentagon Test (QSPT) as a valid screening tool between autopsy-confirmed dementia with Lewy bodies and Alzheimer's disease. *J Alzheimers Dis* **39**, 823-832. doi:10.3233/jad-131403

[481] Piersma D, Fuermaier ABM, De Waard D, Davidse RJ, De Groot J, Doumen MJA, Bredewoud RA, Claesen R, Lemstra AW, Scheltens P, Vermeeren A, Ponds R, Verhey F, De Deyn PP, Brouwer WH, Tucha O (2018) Assessing Fitness to Drive in Patients With Different Types of Dementia. *Alzheimer Dis Assoc Disord* **32**, 70-75. doi:10.1097/wad.0000000000000221

[482] Rascovsky K, Hodges JR, Knopman D, Mendez MF, Kramer JH, Neuhaus J, van Swieten JC, Seelaar H, Dopper EG, Onyike CU, Hillis AE, Josephs KA, Boeve BF, Kertesz A, Seeley WW, Rankin KP, Johnson JK, Gorno-Tempini ML, Rosen H, Prioleau-Latham CE, Lee A, Kipps CM, Lillo P, Piguet O, Rohrer JD, Rossor MN, Warren JD, Fox NC, Galasko D, Salmon DP, Black SE, Mesulam M, Weintraub S, Dickerson BC, Diehl-Schmid J, Pasquier F, Deramecourt V, Lebert F, Pijnenburg Y, Chow TW, Manes F, Grafman J, Cappa SF, Freedman M, Grossman M, Miller BL (2011) Sensitivity of revised diagnostic criteria for the behavioural variant of frontotemporal dementia. *Brain* **134**, 2456-2477. doi:10.1093/brain/awr179

[483] Stacy KE, Lambert J, Shatz R, Bakas T (2023) Development and Validation of the Lewy Body Disease Caregiver Activities Scale. *J Nurs Meas* **31**, 606-614. doi:10.1891/jnm-2021-0100
[484] Thaipisuttikul P, Chittaropas P, Wisajun P, Jullagate S (2018) Development and validation of a screening instrument for cognitive fluctuation in patients with neurocognitive disorder with Lewy bodies (NCDLB): the Mayo Fluctuations Scale-Thai version. *Gen Psychiatr* **31**, e000001. doi:10.1136/gpsych-2018-000001
[485] Thomas AJ, Taylor JP, McKeith I, Bamford C, Burn D, Allan L, O'Brien J (2017) Development of assessment toolkits for improving the diagnosis of the Lewy body dementias: feasibility study within the DIAMOND Lewy study. *Int J Geriatr Psychiatry* **32**, 1280-1304. doi:10.1002/gps.4609
[486] Uchiyama M, Nishio Y, Yokoi K, Hirayama K, Imamura T, Shimomura T, Mori E (2012) Pareidolias: complex visual illusions in dementia with Lewy bodies. *Brain* **135**, 2458-2469. doi:10.1093/brain/aws126
[487] Vatter S, McDonald KR, Stanmore E, McCormick SA, Clare L, Leroi I (2020) A brief psychometric and clinimetric evaluation of self-report burden and mental health measures completed by care partners of people with Parkinson's-related dementia. *Int Psychogeriatr* **32**, 875-880. doi:10.1017/s1041610220000605
[488] Williams DR, Warren JD, Lees AJ (2008) Using the presence of visual hallucinations to differentiate Parkinson's disease from atypical parkinsonism. *J Neurol Neurosurg Psychiatry* **79**, 652-655. doi:10.1136/jnnp.2007.124677
[489] Dada ST, Toprakcioglu Z, Cali MP, Röntgen A, Hardenberg MC, Morris OM, Mrugalla LK, Knowles TPJ, Vendruscolo M (2024) Pharmacological inhibition of α-synuclein aggregation within liquid condensates. *Nat Commun* **15**, 3835. doi:10.1038/s41467-024-47585-x
[490] Hirai Y, Fujita SC, Iwatsubo T, Hasegawa M (2004) Phosphorylated alpha-synuclein in normal mouse brain. *FEBS Lett* **572**, 227-232. doi:10.1016/j.febslet.2004.07.046
[491] Kim H, Kim BY, Soh JW, Cho EJ, Liu JO, Youn HD (2006) A novel function of Nur77: physical and functional association with protein kinase C. *Biochem Biophys Res Commun* **348**, 950-956. doi:10.1016/j.bbrc.2006.07.167
[492] Kobayashi R, Takahashi-Fujigasaki J, Shiozawa S, Hara-Miyauchi C, Inoue T, Okano HJ, Sasaki E, Okano H (2016) α-Synuclein aggregation in the olfactory bulb of middle-aged common marmoset. *Neurosci Res* **106**, 55-61. doi:10.1016/j.neures.2015.11.006
[493] Lee SS, Kim YM, Junn E, Lee G, Park KH, Tanaka M, Ronchetti RD, Quezado MM, Mouradian MM (2003) Cell cycle aberrations by alpha-synuclein over-expression and cyclin B immunoreactivity in Lewy bodies. *Neurobiol Aging* **24**, 687-696. doi:10.1016/s0197-4580(02)00196-3
[494] Maharaj DS, Maharaj H, Daya S, Glass BD (2006) Melatonin and 6-hydroxymelatonin protect against iron-induced neurotoxicity. *J Neurochem* **96**, 78-81. doi:10.1111/j.1471-4159.2005.03532.x
[495] Manfredsson FP, Luk KC, Benskey MJ, Gezer A, Garcia J, Kuhn NC, Sandoval IM, Patterson JR, O'Mara A, Yonkers R, Kordower JH (2018) Induction of alpha-synuclein pathology in the enteric nervous system of the rat and non-human primate results in

gastrointestinal dysmotility and transient CNS pathology. *Neurobiol Dis* **112**, 106-118. doi:10.1016/j.nbd.2018.01.008
[496] Park JY, Lansbury PT, Jr. (2003) Beta-synuclein inhibits formation of alpha-synuclein protofibrils: a possible therapeutic strategy against Parkinson's disease. *Biochemistry* **42**, 3696-3700. doi:10.1021/bi020604a
[497] Sorrentino ZA, Hass E, Vijayaraghavan N, Gorion KM, Riffe CJ, Dhillon JS, Giasson BI (2020) Carboxy-terminal truncation and phosphorylation of α-synuclein elongates survival in a prion-like seeding mouse model of synucleinopathy. *Neurosci Lett* **732**, 135017. doi:10.1016/j.neulet.2020.135017
[498] Stoyka LE, Arrant AE, Thrasher DR, Russell DL, Freire J, Mahoney CL, Narayanan A, Dib AG, Standaert DG, Volpicelli-Daley LA (2020) Behavioral defects associated with amygdala and cortical dysfunction in mice with seeded α-synuclein inclusions. *Neurobiol Dis* **134**, 104708. doi:10.1016/j.nbd.2019.104708
[499] Armstrong MJ, Paulson HL, Maixner SM, Fields JA, Lunde AM, Boeve BF, Manning C, Galvin JE, Taylor AS, Li Z (2021) Protocol for an observational cohort study identifying factors predicting accurately end of life in dementia with Lewy bodies and promoting quality end-of-life experiences: the PACE-DLB study. *BMJ Open* **11**, e047554. doi:10.1136/bmjopen-2020-047554
[500] Gabb VG, Blackman J, Morrison HD, Biswas B, Li H, Turner N, Russell GM, Greenwood R, Jolly A, Trender W, Hampshire A, Whone A, Coulthard E (2024) Remote Evaluation of Sleep and Circadian Rhythms in Older Adults With Mild Cognitive Impairment and Dementia: Protocol for a Feasibility and Acceptability Mixed Methods Study. *JMIR Res Protoc* **13**, e52652. doi:10.2196/52652
[501] Grycuk E, Eichenholtz E, Aarsland D, Betzhold S, Daly G, Fitzpatrick R, Folkerts AK, Kalbe E, Kane JP, Kinchin I, Saldanha IJ, Smith V, Taylor JP, Thompson R, Leroi I (2022) Developing a core outcome set (COS) for Dementia with Lewy bodies (DLB). *HRB Open Res* **5**, 57. doi:10.12688/hrbopenres.13590.2
[502] Hindle JV, Watermeyer TJ, Roberts J, Martyr A, Lloyd-Williams H, Brand A, Gutting P, Hoare Z, Edwards RT, Clare L (2016) Cognitive rehabilitation for Parkinson's disease dementia: a study protocol for a pilot randomised controlled trial. *Trials* **17**, 152. doi:10.1186/s13063-016-1253-0
[503] McCormick SA, McDonald KR, Vatter S, Orgeta V, Poliakoff E, Smith S, Silverdale MA, Fu B, Leroi I (2017) Psychosocial therapy for Parkinson's-related dementia: study protocol for the INVEST randomised controlled trial. *BMJ Open* **7**, e016801. doi:10.1136/bmjopen-2017-016801
[504] O'Brien JT, Taylor JP, Thomas A, Bamford C, Vale L, Hill S, Allan L, Finch T, McNally R, Hayes L, Surendranathan A, Kane J, Chrysos AE, Bentley A, Barker S, Mason J, Burn D, McKeith I (2021) Programme Grants for Applied Research In *Improving the diagnosis and management of Lewy body dementia: the DIAMOND-Lewy research programme including pilot cluster RCT* NIHR Journals Library
Copyright © 2021 O'Brien et al. This work was produced by O'Brien et al. under the terms of a commissioning contract issued by the Secretary of State for Health and Social Care. This is an Open Access publication distributed under the terms of the Creative Commons Attribution CC BY 4.0 licence, which permits unrestricted use,

distribution, reproduction and adaption in any medium and for any purpose provided that it is properly attributed. See: https://creativecommons.org/licenses/by/4.0/. For attribution the title, original author(s), the publication source – NIHR Journals Library, and the DOI of the publication must be cited., Southampton (UK).
[505] van Kooten J, Delwel S, Binnekade TT, Smalbrugge M, van der Wouden JC, Perez RS, Rhebergen D, Zuurmond WW, Stek ML, Lobbezoo F, Hertogh CM, Scherder EJ (2015) Pain in dementia: prevalence and associated factors: protocol of a multidisciplinary study. *BMC Geriatr* **15**, 29. doi:10.1186/s12877-015-0025-0
[506] Waddington C, Harding E, Brotherhood EV, Davies Abbott I, Barker S, Camic PM, Ezeofor V, Gardner H, Grillo A, Hardy C, Hoare Z, McKee-Jackson R, Moore K, O'Hara T, Roberts J, Rossi-Harries S, Suarez-Gonzalez A, Sullivan MP, Edwards RT, Van Der Byl Williams M, Walton J, Willoughby A, Windle G, Winrow E, Wood O, Zimmermann N, Crutch SJ, Stott J (2022) The Development of Videoconference-Based Support for People Living With Rare Dementias and Their Carers: Protocol for a 3-Phase Support Group Evaluation. *JMIR Res Protoc* **11**, e35376. doi:10.2196/35376
[507] Amini N, Ibn Hach M, Lapauw L, Dupont J, Vercauteren L, Verschueren S, Tournoy J, Gielen E (2024) Meta-analysis on the interrelationship between sarcopenia and mild cognitive impairment, Alzheimer's disease and other forms of dementia. *J Cachexia Sarcopenia Muscle* **15**, 1240-1253. doi:10.1002/jcsm.13485
[508] Areza-Fegyveres R, Caramelli P, Porto CS, Ono CR, Buchpiguel CA, Nitrini R (2007) The syndrome of progressive posterior cortical dysfunction: A multiple case study and review. *Dement Neuropsychol* **1**, 311-319. doi:10.1590/s1980-57642008dn10300014
[509] Arnold SE, Trojanowski JQ, Gur RE, Blackwell P, Han LY, Choi C (1998) Absence of neurodegeneration and neural injury in the cerebral cortex in a sample of elderly patients with schizophrenia. *Arch Gen Psychiatry* **55**, 225-232. doi:10.1001/archpsyc.55.3.225
[510] Arnulf I, Bonnet AM, Damier P, Bejjani BP, Seilhean D, Derenne JP, Agid Y (2000) Hallucinations, REM sleep, and Parkinson's disease: a medical hypothesis. *Neurology* **55**, 281-288. doi:10.1212/wnl.55.2.281
[511] Balas JS, Phelps EB, Shaw C, Washington E, Glover CM, Ludwig GA, Bennett DA, Fleischman DA, Volgman AS, Aggarwal NT (2021) Leveraging virtual reality to train certified nursing assistants as essential dementia-care personnel in the age of COVID-19. *Alzheimers Dement* **17 Suppl 11**, e051128. doi:10.1002/alz.051128
[512] Boeve BF (2013) Idiopathic REM sleep behaviour disorder in the development of Parkinson's disease. *Lancet Neurol* **12**, 469-482. doi:10.1016/s1474-4422(13)70054-1
[513] Brandel JP, Corbillé AG, Derkinderen P, Haïk S (2015) [Is Parkinson's disease a prion disease?]. *Rev Neurol (Paris)* **171**, 812-824. doi:10.1016/j.neurol.2015.10.005
[514] Brenowitz WD, Han F, Kukull WA, Nelson PT (2018) Treated hypothyroidism is associated with cerebrovascular disease but not Alzheimer's disease pathology in older adults. *Neurobiol Aging* **62**, 64-71. doi:10.1016/j.neurobiolaging.2017.10.004

[515] Cannas A, Meloni M, Mascia MM, Solla P, Cocco L, Muroni A, Floris G, Di Stefano F, Marrosu F (2017) Capgras syndrome in Parkinson's disease: two new cases and literature review. *Neurol Sci* **38**, 225-231. doi:10.1007/s10072-016-2765-9
[516] Caviness JN (2003) Myoclonus and neurodegenerative disease--what's in a name? *Parkinsonism Relat Disord* **9**, 185-192. doi:10.1016/s1353-8020(02)00054-8
[517] Caviness JN (2012) Presymptomatic Parkinson's disease: the Arizona experience. *Parkinsonism Relat Disord* **18 Suppl 1**, S203-206. doi:10.1016/s1353-8020(11)70063-3
[518] Cummings J, Emre M, Aarsland D, Tekin S, Dronamraju N, Lane R (2010) Effects of rivastigmine in Alzheimer's disease patients with and without hallucinations. *J Alzheimers Dis* **20**, 301-311. doi:10.3233/jad-2010-1362
[519] D'Este G, Berra F, Carli G, Leitner C, Marelli S, Zucconi M, Casoni F, Ferini-Strambi L, Galbiati A (2023) Cognitive Reserve in Isolated Rapid Eye-Movement Sleep Behavior Disorder. *Brain Sci* **13**. doi:10.3390/brainsci13020176
[520] Dhakal S, Kushairi N, Phan CW, Adhikari B, Sabaratnam V, Macreadie I (2019) Dietary Polyphenols: A Multifactorial Strategy to Target Alzheimer's Disease. *Int J Mol Sci* **20**. doi:10.3390/ijms20205090
[521] Dickson DW (2018) Neuropathology of Parkinson disease. *Parkinsonism Relat Disord* **46 Suppl 1**, S30-s33. doi:10.1016/j.parkreldis.2017.07.033
[522] Doi H, Tanaka F (2013) [The genetics of corticobasal syndrome]. *Brain Nerve* **65**, 19-30.
[523] Ehgoetz Martens KA, Matar E, Phillips JR, Shine JM, Grunstein RR, Halliday GM, Lewis SJG (2022) Narrow doorways alter brain connectivity and step patterns in isolated REM sleep behaviour disorder. *Neuroimage Clin* **33**, 102958. doi:10.1016/j.nicl.2022.102958
[524] Farfel JM, Nitrini R, Suemoto CK, Grinberg LT, Ferretti RE, Leite RE, Tampellini E, Lima L, Farias DS, Neves RC, Rodriguez RD, Menezes PR, Fregni F, Bennett DA, Pasqualucci CA, Jacob Filho W (2013) Very low levels of education and cognitive reserve: a clinicopathologic study. *Neurology* **81**, 650-657. doi:10.1212/WNL.0b013e3182a08f1b
[525] Farlow MR (2003) Clinical pharmacokinetics of galantamine. *Clin Pharmacokinet* **42**, 1383-1392. doi:10.2165/00003088-200342150-00005
[526] Fedorova TD, Knudsen K, Sommerauer M, Svendsen KB, Otto M, Borghammer P (2020) A Screening-Based Method for Identifying Patients with REM Sleep Behaviour Disorder in a Danish Community Setting. *J Parkinsons Dis* **10**, 1249-1253. doi:10.3233/jpd-202020
[527] Förstl H, Burns A, Levy R, Cairns N (1994) Neuropathological correlates of psychotic phenomena in confirmed Alzheimer's disease. *Br J Psychiatry* **165**, 53-59. doi:10.1192/bjp.165.1.53
[528] Gan J, Wang M, Liu S, Chen Z, Wang XD, Ji Y (2021) Effect of Multiple Medicines on Dementia Initial Treatment: Experience and Thinking. *Am J Alzheimers Dis Other Demen* **36**, 15333175211053134. doi:10.1177/15333175211053134
[529] Gibbons LE, Power MC, Walker RL, Kumar RG, Murphy A, Latimer CS, Nolan AL, Melief EJ, Beller A, Bogdani M, Keene CD, Larson EB, Crane PK, Dams-O'Connor K

(2023) Association of Traumatic Brain Injury with Late Life Neuropathological Outcomes in a Community-Based Cohort. *J Alzheimers Dis* **93**, 949-961. doi:10.3233/jad-221224
[530] Goetz CG, Vogel C, Tanner CM, Stebbins GT (1998) Early dopaminergic drug-induced hallucinations in parkinsonian patients. *Neurology* **51**, 811-814. doi:10.1212/wnl.51.3.811
[531] Gossard TR, Teigen LN, Yoo S, Timm PC, Jagielski J, Bibi N, Feemster JC, Steele T, Carvalho DZ, Junna MR, Lipford MC, Tippmann Peikert M, LeClair-Visonneau L, McCarter SJ, Boeve BF, Silber MH, Hirsch J, Sharp RR, St Louis EK (2023) Patient values and preferences regarding prognostic counseling in isolated REM sleep behavior disorder. *Sleep* **46**. doi:10.1093/sleep/zsac244
[532] Grosset DG, Tatsch K, Oertel WH, Tolosa E, Bajaj N, Kupsch A, O'Brien JT, Seibyl J, Walker Z, Sherwin P, Chen C, Grachev ID (2014) Safety analysis of 10 clinical trials and for 13 years after first approval of ioflupane 123I injection (DaTscan). *J Nucl Med* **55**, 1281-1287. doi:10.2967/jnumed.114.138032
[533] Haan MN, Jagust WJ, Galasko D, Kaye J (2002) Effect of extrapyramidal signs and Lewy bodies on survival in patients with Alzheimer disease. *Arch Neurol* **59**, 588-593. doi:10.1001/archneur.59.4.588
[534] Han W, Wei M, Xu F, Niu Z (2024) Aggregation and phase separation of α-synuclein in Parkinson's disease. *Chem Commun (Camb)* **60**, 6581-6590. doi:10.1039/d4cc01591f
[535] Hartikainen S, Rahkonen T, Kautiainen H, Sulkava R (2003) Use of psychotropics among home-dwelling nondemented and demented elderly. *Int J Geriatr Psychiatry* **18**, 1135-1141. doi:10.1002/gps.1024
[536] Honig LS, Chambliss DD, Bigio EH, Carroll SL, Elliott JL (2000) Glutamate transporter EAAT2 splice variants occur not only in ALS, but also in AD and controls. *Neurology* **55**, 1082-1088. doi:10.1212/wnl.55.8.1082
[537] Iranzo A (2022) Parasomnias and Sleep-Related Movement Disorders in Older Adults. *Sleep Med Clin* **17**, 295-305. doi:10.1016/j.jsmc.2022.02.005
[538] Kay LM (2022) COVID-19 and olfactory dysfunction: a looming wave of dementia? *J Neurophysiol* **128**, 436-444. doi:10.1152/jn.00255.2022
[539] Kiesmann M, Sauleau E, Ewald Martin R, Danan J, Sauer A, Konrad S, Blanc F, Vogel T, Kaltenbach G, Schmitt E (2022) Older Persons Displaying Parkinsonian Gait: Prediction and Explanation of the Recurrent Falling Phenomenon and Its Related Poor Prognosis. *Gerontology* **68**, 1402-1414. doi:10.1159/000521503
[540] Kiesmann M, Sauleau E, Perisse J, Jehl C, Konrad S, Karcher P, Fleury MC, Rohmer D, Sauer A, Ehret M, Vogel T, Kaltenbach G, Schmitt E (2021) Parkinsonian gait in elderly people: Significance of the threshold value of two and more falls per year. *Rev Neurol (Paris)* **177**, 385-393. doi:10.1016/j.neurol.2020.06.012
[541] Knudsen K, Fedorova TD, Hansen AK, Sommerauer M, Haase AM, Svendsen KB, Otto M, Østergaard K, Krogh K, Borghammer P (2019) Objective intestinal function in patients with idiopathic REM sleep behavior disorder. *Parkinsonism Relat Disord* **58**, 28-34. doi:10.1016/j.parkreldis.2018.08.011

[542] Kumari S, Taliyan R, Dubey SK (2023) Comprehensive Review on Potential Signaling Pathways Involving the Transfer of α-Synuclein from the Gut to the Brain That Leads to Parkinson's Disease. *ACS Chem Neurosci* **14**, 590-602. doi:10.1021/acschemneuro.2c00730

[543] Lapid MI, Burton MC, Chang MT, Rummans TA, Cha SS, Leavitt JA, Boeve BF (2013) Clinical phenomenology and mortality in Charles Bonnet syndrome. *J Geriatr Psychiatry Neurol* **26**, 3-9. doi:10.1177/0891988712473800

[544] Lerche S, Machetanz G, Roeben B, Wurster I, Zimmermann M, von Thaler AK, Liepelt-Scarfone I, Eschweiler GW, Fallgatter A, Metzger F, Maetzler W, Berg D, Brockmann K (2018) Deterioration of executive dysfunction in elderly with REM sleep behavior disorder (RBD). *Neurobiol Aging* **70**, 242-246. doi:10.1016/j.neurobiolaging.2018.06.029

[545] Lim SY, Dy Closas AMF, Tan AH, Lim JL, Tan YJ, Vijayanathan Y, Tay YW, Abdul Khalid RB, Ng WK, Kanesalingam R, Martinez-Martin P, Ahmad Annuar A, Lit LC, Foo JN, Lim WK, Ng ASL, Tan EK (2023) New insights from a multi-ethnic Asian progressive supranuclear palsy cohort. *Parkinsonism Relat Disord* **108**, 105296. doi:10.1016/j.parkreldis.2023.105296

[546] Ling H, Kearney S, Yip HL, Silveira-Moriyama L, Revesz T, Holton JL, Strand C, Davey K, Mok KY, Polke JM, Lees AJ (2016) Parkinson's disease without nigral degeneration: a pathological correlate of scans without evidence of dopaminergic deficit (SWEDD)? *J Neurol Neurosurg Psychiatry* **87**, 633-641. doi:10.1136/jnnp-2015-310756

[547] Litvan I, Campbell G, Mangone CA, Verny M, McKee A, Chaudhuri KR, Jellinger K, Pearce RK, D'Olhaberriague L (1997) Which clinical features differentiate progressive supranuclear palsy (Steele-Richardson-Olszewski syndrome) from related disorders? A clinicopathological study. *Brain* **120 (Pt 1)**, 65-74. doi:10.1093/brain/120.1.65

[548] Livinț Popa L, Dragoș HM, Strilciuc Ș, Pantelemon C, Mureșanu I, Dina C, Văcăraș V, Mureșanu D (2021) Added Value of QEEG for the Differential Diagnosis of Common Forms of Dementia. *Clin EEG Neurosci* **52**, 201-210. doi:10.1177/1550059420971122

[549] Magnaeva AS, Gulevskaya TS, Anufriev PL, Baranich TI, Sukhorukov VS (2022) [Morphological characteristics of the brain nervous tissue during aging]. *Arkh Patol* **84**, 20-28. doi:10.17116/patol20228404120

[550] Mahajan A, Bulica B, Ahmad A, Kaminski P, LeWitt P, Taylor D, Krstevska S, Patel N (2018) Pimavanserin use in a movement disorders clinic: a single-center experience. *Neurol Sci* **39**, 1767-1771. doi:10.1007/s10072-018-3500-5

[551] Mantyh WG, Block AD, Castro MR, Hansen A, Matheson MJ, Strong C, Jr., Hill A, Cayci Z, Henderson JN (2023) Characteristics of Recurrent Visions of the Nonphysical World Among Cognitively Unimpaired Elders of the Ojibwe Tribal Nation. *JAMA Netw Open* **6**, e2338221. doi:10.1001/jamanetworkopen.2023.38221

[552] Marshall A, Spreadbury J, Cheston R, Coleman P, Ballinger C, Mullee M, Pritchard J, Russell C, Bartlett E (2015) A pilot randomised controlled trial to compare changes in quality of life for participants with early diagnosis dementia who attend a 'Living

Well with Dementia' group compared to waiting-list control. *Aging Ment Health* **19**, 526-535. doi:10.1080/13607863.2014.954527
[553] McIntyre A, Harding E, Yong KXX, Sullivan MP, Gilhooly M, Gilhooly K, Woodbridge R, Crutch S (2019) Health and social care practitioners' understanding of the problems of people with dementia-related visual processing impairment. *Health Soc Care Community* **27**, 982-990. doi:10.1111/hsc.12715
[554] McShane R, Keene J, Gedling K, Fairburn C, Jacoby R, Hope T (1997) Do neuroleptic drugs hasten cognitive decline in dementia? Prospective study with necropsy follow up. *Bmj* **314**, 266-270. doi:10.1136/bmj.314.7076.266
[555] McShane R, Westby MJ, Roberts E, Minakaran N, Schneider L, Farrimond LE, Maayan N, Ware J, Debarros J (2019) Memantine for dementia. *Cochrane Database Syst Rev* **3**, Cd003154. doi:10.1002/14651858.CD003154.pub6
[556] Molloy SA, Rowan EN, O'Brien JT, McKeith IG, Wesnes K, Burn DJ (2006) Effect of levodopa on cognitive function in Parkinson's disease with and without dementia and dementia with Lewy bodies. *J Neurol Neurosurg Psychiatry* **77**, 1323-1328. doi:10.1136/jnnp.2006.098079
[557] Mrak RE (2009) Neuropathology and the neuroinflammation idea. *J Alzheimers Dis* **18**, 473-481. doi:10.3233/jad-2009-1158
[558] Nakamori M, Junn E, Mochizuki H, Mouradian MM (2019) Nucleic Acid-Based Therapeutics for Parkinson's Disease. *Neurotherapeutics* **16**, 287-298. doi:10.1007/s13311-019-00714-7
[559] Nishio Y, Yokoi K, Hirayama K, Ishioka T, Hosokai Y, Gang M, Uchiyama M, Baba T, Suzuki K, Takeda A, Mori E (2018) Defining visual illusions in Parkinson's disease: Kinetopsia and object misidentification illusions. *Parkinsonism Relat Disord* **55**, 111-116. doi:10.1016/j.parkreldis.2018.05.023
[560] Oda S, Sano T, Nishikawa N, Mikasa M, Takahashi Y, Takao M (2021) [Amyotrophic lateral sclerosis with muscle weakness and dropped head during the course of Parkinson's disease: an autopsy case]. *Rinsho Shinkeigaku* **61**, 373-377. doi:10.5692/clinicalneurol.cn-001546
[561] Oudiette D, De Cock VC, Lavault S, Leu S, Vidailhet M, Arnulf I (2009) Nonviolent elaborate behaviors may also occur in REM sleep behavior disorder. *Neurology* **72**, 551-557. doi:10.1212/01.wnl.0000341936.78678.3a
[562] Pagonabarraga J, Llebaria G, García-Sánchez C, Pascual-Sedano B, Gironell A, Kulisevsky J (2008) A prospective study of delusional misidentification syndromes in Parkinson's disease with dementia. *Mov Disord* **23**, 443-448. doi:10.1002/mds.21864
[563] Pakrasi S, Mukaetova-Ladinska EB, McKeith IG, O'Brien JT (2003) Clinical predictors of response to Acetyl Cholinesterase Inhibitors: experience from routine clinical use in Newcastle. *Int J Geriatr Psychiatry* **18**, 879-886. doi:10.1002/gps.928
[564] Park IS, Yoo SW, Lee KS, Kim JS (2014) Epileptic seizure presenting as dementia with Lewy bodies. *Gen Hosp Psychiatry* **36**, 230.e233-235. doi:10.1016/j.genhosppsych.2013.10.015
[565] Park J, Howard H, Tolea MI, Galvin JE (2020) Perceived Benefits of Using Nonpharmacological Interventions in Older Adults With Alzheimer's Disease or

Dementia With Lewy Bodies. *J Gerontol Nurs* **46**, 37-46. doi:10.3928/00989134-20191217-01
[566] Qian W, Fischer CE, Schweizer TA, Munoz DG (2018) Association Between Psychosis Phenotype and APOE Genotype on the Clinical Profiles of Alzheimer's Disease. *Curr Alzheimer Res* **15**, 187-194. doi:10.2174/1567205014666170829114346
[567] Ratti PL, Sierra-Peña M, Manni R, Simonetta-Moreau M, Bastin J, Mace H, Rascol O, David O (2015) Distinctive features of NREM parasomnia behaviors in parkinson's disease and multiple system atrophy. *PLoS One* **10**, e0120973. doi:10.1371/journal.pone.0120973
[568] Reckner E, Cipolotti L, Foley JA (2020) Presence phenomena in parkinsonian disorders: Phenomenology and neuropsychological correlates. *Int J Geriatr Psychiatry* **35**, 785-793. doi:10.1002/gps.5303
[569] Rockwood K, Fay S, Hamilton L, Ross E, Moorhouse P (2014) Good days and bad days in dementia: a qualitative chart review of variable symptom expression. *Int Psychogeriatr* **26**, 1239-1246. doi:10.1017/s1041610214000222
[570] Ross GW, Abbott RD, Petrovitch H, Tanner CM, White LR (2012) Pre-motor features of Parkinson's disease: the Honolulu-Asia Aging Study experience. *Parkinsonism Relat Disord* **18 Suppl 1**, S199-202. doi:10.1016/s1353-8020(11)70062-1
[571] Ruggiero F, Zirone E, Molisso MT, Carandini T, Fumagalli G, Pietroboni A, Ferrucci R, Aiello EN, Poletti B, Silani V, Comi G, Scarpini E, Barbieri S, Arighi A, Mameli F (2023) Telemedicine for cognitive impairment: a telephone survey of patients' experiences with neurological video consultation. *Neurol Sci* **44**, 3885-3894. doi:10.1007/s10072-023-06903-9
[572] Sakurai K, Kaneda D, Uchida Y, Inui S, Bundo M, Akagi A, Nihashi T, Kimura Y, Kato T, Ito K, Ohashi W, Hashizume Y (2021) Can Medial Temporal Impairment Be an Imaging Red Flag for Neurodegeneration in Disproportionately Enlarged Subarachnoid Space Hydrocephalus? *J Alzheimers Dis* **83**, 1199-1209. doi:10.3233/jad-210535
[573] Sarto J, Mayà G, Molina-Porcel L, Balasa M, Gelpi E, Aldecoa I, Borrego-Écija S, Contador J, Ximelis T, Vergara M, Antonell A, Sánchez-Valle R, Lladó A (2022) Evolution of Clinical-Pathological Correlations in Early-Onset Alzheimer's Disease Over a 25-Year Period in an Academic Brain Bank. *J Alzheimers Dis* **87**, 1659-1669. doi:10.3233/jad-220045
[574] Sharma S, Awasthi A, Singh S (2019) Altered gut microbiota and intestinal permeability in Parkinson's disease: Pathological highlight to management. *Neurosci Lett* **712**, 134516. doi:10.1016/j.neulet.2019.134516
[575] Shindo A, Ueda Y, Kuzuhara S, Kokubo Y (2014) Neuropsychological study of amyotrophic lateral sclerosis and parkinsonism-dementia complex in Kii peninsula, Japan. *BMC Neurol* **14**, 151. doi:10.1186/1471-2377-14-151
[576] Sohail S, Yu L, Schneider JA, Bennett DA, Buchman AS, Lim ASP (2017) Sleep fragmentation and Parkinson's disease pathology in older adults without Parkinson's disease. *Mov Disord* **32**, 1729-1737. doi:10.1002/mds.27200
[577] Tachibana H (2013) [Cognitive impairment in Parkinson's disease]. *Seishin Shinkeigaku Zasshi* **115**, 1142-1149.

[578] Tolea MI, Camacho S, Cohen IR, Galvin JE (2023) Mindfulness and Care Experience in Family Caregivers of Persons Living with Dementia. *J Alzheimers Dis Rep* **7**, 151-164. doi:10.3233/adr-220069

[579] Tuena C, Riva G, Murru I, Campana L, Goulene KM, Pedroli E, Stramba-Badiale M (2022) Contribution of cognitive and bodily navigation cues to egocentric and allocentric spatial memory in hallucinations due to Parkinson's disease: A case report. *Front Behav Neurosci* **16**, 992498. doi:10.3389/fnbeh.2022.992498

[580] Uchiyama M, Nishio Y, Yokoi K, Hosokai Y, Takeda A, Mori E (2015) Pareidolia in Parkinson's disease without dementia: A positron emission tomography study. *Parkinsonism Relat Disord* **21**, 603-609. doi:10.1016/j.parkreldis.2015.03.020

[581] Uitti RJ (2012) Tandem deep brain stimulation--challenging new structural targets for Parkinson's disease. *Parkinsonism Relat Disord* **18 Suppl 1**, S171-173. doi:10.1016/s1353-8020(11)70053-0

[582] Verbaan D, van Rooden SM, Visser M, Marinus J, Emre M, van Hilten JJ (2009) Psychotic and compulsive symptoms in Parkinson's disease. *Mov Disord* **24**, 738-744. doi:10.1002/mds.22453

[583] Willroth EC, James BD, Graham EK, Kapasi A, Bennett DA, Mroczek DK (2023) Well-Being and Cognitive Resilience to Dementia-Related Neuropathology. *Psychol Sci* **34**, 283-297. doi:10.1177/09567976221119828

[584] Wüllner U, Borghammer P, Choe CU, Csoti I, Falkenburger B, Gasser T, Lingor P, Riederer P (2023) The heterogeneity of Parkinson's disease. *J Neural Transm (Vienna)* **130**, 827-838. doi:10.1007/s00702-023-02635-4

[585] Zis P, Mitsikostas DD (2018) Nocebo Responses in Brain Diseases: A Systematic Review of the Current Literature. *Int Rev Neurobiol* **139**, 443-462. doi:10.1016/bs.irn.2018.07.025

[586] Alafuzoff I (2018) Minimal neuropathologic diagnosis for brain banking in the normal middle-aged and aged brain and in neurodegenerative disorders. *Handb Clin Neurol* **150**, 131-141. doi:10.1016/b978-0-444-63639-3.00010-4

[587] Alencar Filho EB, Ribeiro LAA, Carvalho TGC, Silva FS, Duarte-Filho L, Barbosa EG, Menezes PMN, Tavares JF, da Silva MS, Silva BA (2021) In vitro and in silico studies of 8(17),12E,14-labdatrien-18-oic acid in airways smooth muscle relaxation: new molecular insights about its mechanism of action. *Naunyn Schmiedebergs Arch Pharmacol* **394**, 885-902. doi:10.1007/s00210-020-02010-0

[588] An H, Sung W, Yoon SY (2022) Implementation of learning by design in a synchronized online environment to teach educational robotics to inservice teachers. *Educ Technol Res Dev* **70**, 1473-1496. doi:10.1007/s11423-022-10134-8

[589] Beattie JH, Peace HS (1993) The influence of a low-boron diet and boron supplementation on bone, major mineral and sex steroid metabolism in postmenopausal women. *Br J Nutr* **69**, 871-884. doi:10.1079/bjn19930087

[590] Berger-Sieczkowski E, Lutz MI, Auff E, Kovacs GG (2016) Gaucher cells are not associated with α-synuclein neuropathology in infants. *Clin Neuropathol* **35**, 122-128. doi:10.5414/np300901

[591] Bonauto DK, Smith CK, Adams DA, Fan ZJ, Silverstein BA, Foley MP (2010) Language preference and non-traumatic low back disorders in Washington State workers' compensation. *Am J Ind Med* **53**, 204-215. doi:10.1002/ajim.20740
[592] Borod JC, Koff E, Perlman Lorch M, Nicholas M (1986) The expression and perception of facial emotion in brain-damaged patients. *Neuropsychologia* **24**, 169-180. doi:10.1016/0028-3932(86)90050-3
[593] Borod JC, Rorie KD, Haywood CS, Andelman F, Obler LK, Welkowitz J, Bloom RL, Tweedy JR (1996) Hemispheric specialization for discourse reports of emotional experiences: relationships to demographic, neurological, and perceptual variables. *Neuropsychologia* **34**, 351-359. doi:10.1016/0028-3932(95)00131-x
[594] Boukrina O, Chen P (2021) Neural Mechanisms of Prism Adaptation in Healthy Adults and Individuals with Spatial Neglect after Unilateral Stroke: A Review of fMRI Studies. *Brain Sci* **11**. doi:10.3390/brainsci11111468
[595] Carson L, Filipowicz A, Anderson B, Danckert J (2019) Representational drawing following brain injury. *Neuropsychologia* **133**, 107154. doi:10.1016/j.neuropsychologia.2019.107154
[596] Chen CL, Kaber DB, Dempsey PG (2000) A new approach to applying feedforward neural networks to the prediction of musculoskeletal disorder risk. *Appl Ergon* **31**, 269-282. doi:10.1016/s0003-6870(99)00055-1
[597] Cherniack M, Dillon C, Erdil M, Ferguson S, Kaplan J, Krompinger J, Litt M, Murphy M (2001) Clinical and psychological correlates of lumbar motion abnormalities in low back disorders. *Spine J* **1**, 290-298. doi:10.1016/s1529-9430(01)00104-8
[598] Cho EM, Lee HS, Moon JS, Kim IS, Sim S, Ohta A (2012) Organotin compounds act as inhibitor of transcriptional activation with human estrogen receptor. *J Microbiol Biotechnol* **22**, 378-384. doi:10.4014/jmb.1105.05033
[599] Chou Y, Yang RJ (2009) Simulations of IEF in microchannel with variable cross-sectional area. *Electrophoresis* **30**, 819-830. doi:10.1002/elps.200800460
[600] Clarençon F, Papagiannaki C, Saleme S, Rouchaud A, Shotar E, Rius E, Burel J, Boch AL, Sourour NA, Mounayer C (2023) Balloon Pressure Technique with the Scepter Mini Balloon as Part of the Endovascular Strategy for Brain Arteriovenous Malformations Embolization : Preliminary Multicenter Experience. *Clin Neuroradiol* **33**, 1055-1065. doi:10.1007/s00062-023-01309-8
[601] Clarençon F, Parat D, Shotar E, Premat K, Lenck S, Drir M, Maillart E, Boch AL, Sourour N (2023) 'Balloon pressure technique' for endovascular treatment of spinal cord arteriovenous fistulas: preliminary results in 10 cases. *J Neurointerv Surg* **15**, 276-282. doi:10.1136/neurintsurg-2022-018807
[602] Cosentino L, Heddle JA (2000) Differential mutation of transgenic and endogenous loci in vivo. *Mutat Res* **454**, 1-10. doi:10.1016/s0027-5107(00)00125-1
[603] de Bonis M, Dellatolas G, Rondot P (1985) Mood disorders in left and right brain-damaged patients: comparison between ratings and self-ratings on the same adjective mood scale. Some methodological problems. *Psychopathology* **18**, 286-292. doi:10.1159/000284416
[604] Dellafiora L, Ruotolo R, Perotti A, Cirlini M, Galaverna G, Cozzini P, Buschini A, Dall'Asta C (2017) Molecular insights on xenoestrogenic potential of zearalenone-

14-glucoside through a mixed in vitro/in silico approach. *Food Chem Toxicol* **108**, 257-266. doi:10.1016/j.fct.2017.07.062
[605] Dodamani MH, Lila AR, Memon SS, Sarathi V, Arya S, Rane A, Sehemby MK, Garg R, Bhandare VV, Karlekar M, Patil VA, Kunwar A, Bandgar TR (2023) Genotypic Spectrum and its Correlation with Alopecia and Clinical Response in Hereditary Vitamin D Resistant Rickets: Our Experience and Systematic Review. *Calcif Tissue Int* **112**, 483-492. doi:10.1007/s00223-023-01061-8
[606] Dowhan DH, Muscat GE (1996) Characterization of the AB (AF-1) region in the muscle-specific retinoid X receptor-gamma: evidence that the AF-1 region functions in a cell-specific manner. *Nucleic Acids Res* **24**, 264-271. doi:10.1093/nar/24.2.264
[607] Eitelhuber TW, Thackray J, Hodges S, Alan J (2018) Fit for purpose - developing a software platform to support the modern challenges of data linkage in Western Australia. *Int J Popul Data Sci* **3**, 435. doi:10.23889/ijpds.v3i3.435
[608] Espay AJ, Da Prat GA, Dwivedi AK, Rodriguez-Porcel F, Vaughan JE, Rosso M, Devoto JL, Duker AP, Masellis M, Smith CD, Mandybur GT, Merola A, Lang AE (2017) Deconstructing normal pressure hydrocephalus: Ventriculomegaly as early sign of neurodegeneration. *Ann Neurol* **82**, 503-513. doi:10.1002/ana.25046
[609] Espinoza-Ríos J, Aliaga Ramos M, Rodríguez Borda J, Miraval Wong E, Zegarra Chang A, Bravo Paredes E, Prochazka Zárate R (2016) [Sphincterotomy followed by papillary large balloon in the management of cholecolithiasis. Therapeutic success and safety in a Lima-Peru hospital]. *Rev Gastroenterol Peru* **36**, 203-208.
[610] Fernandez-Gomez FJ, Jumeau F, Derisbourg M, Burnouf S, Tran H, Eddarkaoui S, Obriot H, Dutoit-Lefevre V, Deramecourt V, Mitchell V, Lefranc D, Hamdane M, Blum D, Buée L, Buée-Scherrer V, Sergeant N (2014) Consensus brain-derived protein, extraction protocol for the study of human and murine brain proteome using both 2D-DIGE and mini 2DE immunoblotting. *J Vis Exp*. doi:10.3791/51339
[611] Gagnon D, Plamondon A, Larivière C (2016) A biomechanical comparison between expert and novice manual materials handlers using a multi-joint EMG-assisted optimization musculoskeletal model of the lumbar spine. *J Biomech* **49**, 2938-2945. doi:10.1016/j.jbiomech.2016.07.009
[612] Ganguly RK, Al-Helal MA, Chakraborty SK (2022) Role of bioactive xenobiotics towards reproductive potential of Odontotermes longignathus through in silico study: An amalgamation of ecoinformatics and ecotechnological insights of termite mounds from a tropical forest, India. *Ecotoxicol Environ Saf* **232**, 113275. doi:10.1016/j.ecoenv.2022.113275
[613] Goldenberg G (1996) Defective imitation of gestures in patients with damage in the left or right hemispheres. *J Neurol Neurosurg Psychiatry* **61**, 176-180. doi:10.1136/jnnp.61.2.176
[614] Goldstein DS, Cheshire WP, Jr. (2018) Roles of cardiac sympathetic neuroimaging in autonomic medicine. *Clin Auton Res* **28**, 397-410. doi:10.1007/s10286-018-0547-6
[615] Graham LD, Pilling PA, Eaton RE, Gorman JJ, Braybrook C, Hannan GN, Pawlak-Skrzecz A, Noyce L, Lovrecz GO, Lu L, Hill RJ (2007) Purification and characterization of recombinant ligand-binding domains from the ecdysone receptors of four pest insects. *Protein Expr Purif* **53**, 309-324. doi:10.1016/j.pep.2006.12.011

[616] Guan T, Sun Y, Wang Y, Li Z, Li T, Hou L (2019) Multi-Residue Method for the Analysis of Stilbene Estrogens in Milk. *Int J Mol Sci* **20**. doi:10.3390/ijms20030744
[617] Hallgren J, Pietrzak M, Rempala G, Nelson PT, Hetman M (2014) Neurodegeneration-associated instability of ribosomal DNA. *Biochim Biophys Acta* **1842**, 860-868. doi:10.1016/j.bbadis.2013.12.012
[618] Hamdan S, Melhem NM, Porta G, Walker Payne M, Brent DA (2012) The phenomenology and course of depression in parentally bereaved and non-bereaved youth. *J Am Acad Child Adolesc Psychiatry* **51**, 528-536. doi:10.1016/j.jaac.2012.02.021
[619] Hatton C, Ghanem SS, Koss DJ, Abdi IY, Gibbons E, Guerreiro R, Bras J, Walker L, Gelpi E, Heywood W, Outeiro TF, Attems J, McFarland R, Forsyth R, El-Agnaf OM, Erskine D (2022) Prion-like α-synuclein pathology in the brain of infants with Krabbe disease. *Brain* **145**, 1257-1263. doi:10.1093/brain/awac002
[620] Hickey TE, Irvine CM, Dvinge H, Tarulli GA, Hanson AR, Ryan NK, Pickering MA, Birrell SN, Hu DG, Mackenzie PI, Russell R, Caldas C, Raj GV, Dehm SM, Plymate SR, Bradley RK, Tilley WD, Selth LA (2015) Expression of androgen receptor splice variants in clinical breast cancers. *Oncotarget* **6**, 44728-44744. doi:10.18632/oncotarget.6296
[621] Hollister RD, Xia M, McNamara MJ, Hyman BT (1997) Neuronal expression of class II major histocompatibility complex (HLA-DR) in 2 cases of Pick disease. *Arch Neurol* **54**, 243-248. doi:10.1001/archneur.1997.00550150011008
[622] Huang WC, Chang MC, Wang WF, Jhang KM (2023) Corrigendum: A comparison of caregiver burden for different types of dementia: an 18-month retrospective cohort study. *Front Psychol* **14**, 1224716. doi:10.3389/fpsyg.2023.1224716
[623] Huisman-Baron M, van der Veen L, Jansen PA, van Roon EN, Brouwers JR, van Marum RJ (2011) Criteria for drug selection in frail elderly persons. *Drugs Aging* **28**, 391-402. doi:10.2165/11587200-000000000-00000
[624] Hurth KM, Nilges MJ, Carlson KE, Tamrazi A, Belford RL, Katzenellenbogen JA (2004) Ligand-induced changes in estrogen receptor conformation as measured by site-directed spin labeling. *Biochemistry* **43**, 1891-1907. doi:10.1021/bi035566p
[625] Jansen C, Schuur M, Spliet WG, van Gool WA, van Duijn CM, Rozemuller AJ (2009) [Eleven years of autopsy on account of Creutzfeldt-Jakob disease in the Netherlands]. *Ned Tijdschr Geneeskd* **153**, A172.
[626] Jin X, Liu GG, Luo N, Li H, Guan H, Xie F (2016) Is bad living better than good death? Impact of demographic and cultural factors on health state preference. *Qual Life Res* **25**, 979-986. doi:10.1007/s11136-015-1129-x
[627] Karamyan A, Brandtner H, Grinzinger S, Chroust V, Bacher C, Otto F, Reisp M, Hauer L, Sellner J (2017) Causes of death in critically ill multiple sclerosis patients. *Acta Neurol Scand* **136**, 305-309. doi:10.1111/ane.12724
[628] Kashyap J, Tyagi RK (2022) Mitotic genome bookmarking by nuclear receptor VDR advocates transmission of cellular transcriptional memory to progeny cells. *Exp Cell Res* **417**, 113193. doi:10.1016/j.yexcr.2022.113193

[629] Kawada M, Tanaka N, Yamaguchi S, Meguro K (2014) Observational assessment of communication disorders in vascular dementia patients with right hemisphere damage. *Psychogeriatrics* **14**, 143-151. doi:10.1111/psyg.12052

[630] Kelley AR, Colley ME, Perry G, Bach SBH (2018) Incubation with Cu(II) and Zn(II) salts enhances MALDI-TOF mass spectra of amyloid-beta and α-synuclein toward in vivo analysis. *J Mass Spectrom* **53**, 162-171. doi:10.1002/jms.4044

[631] Khandelwal A, Krasowski MD, Reschly EJ, Sinz MW, Swaan PW, Ekins S (2008) Machine learning methods and docking for predicting human pregnane X receptor activation. *Chem Res Toxicol* **21**, 1457-1467. doi:10.1021/tx800102e

[632] Krishnan A, Degnan BM, Degnan SM (2019) The first identification of complete Eph-ephrin signalling in ctenophores and sponges reveals a role for neofunctionalization in the emergence of signalling domains. *BMC Evol Biol* **19**, 96. doi:10.1186/s12862-019-1418-z

[633] Lacal J, Alfonso C, Liu X, Parales RE, Morel B, Conejero-Lara F, Rivas G, Duque E, Ramos JL, Krell T (2010) Identification of a chemoreceptor for tricarboxylic acid cycle intermediates: differential chemotactic response towards receptor ligands. *J Biol Chem* **285**, 23126-23136. doi:10.1074/jbc.M110.110403

[634] Launer-Wachs S, Taub-Tabib H, Madem JT, Bar-Natan O, Goldberg Y, Shamay Y (2023) From centralized to ad-hoc knowledge base construction for hypotheses generation. *J Biomed Inform* **142**, 104383. doi:10.1016/j.jbi.2023.104383

[635] Lee GS, Simons SS, Jr. (2011) Ligand binding domain mutations of the glucocorticoid receptor selectively modify the effects with, but not binding of, cofactors. *Biochemistry* **50**, 356-366. doi:10.1021/bi101792d

[636] Mandal PK, Sarifuddin, Kolachalama VB (2016) Computational Model of Drug-Coated Balloon Delivery in a Patient-Specific Arterial Vessel with Heterogeneous Tissue Composition. *Cardiovasc Eng Technol* **7**, 406-419. doi:10.1007/s13239-016-0273-y

[637] Martín-Mora D, Ortega Á, Matilla MA, Martínez-Rodríguez S, Gavira JA, Krell T (2019) The Molecular Mechanism of Nitrate Chemotaxis via Direct Ligand Binding to the PilJ Domain of McpN. *mBio* **10**. doi:10.1128/mBio.02334-18

[638] Maski K, Trotti LM, Kotagal S, Robert Auger R, Rowley JA, Hashmi SD, Watson NF (2021) Treatment of central disorders of hypersomnolence: an American Academy of Sleep Medicine clinical practice guideline. *J Clin Sleep Med* **17**, 1881-1893. doi:10.5664/jcsm.9328

[639] Mei F, Rota M, Bonifazi M, Zuccatosta L, Porcarelli FM, Sediari M, Bedawi EO, Sundaralingam A, Addala D, Gasparini S, Rahman NM (2023) Efficacy of Small versus Large-Bore Chest Drain in Pleural Infection: A Systematic Review and Meta-Analysis. *Respiration* **102**, 247-256. doi:10.1159/000529027

[640] Montreys CR, Borod JC (1998) A preliminary evaluation of emotional experience and expression following unilateral brain damage. *Int J Neurosci* **96**, 269-283. doi:10.3109/00207459808986474

[641] Morimura Y, Hirano A, Llena JF (1985) Electron-microscopic observation of the nucleus basilis of Meynert in human autopsy cases. *Acta Neuropathol* **68**, 130-137. doi:10.1007/bf00688634

[642] Muñoz JP, Araya-Osorio R, Mera-Adasme R, Calaf GM (2023) Glyphosate mimics 17β-estradiol effects promoting estrogen receptor alpha activity in breast cancer cells. *Chemosphere* **313**, 137201. doi:10.1016/j.chemosphere.2022.137201

[643] Najim O, Seghers S, Sergoynne L, Van Gaver H, Papadimitriou K, Wouters K, Trinh XB, Huizing MT, Tjalma W (2019) The association between type of endocrine therapy and development of estrogen receptor-1 mutation(s) in patients with hormone-sensitive advanced breast cancer: A systematic review and meta-analysis of randomized and non-randomized trials. *Biochim Biophys Acta Rev Cancer* **1872**, 188315. doi:10.1016/j.bbcan.2019.188315

[644] Nakhutina L, Borod JC, Zgaljardic DJ (2006) Posed prosodic emotional expression in unilateral stroke patients: recovery, lesion location, and emotional perception. *Arch Clin Neuropsychol* **21**, 1-13. doi:10.1016/j.acn.2005.06.013

[645] Obst JK, Tien AH, Setiawan JC, Deneault LF, Sadar MD (2024) Inhibitors of the transactivation domain of androgen receptor as a therapy for prostate cancer. *Steroids* **210**, 109482. doi:10.1016/j.steroids.2024.109482

[646] Ohishi S, Otani T (2023) Preimplantation genetic testing for aneuploidy: helpful but not a first choice. *J Assist Reprod Genet* **40**, 161-168. doi:10.1007/s10815-022-02683-x

[647] Oleske DM, Lavender SA, Andersson GB, Kwasny MM (2007) Are back supports plus education more effective than education alone in promoting recovery from low back pain?: Results from a randomized clinical trial. *Spine (Phila Pa 1976)* **32**, 2050-2057. doi:10.1097/BRS.0b013e3181453fcc

[648] Pepe P, Vatrano S, Cannarella R, Calogero AE, Marchese G, Ravo M, Fraggetta F, Pepe L, Pennisi M, Romano C, Ferri R, Salemi M (2021) A study of gene expression by RNA-seq in patients with prostate cancer and in patients with Parkinson disease: an example of inverse comorbidity. *Mol Biol Rep* **48**, 7627-7631. doi:10.1007/s11033-021-06723-0

[649] Randerath J, Li Y, Goldenberg G, Hermsdörfer J (2009) Grasping tools: effects of task and apraxia. *Neuropsychologia* **47**, 497-505. doi:10.1016/j.neuropsychologia.2008.10.005

[650] Rane SU, Shet T, Sridhar E, Bhele S, Gaikwad V, Agale S, Shinde S, Prabhat D, Fernandes G, Hastak M, Tampi C, Narurkar S, Patel K, Madiwale C, Shah K, Shah L, Sawaimoon S, Lad P (2014) Interobserver variation is a significant limitation in the diagnosis of Burkitt lymphoma. *Indian J Med Paediatr Oncol* **35**, 44-53. doi:10.4103/0971-5851.133721

[651] Sadar MD (2012) Advances in small molecule inhibitors of androgen receptor for the treatment of advanced prostate cancer. *World J Urol* **30**, 311-318. doi:10.1007/s00345-011-0745-5

[652] Sadiq S, Rana NF, Zahid MA, Zargaham MK, Tanweer T, Batool A, Naeem A, Nawaz A, Rizwan Ur R, Muneer Z, Siddiqi AR (2020) Virtual Screening of FDA-Approved Drugs against LasR of Pseudomonas aeruginosa for Antibiofilm Potential. *Molecules* **25**. doi:10.3390/molecules25163723

[653] Sakakibara R, Yamamoto T, Sekido N, Sawai S (2023) How brain diseases affect the lower urinary tract function? *Bladder (San Franc)* **10**, e21200001. doi:10.14440/bladder.2023.854

[654] Sartori S, Poirrier R (1996) [Seasonal affective syndrome and phototherapy: theoretical concepts and clinical applications]. *Encephale* **22**, 7-16.

[655] Sedlák D, Paguio A, Bartůněk P (2011) Two panels of steroid receptor luciferase reporter cell lines for compound profiling. *Comb Chem High Throughput Screen* **14**, 248-266. doi:10.2174/138620711795222446

[656] Sternberg RM, Hotchkiss AK, Leblanc GA (2008) Synchronized expression of retinoid X receptor mRNA with reproductive tract recrudescence in an imposex-susceptible mollusc. *Environ Sci Technol* **42**, 1345-1351. doi:10.1021/es702381g

[657] Suzuki H, Quintáns J (1986) The syngeneic T-T lymphocyte reaction (STTLR). II. Induction of primary T anti-T cell cytotoxic responses in vitro in T cell cultures stimulated with syngeneic self-reactive T cells. *J Mol Cell Immunol* **2**, 345-357.

[658] Takaya M (2013) Memantine treatment for neuropsychiatric symptoms in a patient with probable idiopathic normal pressure hydrocephalus: a case report. *J Med Case Rep* **7**, 94. doi:10.1186/1752-1947-7-94

[659] Testempasis SI, Papazlatani CV, Theocharis S, Karas PA, Koundouras S, Karpouzas DG, Karaoglanidis GS (2023) Vineyard practices reduce the incidence of Aspergillus spp. and alter the composition of carposphere microbiome in grapes (Vitis vinifera L.). *Front Microbiol* **14**, 1257644. doi:10.3389/fmicb.2023.1257644

[660] Tomokane N, Iwaki T, Tateishi J, Iwaki A, Goldman JE (1991) Rosenthal fibers share epitopes with alpha B-crystallin, glial fibrillary acidic protein, and ubiquitin, but not with vimentin. Immunoelectron microscopy with colloidal gold. *Am J Pathol* **138**, 875-885.

[661] Trojano L, Caccavale M, De Bellis F, Crisci C (2017) The brain and the subjective experience of time. A voxel based symptom-lesion mapping study. *Behav Brain Res* **329**, 26-34. doi:10.1016/j.bbr.2017.04.031

[662] Villardita C (1987) Tactile exploration of space and visual neglect in brain-damaged patients. *J Neurol* **234**, 292-297. doi:10.1007/bf00314283

[663] Vu JP, Jagannath D, Spielberg DR, Chiou EH, Hosek KE, Lambert EM (2024) Triple endoscopy and recurrent croup in children: A single aerodigestive center experience. *Auris Nasus Larynx* **51**, 125-131. doi:10.1016/j.anl.2023.07.005

[664] Wainwright-Sharp JA, Bryson SE (1993) Visual orienting deficits in high-functioning people with autism. *J Autism Dev Disord* **23**, 1-13. doi:10.1007/bf01066415

[665] Wang TT, Si FL, He ZB, Chen B (2018) Genome-wide identification, characterization and classification of ionotropic glutamate receptor genes (iGluRs) in the malaria vector Anopheles sinensis (Diptera: Culicidae). *Parasit Vectors* **11**, 34. doi:10.1186/s13071-017-2610-x

[666] Zhang X, Jeyakumar M, Bagchi MK (1996) Ligand-dependent cross-talk between steroid and thyroid hormone receptors. Evidence for common transcriptional coactivator(s). *J Biol Chem* **271**, 14825-14833.

[667] Zhou NX, Huang ZQ, Zhang WZ, Huang XQ, Wang J, Liu R, Ji WB, Xiao M, Meng XF (2006) [Surgical treatment of 402 consecutive cases for hilar cholangiocarcinoma: Chinese single center experience]. *Zhonghua Wai Ke Za Zhi* **44**, 1599-1603.
[668] Agarwal K, Backler W, Bayram E, Bloom L, Boeve BF, Cha JH, Denslow M, Ferman TJ, Galasko D, Galvin JE, Gomperts SN, Irizarry MC, Kantarci K, Kaushik H, Kietlinski M, Koenig A, Leverenz JB, McKeith I, McLean PJ, Montine TJ, Moose SO, O'Brien JT, Panier V, Ramanathan S, Ringel MS, Scholz SW, Small J, Sperling RA, Taylor A, Taylor JP, Ward RA, Witten L, Hyman BT (2024) Lewy body dementia: Overcoming barriers and identifying solutions. *Alzheimers & Dementia* **20**, 2298-2308. doi:10.1002/alz.13674
[669] Alosco ML, Mez J, Tripodis Y, Kiernan PT, Abdolmohammadi B, Murphy L, Kowall NW, Stein TD, Huber BR, Goldstein LE, Cantu RC, Katz DI, Chaisson CE, Martin B, Solomon TM, McClean MD, Daneshvar DH, Nowinski CJ, Stern RA, McKee AC (2018) Age of first exposure to tackle football and chronic traumatic encephalopathy. *Annals of Neurology* **83**, 886-901. doi:10.1002/ana.25245
[670] Amini N, Ibn Hach M, Lapauw L, Dupont J, Vercauteren L, Verschueren S, Tournoy J, Gielen E (2024) Meta-analysis on the interrelationship between sarcopenia and mild cognitive impairment, Alzheimer's disease and other forms of dementia. *Journal of Cachexia Sarcopenia and Muscle* **15**, 1240-1253. doi:10.1002/jcsm.13485
[671] Armstrong MJ, Alliance S, Corsentino P, Lunde A, Taylor A (2022) Informal caregiver experiences at the end-of-life of individuals living with dementia with Lewy bodies: An interview study. *Dementia-International Journal of Social Research and Practice* **21**, 287-303. doi:10.1177/14713012211038428
[672] Armstrong MJ, Alliance S, Corsentino P, Maixner SM, Paulson HL, Taylor A (2020) Caregiver-reported barriers to quality end-of-life care in dementia with Lewy bodies: A qualitative analysis. *American Journal of Hospice & Palliative Medicine* **37**, 728-737. doi:10.1177/1049909119897241
[673] Armstrong MJ, Alliance S, Taylor A, Corsentino P, Galvin JE (2019) End-of-life experiences in dementia with Lewy bodies: Qualitative interviews with former caregivers. *Plos One* **14**. doi:10.1371/journal.pone.0217039
[674] Armstrong MJ, Gamez N, Alliance S, Majid T, Taylor A, Kurasz AM, Patel B, Smith G (2020) Research priorities of caregivers and individuals with dementia with Lewy bodies: An interview study. *Plos One* **15**. doi:10.1371/journal.pone.0239279
[675] Armstrong MJ, Gamez N, Alliance S, Majid T, Taylor AS, Kurasz AM, Patel B, Smith GE (2021) Clinical Care and Unmet Needs of Individuals With Dementia With Lewy Bodies and Caregivers An Interview Study. *Alzheimer Disease & Associated Disorders* **35**, 327-334. doi:10.1097/wad.0000000000000459
[676] Armstrong MJ, Paulson HL, Maixner SM, Fields JA, Lunde AM, Boeve BF, Manning C, Galvin JE, Taylor AS, Li ZG (2021) Protocol for an observational cohort study identifying factors predicting accurately end of life in dementia with Lewy bodies and promoting quality end-of-life experiences: the PACE-DLB study. *Bmj Open* **11**. doi:10.1136/bmjopen-2020-047554
[677] Arnulf I (2012) REM sleep behavior disorder: Motor manifestations and pathophysiology. *Movement Disorders* **27**, 677-689. doi:10.1002/mds.24957

[678] Attreed A, Morand LR, Pond DC, Sturmberg JP (2024) The Clinical Role of Heart Rate Variability Assessment in Cognitively Impaired Patients and Its Applicability in Community Care Settings: A Systematic Review of the Literature. *Cureus Journal of Medical Science* **16**. doi:10.7759/cureus.61703

[679] Au R, Seshadri S, Knox K, Beiser A, Himali JJ, Cabral HJ, Auerbach S, Green RC, Wolf PA, McKee AC (2012) The Framingham Brain Donation Program: Neuropathology Along the Cognitive Continuum. *Current Alzheimer Research* **9**, 673-686.

[680] Ballard C, Patel A, Oyebode F, Wilcock G (1996) Cognitive decline in patients with Alzheimer's disease, vascular dementia and senile dementia of Lewy body type. *Age and Ageing* **25**, 209-213. doi:10.1093/ageing/25.3.209

[681] Beekman ATF (2011) Neuropathological correlates of late-life depression. *Expert Review of Neurotherapeutics* **11**, 947-949. doi:10.1586/ern.11.88

[682] Beretta L, Caminiti SP, Santangelo R, Magnani G, Ferrari-Pellegrini F, Caffarra P, Perani D (2019) Two distinct pathological substrates associated with MMSE-pentagons item deficit in DLB and AD. *Neuropsychologia* **133**. doi:10.1016/j.neuropsychologia.2019.107174

[683] Bertrand E, Lechowicz W, Szpak GM, Dymecki J (1997) Qualitative and quantitative analysis of locus coeruleus neurons in Parkinson's disease. *Folia Neuropathologica* **35**, 80-86.

[684] Blanke O, Bernasconi F, Potheegadoo J (2023) Phantom Boarder Relates to Experimentally-Induced Presence Hallucinations in Parkinson's Disease. *Movement Disorders Clinical Practice* **10**, 617-624. doi:10.1002/mdc3.13684

[685] Bombois S, Derambure P, Pasquier F, Monaca C (2010) Sleep disorders in aging and dementia. *Journal of Nutrition Health & Aging* **14**, 212-217. doi:10.1007/s12603-010-0052-7

[686] Bradfield NI (2023) Mild Cognitive Impairment: Diagnosis and Subtypes. *Clinical Eeg and Neuroscience* **54**, 4-11. doi:10.1177/15500594211042708

[687] Bradshaw J, Saling M, Hopwood M, Anderson V, Brodtmann A (2004) Fluctuating cognition in dementia with Lewy bodies and Alzheimer's disease is qualitatively distinct. *Journal of Neurology Neurosurgery and Psychiatry* **75**, 382-387. doi:10.1136/jnnp.2002.002576

[688] Bramich S, King A, Kuruvilla M, Naismith SL, Noyce A, Alty J (2022) Isolated REM sleep behaviour disorder: current diagnostic procedures and emerging new technologies. *Journal of Neurology* **269**, 4684-4695. doi:10.1007/s00415-022-11213-9

[689] Bräuer S, Rossi M, Sajapin J, Henle T, Gasser T, Parchi P, Brockmann K, Falkenburger BH (2023) Kinetic parameters of alpha-synuclein seed amplification assay correlate with cognitive impairment in patients with Lewy body disorders. *Acta Neuropathologica Communications* **11**. doi:10.1186/s40478-023-01653-3

[690] Brayne C, Richardson K, Matthews FE, Fleming J, Hunter S, Xuereb JH, Paykel E, Mukaetova-Ladinska EB, Huppert FA, O'Sullivan A, Dening T, Neuropathology CCS (2009) Neuropathological Correlates of Dementia in Over-80-Year-Old Brain Donors from the Population-Based Cambridge City over-75s Cohort (CC75C) Study. *Journal of Alzheimers Disease* **18**, 645-658. doi:10.3233/jad-2009-1182

[691] Brockhuis B, Slawek J, Wieczorek D, Ussorowska D, Derejko M, Romanowicz G, Marks W, Dubaniewicz M (2006) Cerebral blood flow changes in patients with dementia with Lewy Bodies (DLB). A study of 6 cases. *Nuclear Medicine Review* **9**, 114-118.
[692] Brown LJE, Aldridge Z, Pepper A, Leroi I, Dening KH (2022) 'It's just incredible the difference it has made': family carers' experiences of a specialist Lewy body dementia Admiral Nurse service. *Age and Ageing* **51**. doi:10.1093/ageing/afac207
[693] Bukhatwa S, Zeng BY, Rose S, Jenner P (2010) A comparison of changes in proteasomal subunit expression in the substantia nigra in Parkinson's disease, multiple system atrophy and progressive supranuclear palsy. *Brain Research* **1326**, 174-183. doi:10.1016/j.brainres.2010.02.045
[694] Burkhard PR, Sanchez JC, Landis T, Hochstrasser DF (2001) CSF detection of the 14-3-3 protein in unselected patients with dementia. *Neurology* **56**, 1528-1533. doi:10.1212/wnl.56.11.1528
[695] Caffarra P, Gardini S, Dieci F, Copelli S, Maset L, Concari L, Farina E, Grossi E (2013) The qualitative scoring MMSE pentagon test (QSPT): A new method for differentiating dementia with Lewy Body from Alzheimer's disease. *Behavioural Neurology* **27**, 213-220. doi:10.1155/2013/728158
[696] Cagnin A, Bussè C, Jelcic N, Gnoato F, Mitolo M, Caffarra P (2015) High specificity of MMSE pentagon scoring for diagnosis of prodromal dementia with Lewy bodies. *Parkinsonism & Related Disorders* **21**, 303-305. doi:10.1016/j.parkreldis.2014.12.007
[697] Cagnin A, Busssè C, Gardini S, Jelcic N, Guzzo C, Gnoato F, Mitolo M, Ermani M, Caffarra P (2015) Clinical and Cognitive Phenotype of Mild Cognitive Impairment Evolving to Dementia with Lewy Bodies. *Dementia and Geriatric Cognitive Disorders Extra* **5**, 442-449. doi:10.1159/000441184
[698] Cagnin A, Di Lorenzo R, Marra C, Bonanni L, Cupidi C, Laganà V, Rubino E, Vacca A, Provero P, Isella V, Vanacore N, Agosta F, Appollonio I, Caffarra P, Pettenuzzo I, Sambati R, Quaranta D, Guglielmi V, Logroscino G, Filippi M, Tedeschi G, Ferrarese C, Rainero I, Bruni AC, Grp SICS (2020) Behavioral and Psychological Effects of Coronavirus Disease-19 Quarantine in Patients With Dementia. *Frontiers in Psychiatry* **11**. doi:10.3389/fpsyt.2020.578015
[699] Carli G, Caminiti SP, Galbiati A, Marelli S, Casoni F, Padovani A, Ferini-Strambi L, Perani D (2020) <i>In-vivo</i>signatures of neurodegeneration in isolated rapid eye movement sleep behaviour disorder. *European Journal of Neurology* **27**, 1285-1295. doi:10.1111/ene.14215
[700] Chanpalay V, Asan E (1989) ALTERATIONS IN CATECHOLAMINE NEURONS OF THE LOCUS COERULEUS IN SENILE DEMENTIA OF THE ALZHEIMER TYPE AND IN PARKINSONS-DISEASE WITH AND WITHOUT DEMENTIA AND DEPRESSION. *Journal of Comparative Neurology* **287**, 373-392. doi:10.1002/cne.902870308
[701] Chanthamontri C, Liu J, McLuckey SA (2009) Charge state dependent fragmentation of gaseous α-synuclein cations via ion trap and beam-type collisional activation. *International Journal of Mass Spectrometry* **283**, 9-16. doi:10.1016/j.ijms.2008.12.007

[702] Chen G, Liu S, Wu H, Gan JH, Wang XD, Ji Y (2023) Analysis of clinical characteristics of mirror and TV signs in Alzheimer's disease and dementia with Lewy bodies. *Journal of International Medical Research* **51**. doi:10.1177/03000605231156098

[703] Chhabra A, Singh G, Waraich SS, Sidhu B, Kumar G (2006) in *Conference of the World-Academy-of-Science-Engineering-and-Technology*, Venice, ITALY, pp. 39-42.

[704] Coindreau V, Chesnel C, Babany F, Declemy A, Savard E, Charlanes A, Lebreton F, Amarenco G (2020) Urinary tract symptoms in Lewy body dementia: About 19 cases. *Progres En Urologie* **30**, 267-272. doi:10.1016/j.purol.2020.02.007

[705] Collía A, Iranzo A, Serradell M, Muñoz-Lopetegi A, Mayà G, Santamaría J, Sánchez-Valle R, Gaig C (2023) Former participation in professional football as an occupation in patients with isolated REM sleep behavior disorder leading to a synucleinopathy: a case-control study. *Journal of Neurology* **270**, 3234-3242. doi:10.1007/s00415-023-11591-8

[706] Cummings J, Emre M, Aarsland D, Tekin S, Dronamraju N, Lane R (2010) Effects of Rivastigmine in Alzheimer's Disease Patients with and Without Hallucinations. *Journal of Alzheimers Disease* **20**, 301-311. doi:10.3233/jad-2010-1362

[707] D'Antonio F, Boccia M, Di Vita A, Suppa A, Fabbrini A, Canevelli M, Caramia F, Fiorelli M, Guariglia C, Ferracuti S, De Lena C, Aarsland D, Ffytche D (2022) Visual hallucinations in Lewy body disease: pathophysiological insights from phenomenology. *Journal of Neurology* **269**, 3636-3652. doi:10.1007/s00415-022-10983-6

[708] de Silva HA, Gunatilake SB, Smith AD (2003) Prevalence of dementia in a semi-urban population in Sri Lanka: report from a regional survey. *International Journal of Geriatric Psychiatry* **18**, 711-715. doi:10.1002/gps.909

[709] Denson MA, Wszolek ZK, Pfeiffer RF, Wszolek EK, Paschall TM, McComb RD (1997) Familial Parkinsonism, dementia, and Lewy body disease: Study of Family G. *Annals of Neurology* **42**, 638-643. doi:10.1002/ana.410420415

[710] Desmarais P, Massoud F, Filion J, Nguyen QD, Bajsarowicz P (2016) Quetiapine for Psychosis in Parkinson Disease and Neurodegenerative Parkinsonian Disorders: A Systematic Review. *Journal of Geriatric Psychiatry and Neurology* **29**, 227-236. doi:10.1177/0891988716640378

[711] Donaghy PC, Barnett N, Olsen K, Taylor JP, McKeith IG, O'Brien JT, Thomas AJ (2017) Symptoms associated with Lewy body disease in mild cognitive impairment. *International Journal of Geriatric Psychiatry* **32**, 1163-1171. doi:10.1002/gps.4742

[712] Donnemiller E, Heilmann J, Wenning GK, Berger W, Decristoforo C, Moncayo R, Poewe W, Ransmayr G (1997) Brain perfusion scintigraphy with Tc-99m-HMPAO or Tc-99m-ECD and I-123-beta-CIT single-photon emission tomography in dementia of the Alzheimer-type and diffuse Lewy body disease. *European Journal of Nuclear Medicine* **24**, 320-325.

[713] Doubleday EK, Snowden JS, Varma AR, Neary D (2002) Qualitative performance characteristics differentiate dementia with Lewy bodies and Alzheimer's disease. *Journal of Neurology Neurosurgery and Psychiatry* **72**, 602-607. doi:10.1136/jnnp.72.5.602

[714] Duro D, Tábuas-Pereira M, Freitas S, Santiago B, Botelho MA, Santana I (2018) Validity and clinical utility of different clock drawing test scoring systems in multiple forms of dementia. *Journal of Geriatric Psychiatry and Neurology* **31**, 114-122. doi:10.1177/0891988718774432

[715] Dymecki J, Lechowicz W, Bertrand E, Szpak GM (1996) Changes in dopaminergic neurons of the mesocorticolimbic system in Parkinson's disease. *Folia Neuropathologica* **34**, 102-106.

[716] Eversfield CL, Orton LD (2019) Auditory and visual hallucination prevalence in Parkinson's disease and dementia with Lewy bodies: a systematic review and meta-analysis. *Psychological Medicine* **49**, 2342-2353. doi:10.1017/S0033291718003161

[717] Falque A, Jordanis M, Landré L, de Sousa PL, Mondino M, Furcieri E, Blanc F (2022) Neural basis of impaired narrative discourse comprehension in prodromal and mild dementia with lewy bodies. *Frontiers in Aging Neuroscience* **14**. doi:10.3389/fnagi.2022.939973

[718] Farfel JM, Nitrini R, Suemoto CK, Grinberg LT, Ferretti REL, Leite REP, Tampellini E, Lima L, Farias DS, Neves RC, Rodriguez RD, Menezes PR, Fregni F, Bennett DA, Pasqualucci CA, Jacob W, Brazilian Aging Brain Study G (2013) Very low levels of education and cognitive reserve A clinicopathologic study. *Neurology* **81**, 650-657. doi:10.1212/WNL.0b013e3182a08f1b

[719] Fedorova TD, Knudsen K, Sommerauer M, Svendsen KB, Otto M, Borghammer P (2020) A Screening-Based Method for Identifying Patients with REM Sleep Behaviour Disorder in a Danish Community Setting. *Journal of Parkinsons Disease* **10**, 1249-1253. doi:10.3233/jpd-202020

[720] Fernandez-Gomez FJ, Jumeau F, Derisbourg M, Burnouf S, Tran H, Eddarkaoui S, Obriot H, Dutoit-Lefevre V, Deramecourt V, Mitchell V, Lefranc D, Hamdane M, Blum D, Buée L, Buée-Scherrer V, Sergeant N (2014) Consensus Brain-derived Protein, Extraction Protocol for the Study of Human and Murine Brain Proteome Using Both 2D-DIGE and Mini 2DE Immunoblotting. *Jove-Journal of Visualized Experiments*. doi:10.3791/51339

[721] Foy CML, Nicholas H, Hollingworth P, Boothby H, Willams J, Brown RG, Al-Sarraj S, Lovestone S (2007) Diagnosing Alzheimer's disease - non-clinicians and computerised algorithms together are as accurate as the best clinical practice. *International Journal of Geriatric Psychiatry* **22**, 1154-1163. doi:10.1002/gps.1810

[722] Gabb VG, Blackman J, Morrison HD, Biswas B, Li HX, Turner N, Russell GM, Greenwood R, Jolly A, Trender W, Hampshire A, Whone A, Coulthard E (2024) Remote Evaluation of Sleep and Circadian Rhythms in Older Adults With Mild Cognitive Impairment and Dementia: Protocol for a Feasibility and Acceptability Mixed Methods Study. *Jmir Research Protocols* **13**. doi:10.2196/52652

[723] Gallop K, Pham N, Maclaine G, Saunders E, Black B, Acaster S (2023) Exploring the impact of caring for an individual with neurogenic orthostatic hypotension: a qualitative study. *Neurodegenerative Disease Management* **13**, 191-201. doi:10.2217/nmt-2022-0016

[724] Galvin JE, Malcom H, Johnson D, Morris JC (2007) Personality traits distinguishing dementia with Lewy bodies from Alzheimer disease. *Neurology* **68**, 1895-1901. doi:10.1212/01.wnl.0000263131.80945.ad

[725] Gibbons LE, Power MC, Walker RL, Kumar RG, Murphy A, Latimer CS, Nolan AL, Melief EJ, Beller A, Bogdani M, Keene CD, Larson EB, Crane PK, Dams-O'Connor K (2023) Association of Traumatic Brain Injury with Late Life Neuropathological Outcomes in a Community-Based Cohort. *Journal of Alzheimers Disease* **93**, 949-961. doi:10.3233/jad-221224

[726] Gibson LL, Grinberg LT, Ffytche D, Leite REP, Rodriguez RD, Ferretti-Rebustini REL, Pasqualucci CA, Nitrini R, Jacob-Filho W, Aarsland D, Suemoto CK (2023) Neuropathological correlates of neuropsychiatric symptoms in dementia. *Alzheimers & Dementia* **19**, 1372-1382. doi:10.1002/alz.12765

[727] Goetz CG, Vogel C, Tanner CM, Stebbins GT (1998) Early dopaminergic drug-induced hallucinations in parkinsonian patients. *Neurology* **51**, 811-814. doi:10.1212/wnl.51.3.811

[728] Gossard TR, Teigen LN, Yoo S, Timm PC, Jagielski J, Bibi N, Feemster JC, Steele T, Carvalho DZ, Junna MR, Lipford MC, Peikert MT, LeClair-Visonneau L, McCarter SJ, Boeve BF, Silber MH, Hirsch J, Sharp RR, St Louis EK (2023) Patient values and preferences regarding prognostic counseling in isolated REM sleep behavior disorder. *Sleep* **46**. doi:10.1093/sleep/zsac244

[729] Guo X, Meng QB, Zuo MM, Su Z, Gan JH, Li XD, Zhu HC, Gang BZ, Niu JP, Liu S, Ji Y (2023) Costs of dementia with lewy bodies: A Chinese multicenter cross-sectional study. *International Journal of Geriatric Psychiatry* **38**. doi:10.1002/gps.5848

[730] Harciarek M, Kertesz A (2008) The prevalence of misidentification syndromes in neurodegenerative diseases. *Alzheimer Disease & Associated Disorders* **22**, 163-169. doi:10.1097/WAD.0b013e3181641341

[731] Harding E, Rossi-Harries S, Gerritzen EV, Zimmerman N, Hoare Z, Proctor D, Brotherhood E, Crutch S, Suarez-González A (2023) "I felt like I had been put on the shelf and forgotten about" - lasting lessons about the impact of COVID-19 on people affected by rarer dementias. *Bmc Geriatrics* **23**. doi:10.1186/s12877-023-03992-1

[732] Hartikainen S, Rahkonen T, Kautiainen H, Sulkava R (2003) Use of psychotropics among home-dwelling nondemented and demented elderly. *International Journal of Geriatric Psychiatry* **18**, 1135-1141. doi:10.1002/gps.1024

[733] Hashimoto M, Sakamoto S, Ikeda M (2015) Clinical Features of Delusional Jealousy in Elderly Patients With Dementia. *Journal of Clinical Psychiatry* **76**, 691-695. doi:10.4088/JCP.14m09018

[734] Heyman I, Brorsson A, Persson T, Londos E (2023) Pacemaker Implants and Their Influence on the Daily Life of Patients with Dementia with Lewy Bodies: A Qualitative Case Study. *Neurology and Therapy* **12**, 1359-1373. doi:10.1007/s40120-023-00513-5

[735] Hindle JV, Watermeyer TJ, Roberts J, Martyr A, Lloyd-Williams H, Brand A, Gutting P, Hoare Z, Edwards RT, Clare L (2016) Cognitive rehabiliation for Parkinson's disease demantia: a study protocol for a pilot randomised controlled trial. *Trials* **17**. doi:10.1186/s13063-016-1253-0

[736] Honig LS, Chambliss DD, Bigio EH, Carroll SL, Elliott JL (2000) Glutamate transporter EAAT2 splice variants occur not only in ALS, but also in AD and controls. *Neurology* **55**, 1082-1088. doi:10.1212/wnl.55.8.1082
[737] Huang WC, Chang MC, Wang WF, Jhang KM (2022) A Comparison of Caregiver Burden for Different Types of Dementia: An 18-Month Retrospective Cohort Study. *Frontiers in Psychology* **12**. doi:10.3389/fpsyg.2021.798315
[738] Iaccarino L, Marelli S, Iannaccone S, Magnani G, Ferini-Strambi L, Perani D (2016) Severe Brain Metabolic Decreases Associated with REM Sleep Behavior Disorder in Dementia with Lewy Bodies. *Journal of Alzheimers Disease* **52**, 989-997. doi:10.3233/jad-151000
[739] Ikeda M, Mori E, Kosaka K, Iseki E, Hashimoto M, Matsukawa N, Matsuo K, Nakagawa M, Donepezil DLBSI (2013) Long-Term Safety and Efficacy of Donepezil in Patients with Dementia with Lewy Bodies: Results from a 52-Week, Open-Label, Multicenter Extension Study. *Dementia and Geriatric Cognitive Disorders* **36**, 229-241. doi:10.1159/000351672
[740] Ikeda M, Mori E, Orimo S, Yamada T, Konishi O (2023) Efficacy of Adjunctive Therapy with Zonisamide Versus Increased Dose of Levodopa for Motor Symptoms in Patients with Dementia with Lewy Bodies: The Randomized, Controlled, Non-Inferiority DUEL Study. *Journal of Alzheimers Disease* **95**, 251-264. doi:10.3233/jad-230335
[741] Ince PG, Perry EK, Morris CM (1998) Dementia with Lewy bodies. A distinct non-Alzheimer dementia syndrome? *Brain Pathology* **8**, 299-324.
[742] Ishimaru D, Kanemoto H, Hotta M, Nagata Y, Koizumi F, Satake Y, Taomoto D, Ikeda M (2024) Case report: Environmental adjustment for visual hallucinations in dementia with Lewy bodies based on photo assessment of the living environment. *Frontiers in Psychiatry* **15**. doi:10.3389/fpsyt.2024.1283156
[743] Iwasaki K, Kosaka K, Mori H, Okitsu R, Furukawa K, Manabe Y, Yoshita M, Kanamori A, Ito N, Wada K, Kitayama M, Horiguchi J, Yamaguchi S, Takayama S, Fukuhara R, Ouma S, Nakano S, Hashimoto M, Kinoshita T (2012) Improvement in delusions and hallucinations in patients with dementia with Lewy bodies upon administration of yokukansan, a traditional Japanese medicine. *Psychogeriatrics* **12**, 235-241. doi:10.1111/j.1479-8301.2012.00413.x
[744] Jacquet AD, Timmers M, Ma SY, Thieme A, McCabe GP, Vest JHC, Lila MA, Rochet JC (2017) Lumbee traditional medicine: Neuroprotective activities of medicinal plants used to treat Parkinson's disease-related symptoms. *Journal of Ethnopharmacology* **206**, 408-425. doi:10.1016/j.jep.2017.02.021
[745] Jhoo JH, Kim KW, Huh Y, Lee SB, Park JH, Lee JJ, Choi EA, Han C, Choo IH, Youn JC, Lee DY, Woo JI (2008) Prevalence of dementia and its subtypes in an elderly urban Korean population: Results from the Korean Longitudinal Study on Health and Aging (KLoSHA). *Dementia and Geriatric Cognitive Disorders* **26**, 270-276. doi:10.1159/000160960
[746] Jreige M, Kurian GK, Perriraz J, Potheegadoo J, Bernasconi F, Stampacchia S, Blanke O, Alessandra G, Lejay N, Chiabotti PS, Rouaud O, Lalonde MN, Schaefer N, Treglia G, Allali G, Prior JO (2023) The diagnostic performance of functional dopaminergic

scintigraphic imaging in the diagnosis of dementia with Lewy bodies: an updated systematic review. *European Journal of Nuclear Medicine and Molecular Imaging* **50**, 1988-2035. doi:10.1007/s00259-023-06154-y
[747] Kanemoto H, Sato S, Satake Y, Koizumi F, Taomoto D, Kanda A, Wada T, Yoshiyama K, Ikeda M (2021) Impact of Behavioral and Psychological Symptoms on Caregiver Burden in Patients With Dementia With Lewy Bodies. *Frontiers in Psychiatry* **12**. doi:10.3389/fpsyt.2021.753864
[748] Kashihara K, Ohno M, Kawada S, Imamura T (2008) Frequent nocturnal vocalization in pure autonomic failure. *Journal of International Medical Research* **36**, 489-495. doi:10.1177/147323000803600313
[749] Kiesmann M, Sauleau E, Perisse J, Jehl C, Konrad S, Karcher P, Fleury MC, Rohmer D, Sauer A, Ehret M, Vogel T, Kaltenbach G, Schmitt E (2021) Parkinsonian gait in elderly people: Significance of the threshold value of two and more falls per year. *Revue Neurologique* **177**, 385-393. doi:10.1016/j.neurol.2020.06.012
[750] Killen A, Flynn D, O'Brien N, Taylor JP (2022) The feasibility and acceptability of a psychosocial intervention to support people with dementia with Lewy bodies and family care partners. *Dementia-International Journal of Social Research and Practice* **21**, 77-93. doi:10.1177/14713012211028501
[751] Kim HJ, Choi KH, Kim SH, Cummings JL, Yang DW (2016) Validation Study of the Korean Version of the Brief Clinical Form of the Neuropsychiatric Inventory. *Dementia and Geriatric Cognitive Disorders Extra* **6**, 214-221. doi:10.1159/000445828
[752] Kindell J, Keady J, Sage K, Wilkinson R (2017) Everyday conversation in dementia: a review of the literature to inform research and practice. *International Journal of Language & Communication Disorders* **52**, 392-406. doi:10.1111/1460-6984.12298
[753] Kirk A (2007) Target symptoms and outcome measures: Cognition. *Canadian Journal of Neurological Sciences* **34**, S42-S46. doi:10.1017/s0317167100005552
[754] Larsson V, Holmbom-Larsen A, Torisson G, Strandberg EL, Londos E (2019) Living with dementia with Lewy bodies: an interpretative phenomenological analysis. *Bmj Open* **9**. doi:10.1136/bmjopen-2018-024983
[755] Law ZK, Todd C, Mehraram R, Schumacher J, Baker MR, LeBeau FEN, Yarnall A, Onofrj M, Bonanni L, Thomas A, Taylor JP (2020) The Role of EEG in the Diagnosis, Prognosis and Clinical Correlations of Dementia with Lewy Bodies-A Systematic Review. *Diagnostics* **10**. doi:10.3390/diagnostics10090616
[756] Lee DR, McKeith I, Mosimann U, Ghosh-Nodyal A, Thomas AJ (2013) Examining carer stress in dementia: the role of subtype diagnosis and neuropsychiatric symptoms. *International Journal of Geriatric Psychiatry* **28**, 135-141. doi:10.1002/gps.3799
[757] Lee H, Brekelmans GJF, Roks G (2015) The EEG as a diagnostic tool in distinguishing between dementia with Lewy bodies and Alzheimer's disease. *Clinical Neurophysiology* **126**, 1735-1739. doi:10.1016/j.clinph.2014.11.021
[758] Leroi I, Vatter S, Carter LA, Smith SJ, Orgeta V, Poliakoff E, Silverdale MA, Raw J, Ahearn DJ, Taylor C, Rodda J, Abdel-Ghany T, McCormick SA (2019) Parkinson's-adapted cognitive stimulation therapy: a pilot randomized controlled clinical trial.

Therapeutic Advances in Neurological Disorders **12**. doi:10.1177/1756286419852217
[759] Leu-Semenescu S, Arnulf I (2010) Disruptive nocturnal behavior in elderly subjects: could it be a parasomnia? *Psychologie & Neuropsychiatrie Du Vieillissement* **8**, 97-109. doi:10.1684/pnv.2010.0210
[760] Levy JA, Chelune GJ (2007) Cognitive-behavioral profiles of Neurodegenerative dementias: Beyond Alzheimer's disease. *Journal of Geriatric Psychiatry and Neurology* **20**, 227-238. doi:10.1177/0891988707308806
[761] Lim SY, Closas A, Tan AH, Lim JL, Tan YJ, Vijayanathan Y, Tay YW, Khalid RBA, Ng WK, Kanesalingam R, Martin PM, Annuar AA, Lit LC, Foo JN, Lim WK, Ng ASL, Tan EK (2023) New insights from a multi-ethnic Asian progressive supranuclear palsy cohort. *Parkinsonism & Related Disorders* **108**. doi:10.1016/j.parkreldis.2023.105296
[762] Lin W, Xie YC, Cheng PY, Dong LY, Hung GU, Chiu PY (2018) Association of visual hallucinations with very mild degenerative dementia due to dementia with Lewy bodies. *Plos One* **13**. doi:10.1371/journal.pone.0205909
[763] Lindeberg S, Müller N, Samuelsson C (2023) Conversations in dementia with Lewy bodies: Resources and barriers in communication. *International Journal of Language & Communication Disorders* **58**, 419-432. doi:10.1111/1460-6984.12799
[764] Liu S, Liu J, Wang XD, Shi ZH, Zhou YY, Li J, Yu T, Ji Y (2018) Caregiver burden, sleep quality, depression, and anxiety in dementia caregivers: a comparison of frontotemporal lobar degeneration, dementia with Lewy bodies, and Alzheimer's disease. *International Psychogeriatrics* **30**, 1131-1138. doi:10.1017/s1041610217002630
[765] Lomax CL, Brown RG, Howard RJ (2004) Measuring disability in patients with neurodegenerative disease using the 'Yesterday Interview'. *International Journal of Geriatric Psychiatry* **19**, 1058-1064. doi:10.1002/gps.1210
[766] López-Pousa S, Garre-Olmo J, Turon-Estrada A, Gelada-Badle E, Lozano-Gallego M, Hernández-Ferrándiz M, Morante-Muñoz V, Peralta-Rodríguez J, Cruz-Reina MM (2003) Clinical incidence of Dementia with Lewy Bodies. *Revista De Neurologia* **36**, 715-720. doi:10.33588/rn.3608.2002580
[767] Maclin JMA, Wang T, Xiao SF (2019) Biomarkers for the diagnosis of Alzheimer's disease, dementia Lewy body, frontotemporal dementia and vascular dementia. *General Psychiatry* **32**. doi:10.1136/gpsych-2019-100054
[768] Mahajan A, Bulica B, Ahmad A, Kaminski P, LeWitt P, Taylor D, Krstevska S, Patel N (2018) Pimavanserin use in a movement disorders clinic: a single-center experience. *Neurological Sciences* **39**, 1767-1771. doi:10.1007/s10072-018-3500-5
[769] McCormick SA, McDonald KR, Vatter S, Orgeta V, Poliakoff E, Smith S, Silverdale MA, Fu B, Leroi I (2017) Psychosocial therapy for Parkinson's-related dementia: study protocol for the INVEST randomised controlled trial. *Bmj Open* **7**. doi:10.1136/bmjopen-2017-016801
[770] McIntyre A, Harding E, Yong KXX, Sullivan MP, Gilhooly M, Gilhooly K, Woodbridge R, Crutch S (2019) Health and social care practitioners' understanding of the problems

of people with dementia-related visual processing impairment. *Health & Social Care in the Community* **27**, 982-990. doi:10.1111/hsc.12715
[771] McShane R, Keene J, Gedling K, Fairburn C, Jacoby R, Hope T (1997) Do neuroleptic drugs hasten cognitive decline in dementia? Prospective study with necropsy follow up. *Bmj-British Medical Journal* **314**, 266-270. doi:10.1136/bmj.314.7076.266
[772] Miki Y, Tanji K, Mori F, Utsumi J, Sasaki H, Kakita A, Takahashi H, Wakabayashi K (2016) Alteration of Upstream Autophagy-Related Proteins (ULK1, ULK2, Beclin1, VPS34 and AMBRA1) in Lewy Body Disease. *Brain Pathology* **26**, 359-370. doi:10.1111/bpa.12297
[773] Mitolo M, Salmon DP, Gardini S, Galasko D, Grossi E, Caffarra P (2014) The New Qualitative Scoring MMSE Pentagon Test (QSPT) as a Valid Screening Tool between Autopsy-Confirmed Dementia with Lewy Bodies and Alzheimer's Disease. *Journal of Alzheimers Disease* **39**, 823-832. doi:10.3233/jad-131403
[774] Mori E, Ikeda M, Kosaka K, Donepezil DLBSI (2012) Donepezil for Dementia with Lewy Bodies: A Randomized, Placebo-Controlled Trial. *Annals of Neurology* **72**, 41-52. doi:10.1002/ana.23557
[775] Mosimann UP, Rowan EN, Partington CE, Collerton D, Littlewood E, O'Brien JT, Burn DJ, McKeith IG (2006) Characteristics of visual hallucinations in Parkinson disease dementia and dementia with Lewy bodies. *American Journal of Geriatric Psychiatry* **14**, 153-160. doi:10.1097/01.Jgp.0000192480.89813.80
[776] Murayama N, Masubuchi Y, Kimura A, Uchiyama K, Yamagata M, Ota K, Iseki E (2023) A simple method to evaluate the pentagon copy test of the Mini-Mental State Examination for the differentiation of dementia with Lewy bodies. *Applied Neuropsychology-Adult*. doi:10.1080/23279095.2023.2200948
[777] Nwabuobi L, Barbosa W, Sweeney M, Oyler S, Meisel T, Di Rocco A, Chodosh J, Fleisher JE (2019) Sex-related differences in homebound advanced Parkinson's disease patients. *Clinical Interventions in Aging* **14**, 1371-1377. doi:10.2147/cia.S203690
[778] Oliveira FPM, Walker Z, Walker RWH, Attems J, Castanheira JC, Silva A, Oliveira C, Vaz S, Silva M, Costa DC (2021) ¹²³I-FP-CIT SPECT in dementia with Lewy bodies, Parkinson's disease and Alzheimer's disease: a new quantitative analysis of autopsy confirmed cases. *Journal of Neurology Neurosurgery and Psychiatry* **92**, 662-667. doi:10.1136/jnnp-2020-324606
[779] Onofrj M, Bonanni L, Manzoli L, Thomas A (2010) Cohort study on somatoform disorders in Parkinson disease and dementia with Lewy bodies. *Neurology* **74**, 1598-1606. doi:10.1212/WNL.0b013e3181df09dd
[780] Onofrj M, Thomas A, Tiraboschi P, Wenning G, Gambi F, Sepede G, Di Giannantonio M, Di Carmine C, Monaco D, Maruotti V, Ciccocioppo F, D'Amico MC, Bonanni L (2011) Updates on Somatoform Disorders (SFMD) in Parkinson's Disease and Dementia with Lewy Bodies and discussion of phenomenology. *Journal of the Neurological Sciences* **310**, 166-171. doi:10.1016/j.jns.2011.07.010
[781] Onofrj M, Varanese S, Bonanni L, Taylor JP, Antonini A, Valente EM, Petrucci S, Stocchi F, Thomas A, Perfetti B (2013) Cohort study of prevalence and

phenomenology of tremor in dementia with Lewy bodies. *Journal of Neurology* **260**, 1731-1742. doi:10.1007/s00415-013-6853-y
[782] Oudiette D, De Cock VC, Lavault S, Leu S, Vidailhet M, Arnulf I (2009) Nonviolent elaborate behaviors may also occur in REM sleep behavior disorder. *Neurology* **72**, 551-557. doi:10.1212/01.wnl.0000341936.78678.3a
[783] Pagonabarraga J, Llebaria G, García-Sánchez C, Pascual-Sedano B, Gironell A, Kulisevsky J (2008) A prospective study of delusional misidentification syndromes in Parkinson's disease with dementia. *Movement Disorders* **23**, 443-448. doi:10.1002/mds.21864
[784] Park J, Howard H, Tolea MI, Galvin JE (2020) Perceived Benefits of Using Nonpharmacological Interventions in Older Adults With Alzheimer's Disease or Dementia With Lewy Bodies. *Journal of Gerontological Nursing* **46**, 37-+. doi:10.3928/00989134-20191217-01
[785] Park JY, Lansbury PT (2003) β-synuclein inhibits formation of α-synuclein protofibrils:: A possible therapeutic strategy against Parkinson's disease. *Biochemistry* **42**, 3696-3700. doi:10.1021/bi020604a
[786] Perri R, Fadda L, Caltagirone C, Carlesimo GA (2013) Word List and Story Recall Elicit Different Patterns of Memory Deficit in Patients with Alzheimer's Disease, Frontotemporal Dementia, Subcortical Ischemic Vascular Disease, and Lewy Body Dementia. *Journal of Alzheimers Disease* **37**, 99-107. doi:10.3233/jad-130347
[787] Perry EK, Marshall E, Thompson P, McKeith IG, Collerton D, Fairbairn AF, Ferrier IN, Irving D, Perry RH (1993) MONOAMINERGIC ACTIVITIES IN LEWY-BODY-DEMENTIA - RELATION TO HALLUCINOSIS AND EXTRAPYRAMIDAL FEATURES. *Journal of Neural Transmission-Parkinsons Disease and Dementia Section* **6**, 167-177. doi:10.1007/bf02260919
[788] Piersma D, Fuermaier ABM, De Waard D, Davidse RJ, De Groot J, Doumen MJA, Bredewoud RA, Claesen R, Lemstra AW, Scheltens P, Vermeeren A, Ponds R, Verhey F, De Deyn PP, Brouwer WH, Tucha O (2018) Assessing Fitness to Drive in Patients With Different Types of Dementia. *Alzheimer Disease & Associated Disorders* **32**, 70-75. doi:10.1097/wad.0000000000000221
[789] Piscopo P, Marcon G, Piras MR, Crestini A, Campeggi LM, Deiana E, Cherchi R, Tanda F, Deplano A, Vanacore N, Tagliavini F, Pocchiari M, Giaccone G, Confaloni A (2008) A novel <i>PSEN2</i> mutation associated with a peculiar phenotype. *Neurology* **70**, 1549-1554. doi:10.1212/01.wnl.0000310643.53587.87
[790] Prentice JL, Schaeffer MJ, Wall AK, Callahan BL (2021) A systematic review and comparison of neurocognitive features of late-life attention-deficit/hyperactivity disorder and dementia with Lewy bodies. *Journal of Geriatric Psychiatry and Neurology* **34**, 466-481. doi:10.1177/0891988720944251
[791] Pullen AH, Martin JE (1995) ULTRASTRUCTURAL ABNORMALITIES WITH INCLUSIONS IN ONUFS NUCLEUS IN MOTOR-NEURON DISEASE (AMYOTROPHIC-LATERAL-SCLEROSIS). *Neuropathology and Applied Neurobiology* **21**, 327-340. doi:10.1111/j.1365-2990.1995.tb01067.x
[792] Rahkonen T, Eloniemi-Sulkava U, Rissanen S, Vatanen A, Viramo P, Sulkava R (2003) Dementia with Lewy bodies according to the consensus criteria in a general

population aged 75 years or older. *Journal of Neurology Neurosurgery and Psychiatry* **74**, 720-724. doi:10.1136/jnnp.74.6.720
[793] Rahman MM, Mim SA, Islam MR, Parvez A, Islam F, Uddin MB, Rahaman MS, Shuvo PA, Ahmed M, Greig NH, Kamal MA (2022) Exploring the Recent Trends in Management of Dementia and Frailty: Focus on Diagnosis and Treatment. *Current Medicinal Chemistry* **29**, 5289-5314. doi:10.2174/0929867329666220408102051
[794] Rainero I, Bruni AC, Marra C, Cagnin A, Bonanni L, Cupidi C, Laganà V, Rubino E, Vacca A, Di Lorenzo R, Provero P, Isella V, Vanacore N, Agosta F, Appollonio I, Caffarra P, Bussè C, Sambati R, Quaranta D, Guglielmi V, Logroscino G, Filippi M, Tedeschi G, Ferrarese C, Grp SIC-S (2021) The Impact of COVID-19 Quarantine on Patients With Dementia and Family Caregivers: A Nation-Wide Survey. *Frontiers in Aging Neuroscience* **12**. doi:10.3389/fnagi.2020.625781
[795] Reynolds AD, Glanzer JG, Kadiu I, Ricardo-Dukelow M, Chaudhuri A, Ciborowski P, Cerny R, Gelman B, Thomas MP, Mosley RL, Gendelman HE (2008) Nitrated alpha-synuclein-activated microglial profiling for Parkinson's disease. *Journal of Neurochemistry* **104**, 1504-1525. doi:10.1111/j.1471-4159.2007.05087.x
[796] Rockwood K, Fay S, Hamilton L, Ross E, Moorhouse P (2014) Good days and bad days in dementia: a qualitative chart review of variable symptom expression. *International Psychogeriatrics* **26**, 1239-1246. doi:10.1017/S1041610214000222
[797] Rongve A, Boeve BF, Aarsland D (2010) Frequency and correlates of caregiver-reported sleep disturbances in a sample of persons with early dementia. *Journal of the American Geriatrics Society* **58**, 480-486. doi:10.1111/j.1532-5415.2010.02733.x
[798] Roque M, Salva A, Vellas B (2013) Malnutrition in community-dwelling adults with dementia (Nutrialz Trial). *Journal of Nutrition Health & Aging* **17**, 295-299. doi:10.1007/s12603-012-0401-9
[799] Sakurai K, Kaneda D, Uchida Y, Inui S, Bundo M, Akagi A, Nihashi T, Kimura Y, Kato T, Ito K, Ohashi W, Hashizume Y (2021) Can Medial Temporal Impairment Be an Imaging Red Flag for Neurodegeneration in Disproportionately Enlarged Subarachnoid Space Hydrocephalus? *Journal of Alzheimers Disease* **83**, 1199-1209. doi:10.3233/jad-210535
[800] Schrempf W, Katona I, Dogan I, Felbert VV, Wienecke M, Heller J, Maier A, Hermann A, Linse K, Brandt MD, Reichmann H, Schulz JB, Schiefer J, Oertel WH, Storch A, Weis J, Reetz K (2016) Reduced intraepidermal nerve fiber density in patients with REM sleep behavior disorder. *Parkinsonism & Related Disorders* **29**, 10-16. doi:10.1016/j.parkreldis.2016.06.003
[801] Shindo A, Ueda Y, Kuzuhara S, Kokubo Y (2014) Neuropsychological study of amyotrophic lateral sclerosis and parkinsonism-dementia complex in Kii peninsula, Japan. *BMC Neurology* **14**.
[802] Snowden JS, Thompson JC, Stopford CL, Richardson AMT, Gerhard A, Neary D, Mann DMA (2011) The clinical diagnosis of early-onset dementias: diagnostic accuracy and clinicopathological relationships. *Brain* **134**, 2478-2492. doi:10.1093/brain/awr189

[803] Soennesyn H, Oppedal K, Greve OJ, Fritze F, Auestad BH, Nore SP, Beyer MK, Aarsland D (2012) White Matter Hyperintensities and the Course of Depressive Symptoms in Elderly People with Mild Dementia. *Dementia and Geriatric Cognitive Disorders Extra* **2**, 97-111. doi:10.1159/000335497
[804] Stacy KE, Lambert J, Shatz R, Bakas T (2023) Development and Validation of the Lewy Body Disease Caregiver Activities Scale. *Journal of Nursing Measurement* **31**, 606-614. doi:10.1891/JNM-2021-0100
[805] Stacy KE, Perazzo J, Shatz R, Bakas T (2022) Needs and concerns of Lewy body disease family caregivers: A qualitative study. *Western Journal of Nursing Research* **44**, 227-238. doi:10.1177/01939459211050957
[806] Stevens T, Livingston G, Kitchen G, Manela M, Walker Z, Katona C (2002) Islington study of dementia subtypes in the community. *British Journal of Psychiatry* **180**, 270-276. doi:10.1192/bjp.180.3.270
[807] Suárez-González A, Serrano-Pozo A, Arroyo-Anlló EM, Franco-Macías E, Polo J, García-Solís D, Gil-Néciga E (2014) Utility of neuropsychiatric tools in the differential diagnosis of dementia with Lewy bodies and Alzheimer's disease: quantitative and qualitative findings. *International Psychogeriatrics* **26**, 453-461. doi:10.1017/s1041610213002068
[808] Tan LPL, Herrmann N, Mainland BJ, Shulman K (2015) Can clock drawing differentiate Alzheimer's disease from other dementias? *International Psychogeriatrics* **27**, 1649-1660. doi:10.1017/S1041610215000939
[809] Tanaka MT, Miki Y, Bettencourt C, Ozaki T, Tanji K, Mori F, Kakita A, Wakabayashi K (2022) Involvement of autophagic protein DEF8 in Lewy bodies. *Biochemical and Biophysical Research Communications* **623**, 170-175. doi:10.1016/j.bbrc.2022.07.069
[810] Tarolli CG, Zimmerman GA, Goldenthal S, Feldman B, Berk S, Siddiqi B, Kopil CM, Chowdhury S, Biglan KM, Dorsey ER, Adams JL (2020) Video research visits for atypical parkinsonian syndromes among Fox Trial Finder participants. *Neurology-Clinical Practice* **10**, 7-14. doi:10.1212/cpj.0000000000000680
[811] Tateno M, Kobayashi S, Shirasaka T, Furukawa Y, Fujii K, Morii H, Yasumura S, Utsumi K, Saito T (2008) Comparison of the usefulness of brain perfusion SPECT and MIBG myocardial scintigraphy for the diagnosis of dementia with Lewy bodies. *Dementia and Geriatric Cognitive Disorders* **26**, 453-457. doi:10.1159/000165918
[812] Terzaghi M, Arnaldi D, Rizzetti MC, Minafra B, Cremascoli R, Rustioni V, Zangaglia R, Pasotti C, Sinforiani E, Pacchetti C, Manni R (2013) Analysis of Video-Polysomnographic Sleep Findings in Dementia With Lewy Bodies. *Movement Disorders* **28**, 1416-1423. doi:10.1002/mds.25523
[813] Thaipisuttikul P, Chittaropas P, Wisajun P, Jullagate S (2018) Development and validation of a screening instrument for cognitive fluctuation in patients with neurocognitive disorder with Lewy bodies (NCDLB): the Mayo Fluctuations Scale-Thai version. *General Psychiatry* **31**, 8-14. doi:10.1136/gpsych-2018-000001
[814] Thaipisuttikul P, Lobach I, Zweig Y, Gurnani A, Galvin JE (2013) Capgras syndrome in Dementia with Lewy Bodies. *International Psychogeriatrics* **25**, 843-849. doi:10.1017/S1041610212002189

[815] Thomas AJ, Taylor JP, McKeith I, Bamford C, Burn D, Allan L, O'Brien J (2017) Development of assessment toolkits for improving the diagnosis of the Lewy body dementias: feasibility study within the DIAMOND Lewy study. *International Journal of Geriatric Psychiatry* **32**, 1280-1304. doi:10.1002/gps.4609
[816] Toepper M, Falkenstein M (2019) Driving fitness in different forms of dementia: An update. *Journal of the American Geriatrics Society* **67**, 2186-2192. doi:10.1111/jgs.16077
[817] Tsopelas C, Stewart R, Savva GM, Brayne C, Ince P, Thomas A, Matthews FE, Med Res Council Cognitive F (2011) Neuropathological correlates of late-life depression in older people. *British Journal of Psychiatry* **198**, 109-114. doi:10.1192/bjp.bp.110.078816
[818] van de Beek M, van Steenoven I, van der Zande JJ, Barkhof F, Teunissen CE, van der Flier WM, Lemstra AW (2020) Prodromal Dementia with Lewy Bodies: Clinical Characterization and Predictors of Progression. *Movement Disorders* **35**, 859-867. doi:10.1002/mds.27997
[819] van de Beek M, van Steenoven I, van der Zande JJ, Porcelijn I, Barkhof F, Stam CJ, Raijmakers P, Scheltens P, Teunissen CE, van der Flier WM, Lemstra AW (2021) Characterization of symptoms and determinants of disease burden in dementia with Lewy bodies: DEvELOP design and baseline results. *Alzheimers Research & Therapy* **13**. doi:10.1186/s13195-021-00792-w
[820] van Loenhoud AC, de Boer C, Wols K, Pijnenburg YA, Lemstra AW, Bouwman FH, Prins ND, Scheltens P, Ossenkoppele R, van der Flier WM (2019) High occurrence of transportation and logistics occupations among vascular dementia patients: an observational study. *Alzheimers Research & Therapy* **11**. doi:10.1186/s13195-019-0570-4
[821] Vatter S, McDonald KR, Stanmore E, Clare L, McCormick SA, Leroi I (2018) A qualitative study of female caregiving spouses' experiences of intimate relationships as cognition declines in Parkinson's disease. *Age and Ageing* **47**, 604-610. doi:10.1093/ageing/afy049
[822] Vatter S, McDonald KR, Stanmore E, McCormick SA, Clare L, Leroi I (2020) A brief psychometric and clinimetric evaluation of self-report burden and mental health measures completed by care partners of people with Parkinson's-related dementia. *International Psychogeriatrics* **32**, 875-880. doi:10.1017/s1041610220000605
[823] Vieregge P, Hagenah J, Heberlein I, Klein C, Ludin HP (1999) Parkinson's disease in twins - A follow-up study. *Neurology* **53**, 566-572. doi:10.1212/wnl.53.3.566
[824] Volkmer A, Cross L, Highton L, Jackson C, Smith C, Brotherhood E, Harding EV, Mummery C, Rohrer J, Weil R, Yong KR, Crutch S, Hardy CJD (2024) 'Communication is difficult': Speech, language and communication needs of people with young onset or rarer forms of non-language led dementia. *International Journal of Language & Communication Disorders* **59**, 1553-1577. doi:10.1111/1460-6984.13018
[825] Waddington C, Harding E, Brotherhood EV, Abbott ID, Barker S, Camic PM, Ezeofor V, Gardner H, Grillo A, Hardy C, Hoare Z, McKee-Jackson R, Moore K, O'Hara T, Roberts J, Rossi-Harries S, Suarez-Gonzalez A, Sullivan MP, Edwards RT, Williams MV, Walton

J, Willoughby A, Windle G, Winrow E, Wood O, Zimmermann N, Crutch SJ, Stott J (2022) The Development of Videoconference-Based Support for People Living With Rare Dementias and Their Carers: Protocol for a 3-Phase Support Group Evaluation. *Jmir Research Protocols* **11**. doi:10.2196/35376
[826] Wajman JR, Cecchini MA, Bertolucci PHF, Mansur LL (2019) Quanti-qualitative components of the semantic verbal fluency test in cognitively healthy controls, mild cognitive impairment, and dementia subtypes. *Applied Neuropsychology-Adult* **26**, 533-542. doi:10.1080/23279095.2018.1465426
[827] Watanabe M, Araki W, Takao C, Maeda C, Tominaga R, Kimura Y, Nayanar G, Tu TTH, Asada T, Toyofuku A (2024) A case with burning mouth syndrome followed by dementia with Lewy bodies: a case report. *Frontiers in Psychiatry* **14**. doi:10.3389/fpsyt.2023.1329171
[828] Watermeyer TJ, Hindle JV, Roberts J, Lawrence CL, Martyr A, Lloyd-Williams H, Brand A, Gutting P, Hoare Z, Edwards RT, Clare L (2016) Goal Setting for Cognitive Rehabilitation in Mild to Moderate Parkinson's Disease Dementia and Dementia with Lewy Bodies. *Parkinsons Disease* **2016**. doi:10.1155/2016/8285041
[829] Williams DR, Warren JD, Lees AJ (2008) Using the presence of visual hallucinations to differentiate Parkinson's disease from atypical parkinsonism. *Journal of Neurology Neurosurgery and Psychiatry* **79**, 652-655. doi:10.1136/jnnp.2007.124677
[830] Yang BJ, Yang ZQ, Liu H, Qi H (2023) Dynamic modelling and tristability analysis of misfolded α-synuclein degraded via autophagy in Parkinson's disease. *Biosystems* **233**. doi:10.1016/j.biosystems.2023.105036
[831] Yumoto A, Suwa S (2021) Difficulties and associated coping methods regarding visual hallucinations caused by dementia with Lewy bodies. *Dementia-International Journal of Social Research and Practice* **20**, 291-307. doi:10.1177/1471301219879541
[832] Zhou S, Meng QC, Li LY, Hai L, Wang ZX, Li ZC, Sun YL (2021) Identification of a Qualitative Signature for the Diagnosis of Dementia With Lewy Bodies. *Frontiers in Genetics* **12**. doi:10.3389/fgene.2021.758103
[833] Abreu E, François J, Lambert W, Pérez J (2022) A Class of Positive Semi-discrete Lagrangian-Eulerian Schemes for Multidimensional Systems of Hyperbolic Conservation Laws. *Journal of Scientific Computing* **90**. doi:10.1007/s10915-021-01712-8
[834] Aurélie de Rus Jacquet RS, Suresh K. Ghimire, Jean-Christophe Rochet a (2014) Nepalese traditional medicine and symptoms related to Parkinson׳s disease and other disorders: Patterns of the usage of plant resources along the Himalayan altitudinal range. *Journal of Ethnopharmacology* **153**, 178-189. doi:https://doi.org/10.1016/j.jep.2014.02.016
[835] Chaudhary N, Aggarwal Y, Singh N, Sinha RK (2015) ELECTRONIC ANALOGY TO SIMULATE AND PREDICT THE DYNAMICS OF CELLULAR MECHANISM OF PARKINSON'S DISEASE STIMULATED BY ENVIRONMENTAL FACTORS. *Biomedical Engineering-Applications Basis Communications* **27**. doi:10.4015/s1016237215500519

[836] Chhabra A, Singh G, Ieee (2006) in *International Conference on Advanced Computing and Communications*, Mangalore, INDIA, pp. 56-59.
[837] Dantonio F, Teghil A, Boccia M, Gabrielli GB, Giulietti G, Conti D, Suppa A, Fabbrini A, Fiorelli M, Caramia F, Bruno G, Guariglia C, Aarsland D, Ffytche D (2024) Distinct grey and white matter changes are associated with the phenomenology of visual hallucinations in Lewy Body Disease. *Scientific Reports* **14**. doi:10.1038/s41598-024-65536-w
[838] Emirie TB, Gebremeskel MM (2024) The prevalence and effect of destructive leadership behavior on teachers' organizational commitment in the post-COVID-19 period: a case study of secondary schools in Amhara Regional State. *Cogent Education* **11**. doi:10.1080/2331186x.2024.2392426
[839] Golenkov AV, Filonenko VA, Sergeeva AI, Filonenko AV, Zolnikov ZI (2021) SUICIDAL BEHAVIOR IN DEMENTIA. *Suicidology* **12**, 91-113. doi:10.32878/suiciderus.21-12-02(43)-91-113
[840] Hirczy S, Salinas M (2022) Clinical Presentation, Diagnosis, and Pathogenesis of Dementia With Lewy Bodies. *Psychiatric Annals* **52**, 398-403. doi:10.3928/00485713-20220907-01
[841] Koo B (2022) A novel implicit method of characteristics using pressure-referenced correction for transient flow in natural gas pipelines. *Journal of Natural Gas Science and Engineering* **104**. doi:10.1016/j.jngse.2022.104665
[842] Obrien HL, Chen AT, Kaneshiro J, Zaslavsky O (2024) User Engagement in an Online Digital Health Intervention to Promote Problem Solving. *Interacting with Computers* **36**, 355-369. doi:10.1093/iwc/iwae030
[843] Ogura H (2009) Pharmacological Overview and Future Perspectives of Cholinergic Therapy in Alzheimer's Disease. *Current Drug Therapy* **4**, 65-72. doi:10.2174/157488509787081868
[844] Perinova P, Plchova L, Buskova J, Kemlink D, Losada V, Dostalova S, Vorlova T, Sonka K (2018) Patients with idiopathic REM sleep behavior disorder follow-up - phenoconversion into parkinsonian syndrome and dementia. *Ceska a Slovenska Neurologie a Neurochirurgie* **81**, 205-207. doi:10.14735/amcsnn2018205
[845] Sahgal A, Galloway PH, McKeith IG, Edwardson JA, Lloyd S (1992) A COMPARATIVE-STUDY OF ATTENTIONAL DEFICITS IN SENILE DEMENTIAS OF ALZHEIMER AND LEWY BODY TYPES. *Dementia* **3**, 350-354. doi:10.1159/000107037
[846] Tisserand A, Blanc F, Muller C, Durand H, Demuynck C, Ravier A, Sanna L, de Sousa PL, Botzung A, Mondino M, Philippi N (2024) Neuroimaging of autobiographical memory in dementia with Lewy bodies: a story of insula. *Brain Communications* **6**. doi:10.1093/braincomms/fcae272
[847] Wójcik D, Szczechowiak K (2019) Selected versions of the clock test in clinical practice - a comparative analysis of quantitative and qualitative scoring systems. *Aktualnosci Neurologiczne* **19**, 83-90. doi:10.15557/an.2019.0012
[848] Zhang XT, Ying LA (2005) Dependence of qualitative behavior of the numerical solutions on the ignition temperature for a combustion model. *Journal of Computational Mathematics* **23**, 337-350.

[849] Bentley A, Salifu Y, Walshe C (2021) Applying an Analytical Process to Longitudinal Narrative Interviews With Couples Living and Dying With Lewy Body Dementia. *International Journal of Qualitative Methods* **20**. doi:10.1177/16094069211060653

[850] Agarwal K, Backler W, Bayram E, Bloom L, Boeve BF, Cha JH, Denslow M, Ferman TJ, Galasko D, Galvin JE, Gomperts SN, Irizarry MC, Kantarci K, Kaushik H, Kietlinski M, Koenig A, Leverenz JB, McKeith I, McLean PJ, Montine TJ, Moose SO, O'Brien JT, Panier V, Ramanathan S, Ringel MS, Scholz SW, Small J, Sperling RA, Taylor A, Taylor JP, Ward RA, Witten L, Hyman BT (2024) Lewy body dementia: Overcoming barriers and identifying solutions. *Alzheimer's & Dementia: The Journal of the Alzheimer's Association* **20**, 2298-2308. doi:10.1002/alz.13674

[851] Armstrong MJ, Alliance S, Corsentino P, Lunde A, Taylor A (2022) Informal caregiver experiences at the end-of-life of individuals living with dementia with Lewy bodies: An interview study. *Dementia: The International Journal of Social Research and Practice* **21**, 287-303. doi:10.1177/14713012211038428

[852] Armstrong MJ, Gamez N, Alliance S, Majid T, Taylor AS, Kurasz AM, Patel B, Smith GE (2021) Clinical care and unmet needs of individuals with dementia with Lewy bodies and caregivers: An interview study. *Alzheimer Disease and Associated Disorders* **35**, 327-334. doi:10.1097/WAD.0000000000000459

[853] Ballard C, O'Brien J, Tovee M (2002) 'Qualitative performance characteristics differentiate dementia with Lewy bodies and Alzheimer's disease:' Comment. *Journal of Neurology, Neurosurgery & Psychiatry* **72**, 565-566. doi:10.1136/jnnp.72.5.565

[854] Bradshaw J, Saling M, Hopwood M, Anderson V, Brodtmann A (2004) Fluctuating cognition in dementia with Lewy bodies and Alzheimer's disease is qualitatively distinct. *Journal of Neurology, Neurosurgery & Psychiatry* **75**, 382-387. doi:10.1136/jnnp.2002.002576

[855] Brayne C, Richardson K, Matthews FE, Fleming J, Hunter S, Xuereb JH, Paykeld E, Mukaetova-Ladinska EB, Huppert FA, O'Sullivan A, Dening T (2009) Neuropathological correlates of dementia in over-80-year-old brain donors from the population-based Cambridge City over-75s Cohort (CC75C) study. *Journal of Alzheimer's Disease* **18**, 645-658. doi:10.3233/JAD-2009-1182

[856] Bukhatwa S, Zeng B-Y, Rose S, Jenner P (2010) A comparison of changes in proteasomal subunit expression in the substantia nigra in Parkinson's disease, multiple system atrophy and progressive supranuclear palsy. *Brain Research* **1326**, 174-183. doi:10.1016/j.brainres.2010.02.045

[857] Burkhard PR, Sanchez J-C, Landis T, Hochstrasser DF (2001) CSF detection of the 14-3-3 protein in unselected patients with dementia. *Neurology* **56**, 1528-1533. doi:10.1212/WNL.56.11.1528

[858] Caffarra P, Gardini S, Dieci F, Copelli S, Maset L, Concari L, Farina E, Grossi E (2013) The qualitative scoring MMSE pentagon test (QSPT): A new method for differentiating dementia with Lewy Body from Alzheimer’s disease. *Behavioural Neurology* **27**, 213-220. doi:10.1155/2013/728158

[859] Cagnin A, Di Lorenzo R, Marra C, Bonanni L, Cupidi C, Laganà V, Rubino E, Vacca A, Provero P, Isella V, Vanacore N, Agosta F, Appollonio I, Caffarra P, Pettenuzzo I,

Sambati R, Quaranta D, Guglielmi V, Logroscino G, Filippi M, Tedeschi G, Ferrarese C, Rainero I, Bruni AC (2020) Behavioral and psychological effects of coronavirus disease-19 quarantine in patients with dementia. *Frontiers in Psychiatry* **11**. doi:10.3389/fpsyt.2020.578015

[860] Carli G, Caminiti SP, Galbiati A, Marelli S, Casoni F, Padovani A, Ferini-Strambi L, Perani D (2020) In-vivo signatures of neurodegeneration in isolated rapid eye movement sleep behaviour disorder. *European Journal of Neurology* **27**, 1285-1295. doi:10.1111/ene.14215

[861] Cummings J, Emre M, Aarsland D, Tekin S, Dronamraju N, Lane R (2010) Effects of rivastigmine in Alzheimer's disease patients with and without hallucinations. *Journal of Alzheimer's Disease* **20**, 301-311. doi:10.3233/JAD-2010-1362

[862] Doubleday EK, Snowden JS, Varma AR, Neary D (2002) Qualitative performance characteristics differentiate dementia with Lewy bodies and Alzheimer's disease. *Journal of Neurology, Neurosurgery & Psychiatry* **72**, 602-607. doi:10.1136/jnnp.72.5.602

[863] Eversfield CL, Orton LD (2019) Auditory and visual hallucination prevalence in Parkinson's disease and dementia with Lewy bodies: A systematic review and meta-analysis. *Psychological Medicine* **49**, 2342-2353. doi:10.1017/S0033291718003161

[864] Falque A, Jordanis M, Landré L, Loureiro de Sousa P, Mondino M, Furcieri E, Blanc F (2022) Neural basis of impaired narrative discourse comprehension in prodromal and mild dementia with Lewy bodies. *Frontiers in Aging Neuroscience* **14**. doi:10.3389/fnagi.2022.939973

[865] Farfel JM, Nitrini R, Suemoto CK, Grinberg LT, Ferretti REL, Paraizo Leite RE, Tampellini E, Lima L, Farias DS, Neves RC, Rodriguez RD, Menezes PR, Fregni F, Bennett DA, Pasqualucci CA, Filho WJ (2013) Very low levels of education and cognitive reserve: A clinicopathologic study. *Neurology* **81**, 650-657. doi:10.1212/WNL.0b013e3182a08f1b

[866] Fernández-Arcos A, Morenas-Rodríguez E, Santamaria J, Sánchez-Valle R, Lladó A, Gaig C, Lleó A, Iranzo A (2019) Clinical and video-polysomnographic analysis of rapid eye movement sleep behavior disorder and other sleep disturbances in dementia with Lewy bodies. *Sleep: Journal of Sleep and Sleep Disorders Research* **42**, 1-18. doi:10.1093/sleep/zsz086

[867] Foy CML, Nicholas H, Hollingworth P, Boothby H, Willams J, Brown RG, Al-Sarraj S, Lovestone S (2007) Diagnosing Alzheimer's disease--non-clinicians and computerised algorithms together are as accurate as the best clinical practice. *International Journal of Geriatric Psychiatry* **22**, 1154-1163. doi:10.1002/gps.1810

[868] Gibson LL, Grinberg LT, ffytche D, Leite REP, Rodriguez RD, Ferretti-Rebustini REL, Pasqualucci CA, Nitrini R, Jacob-Filho W, Aarsland D, Suemoto CK (2022) Neuropathological correlates of neuropsychiatric symptoms in dementia. *Alzheimer's & Dementia: The Journal of the Alzheimer's Association*. doi:10.1002/alz.12765

[869] Gossard TR, Teigen LN, Yoo S, Timm PC, Jagielski J, Bibi N, Feemster JC, Steele T, Carvalho DZ, Junna MR, Lipford MC, Peikert MT, LeClair-Visonneau L, McCarter SJ, Boeve BF, Silber MH, Hirsch J, Sharp RR, St. Louis EK (2023) Patient values and

preferences regarding prognostic counseling in isolated REM sleep behavior disorder. *Sleep: Journal of Sleep and Sleep Disorders Research* **46**, 1-11. doi:10.1093/sleep/zsac244
[870] Guo X, Meng Q, Zuo M, Su Z, Gan J, Li XD, Zhu H, Gang B, Niu J, Liu S, Ji Y (2023) Costs of dementia with Lewy bodies: A Chinese multicenter cross-sectional study. *International Journal of Geriatric Psychiatry* **38**, 1-9. doi:10.1002/gps.5848
[871] Harciarek M, Kertesz A (2008) The prevalence of misidentification syndromes in neurodegenerative diseases. *Alzheimer Disease and Associated Disorders* **22**, 163-169. doi:10.1097/WAD.0b013e3181641341
[872] Huang W-C, Chang M-C, Wang W-F, Jhang K-M (2022) A comparison of caregiver burden for different types of dementia: An 18-month retrospective cohort study. *Frontiers in Psychology* **12**. doi:10.3389/fpsyg.2021.798315
[873] Huang W-C, Chang M-C, Wang W-F, Jhang K-M (2023) Corrigendum: A comparison of caregiver burden for different types of dementia: An 18-month retrospective cohort study. *Frontiers in Psychology* **14**. doi:10.3389/fpsyg.2023.1224716
[874] Iaccarino L, Marelli S, Iannaccone S, Magnani G, Ferini-Strambi L, Perani D (2016) Severe Brain Metabolic Decreases Associated with REM Sleep Behavior Disorder in Dementia with Lewy Bodies. *Journal of Alzheimer's Disease* **52**, 989-997. doi:10.3233/JAD-151000
[875] Ikeda M, Mori E, Kosaka K, Iseki E, Hashimoto M, Matsukawa N, Matsuo K, Nakagawa M (2013) Long-term safety and efficacy of donepezil in patients with dementia with Lewy bodies: Results from a 52-week, open-label, multicenter extension study. *Dementia and Geriatric Cognitive Disorders* **36**, 229-241. doi:10.1159/000351672
[876] Ikeda M, Mori E, Orimo S, Yamada T, Konishi O (2023) Efficacy of adjunctive therapy with zonisamide versus increased dose of levodopa for motor symptoms in patients with dementia with Lewy bodies: The randomized, controlled, non-inferiority DUEL study. *Journal of Alzheimer's Disease* **95**, 251-264. doi:10.3233/JAD-230335
[877] Killen A, Flynn D, O'Brien N, Taylor J-P (2022) The feasibility and acceptability of a psychosocial intervention to support people with dementia with Lewy bodies and family care partners. *Dementia: The International Journal of Social Research and Practice* **21**, 77-93. doi:10.1177/14713012211028501
[878] Kirk A (2007) Target symptoms and outcome measures: Cognition. *The Canadian Journal of Neurological Sciences / Le Journal Canadien Des Sciences Neurologiques* **34**, S42-S46. doi:10.1017/S0317167100005552
[879] Lee H, Brekelmans GJF, Roks G (2015) The EEG as a diagnostic tool in distinguishing between dementia with Lewy bodies and Alzheimer's disease. *Clinical Neurophysiology* **126**, 1735-1739. doi:10.1016/j.clinph.2014.11.021
[880] Leggett AN, Zarit S, Taylor A, Galvin JE (2011) Stress and burden among caregivers of patients with Lewy body dementia. *The Gerontologist* **51**, 76-85. doi:10.1093/geront/gnq055
[881] Leu-Semenescu S, Arnulf I (2010) Agitation nocturne chez la personne âgée: Et si c'était une parasomnie? = Disruptive nocturnal behavior in elderly subjects: Could it be a parasomnia? *Annales de Gérontologie* **8**, 97-109.

[882] Lim S-Y, Dy Closas AMF, Tan AH, Lim JL, Tan YJ, Vijayanathan Y, Tay YW, Abdul Khalid Rb, Ng WK, Kanesalingam R, Martinez-Martin P, Ahmad Annuar A, Lit LC, Foo JN, Lim WK, Ng ASL, Tan E-K (2023) New insights from a multi-ethnic Asian progressive supranuclear palsy cohort. *Parkinsonism & Related Disorders* **108**. doi:10.1016/j.parkreldis.2023.105296

[883] Lin W, Xie Y-C, Cheng P-Y, Dong L-Y, Hung G-U, Chiu P-Y (2018) Association of visual hallucinations with very mild degenerative dementia due to dementia with Lewy bodies. *PLoS ONE* **13**. doi:10.1371/journal.pone.0205909

[884] Lindeberg S, Müller N, Samuelsson C (2022) Conversations in dementia with lewy bodies: Resources and barriers in communication. *International Journal of Language & Communication Disorders*. doi:10.1111/1460-6984.12799

[885] Liu S, Liu J, Wang X-D, Shi Z, Zhou Y, Li J, Yu T, Ji Y (2018) Caregiver burden, sleep quality, depression, and anxiety in dementia caregivers: A comparison of frontotemporal lobar degeneration, dementia with Lewy bodies, and Alzheimer's disease. *International Psychogeriatrics* **30**, 1131-1138. doi:10.1017/S1041610217002630

[886] Maclin JMA, Wang T, Xiao S (2019) Biomarkers for the diagnosis of Alzheimer's disease, dementia Lewy body, frontotemporal dementia and vascular dementia. *General Psychiatry* **32**, 5-13. doi:10.1136/gpsych-2019-100054

[887] McIntyre A, Harding E, Yong KXX, Sullivan MP, Gilhooly M, Gilhooly K, Woodbridge R, Crutch S (2019) Health and social care practitioners' understanding of the problems of people with dementia-related visual processing impairment. *Health & Social Care in the Community* **27**, 982-990. doi:10.1111/hsc.12715

[888] Mitolo M, Salmon DP, Gardini S, Galasko D, Grossi E, Caffarra P (2014) The new Qualitative Scoring MMSE Pentagon Test (QSPT) as a valid screening tool between autopsy-confirmed dementia with Lewy bodies and Alzheimer's disease. *Journal of Alzheimer's Disease* **39**, 823-832. doi:10.3233/JAD-131403

[889] Mori E, Ikeda M, Iseki E, Katayama S, Nagahama Y, Ohdake M, Takase T (2024) Efficacy and safety of donepezil in patients with dementia with Lewy bodies: results from a 12-week multicentre, randomised, double-blind, and placebo-controlled phase IV study. *Psychogeriatrics* **24**, 542-554. doi:10.1111/psyg.13091

[890] Mori E, Ikeda M, Kosaka K (2012) Donepezil for dementia with Lewy bodies: A randomized, placebo-controlled trial. *Annals of Neurology* **72**, 41-52. doi:10.1002/ana.23557

[891] Mori E, Ikeda M, Ohdake M (2024) Donepezil for dementia with Lewy bodies: Meta-analysis of multicentre, randomised, double-blind, placebo-controlled phase II, III, and, IV studies. *Psychogeriatrics* **24**, 589-596. doi:10.1111/psyg.13101

[892] Mosimann UP, Rowan EN, Partington CE, Collerton D, Littlewood E, O'Brien JT, Burn DJ, McKeith IG (2006) Characteristics of visual hallucinations in Parkinson disease dementia and dementia with Lewy bodies. *The American Journal of Geriatric Psychiatry* **14**, 153-160. doi:10.1097/01.JGP.0000192480.89813.80

[893] Murayama N, Masubuchi Y, Kimura A, Uchiyama K, Yamagata M, Ota K, Iseki E (2023) A simple method to evaluate the pentagon copy test of the mini-mental state

examination for the differentiation of dementia with lewy bodies. *Applied Neuropsychology: Adult*. doi:10.1080/23279095.2023.2200948
[894] Oliveira FPM, Walker Z, Walker RWH, Attems J, Castanheira JC, Silva Â, Oliveira C, Vaz S, Silva M, Costa DC (2021) ^{123}I-FP-CIT SPECT in dementia with Lewy bodies, Parkinson's disease and Alzheimer's disease: a new quantitative analysis of autopsy confirmed cases. *Journal of Neurology, Neurosurgery & Psychiatry* **92**, 662-667. doi:10.1136/jnnp-2020-324606
[895] Perri R, Fadda L, Caltagirone C, Carlesimo GA (2013) Word list and story recall elicit different patterns of memory deficit in patients with Alzheimer's disease, frontotemporal dementia, subcortical Ischemic Vascular Disease, and Lewy body dementia. *Journal of Alzheimer's Disease* **37**, 99-107. doi:10.3233/JAD-130347
[896] Piersma D, Fuermaier ABM, De Waard D, Davidse RJ, De Groot J, Doumen MJA, Bredewoud RA, Claesen R, Lemstra AW, Scheltens P, Vermeeren A, Ponds R, Verhey F, De Deyn PP, Brouwer WH, Tucha O (2018) Assessing fitness to drive in patients with different types of dementia. *Alzheimer Disease and Associated Disorders* **32**, 70-75. doi:10.1097/WAD.0000000000000221
[897] Piscopo P, Marcon G, Piras MR, Crestini A, Campeggi LM, Deiana E, Cherchi R, Tanda F, Deplano A, Vanacore N, Tagliavini F, Pocchiari M, Giaccone G, Confaloni A (2008) A novel PSEN2 mutation associated with a peculiar phenotype. *Neurology* **70**, 1549-1554. doi:10.1212/01.wnl.0000310643.53587.87
[898] Rahkonen T, Eloniemi-Sulkava U, Rissanen S, Vatanen A, Viramo P, Sulkava R (2003) Dementia with Lewy bodies according to the consensus criteria in a general population aged 75 years or older. *Journal of Neurology, Neurosurgery & Psychiatry* **74**, 720-724. doi:10.1136/jnnp.74.6.720
[899] Sahgal A, Galloway PH, McKeith IG, Edwardson JA (1992) A comparative study of attentional deficits in senile dementias of Alzheimer and Lewy body types. *Dementia* **3**, 350-354.
[900] Sakurai K, Kaneda D, Uchida Y, Inui S, Bundo M, Akagi A, Nihashi T, Kimura Y, Kato T, Ito K, Ohashi W, Hashizume Y (2021) Can medial temporal impairment be an imaging red flag for neurodegeneration in disproportionately enlarged subarachnoid space hydrocephalus? *Journal of Alzheimer's Disease* **83**, 1199-1209. doi:10.3233/JAD-210535
[901] Snowden JS, Thompson JC, Stopford CL, Richardson AMT, Gerhard A, Neary D, Mann DMA (2011) The clinical diagnosis of early-onset dementias: Diagnostic accuracy and clinicopathological relationships. *Brain: A Journal of Neurology* **134**, 2478-2492. doi:10.1093/brain/awr189
[902] Svendsboe EJ (2022) in *Dissertation Abstracts International: Section B: The Sciences and Engineering* ProQuest Information & Learning.
[903] Tan LPL, Herrmann N, Mainland BJ, Shulman K (2015) Can clock drawing differentiate Alzheimer's disease from other dementias? *International Psychogeriatrics* **27**, 1649-1660. doi:10.1017/S1041610215000939
[904] Tröster AI, Browner N (2013) Movement disorders with dementia in older adults In *Handbook on the neuropsychology of aging and dementia.*, Ravdin LD, Katzen HL, eds. Springer Science + Business Media, New York, NY, pp. 333-361.

[905] Tsopelas C, Stewart R, Savva GM, Brayne C, Ince P, Thomas A, Matthews FE (2011) Neuropathological correlates of late-life depression in older people. *The British Journal of Psychiatry* **198**, 109-114. doi:10.1192/bjp.bp.110.078816

[906] Vatter S, McDonald KR, Stanmore E, McCormick SA, Clare L, Leroi I (2020) A brief psychometric and clinimetric evaluation of self-report burden and mental health measures completed by care partners of people with Parkinson's-related dementia. *International Psychogeriatrics* **32**, 875-880. doi:10.1017/S1041610220000605

[907] Vieregge P, Hagenah J, Heberlein I, Klein C, Ludin HP (1999) Parkinson's disease in twins: A follow up study. *Neurology* **53**, 566-572. doi:10.1212/WNL.53.3.566

[908] Volkmer A, Cross L, Highton L, Jackson C, Smith C, Brotherhood E, Harding EV, Mummery C, Rohrer J, Weil R, Yong K, Crutch S, Hardy CJD (2024) 'communication is difficult': Speech, language and communication needs of people with young onset or rarer forms of non-language led dementia. *International Journal of Language & Communication Disorders*. doi:10.1111/1460-6984.13018

[909] Wajman JR, Cecchini MA, Bertolucci PHF, Mansur LL (2019) Quanti-qualitative components of the semantic verbal fluency test in cognitively healthy controls, mild cognitive impairment, and dementia subtypes. *Applied Neuropsychology: Adult* **26**, 533-542. doi:10.1080/23279095.2018.1465426

[910] Williams DR, Warren JD, Lees AJ (2008) Using the presence of visual hallucinations to differentiate Parkinson's disease from atypical Parkinsonism. *Journal of Neurology, Neurosurgery & Psychiatry* **79**, 652-655. doi:10.1136/jnnp.2007.124677

[911] Yumoto A, Suwa S (2021) Difficulties and associated coping methods regarding visual hallucinations caused by dementia with Lewy bodies. *Dementia: The International Journal of Social Research and Practice* **20**, 291-307. doi:10.1177/1471301219879541

[912] Bartusch DJ, Matsueda RL (1996) Gender, reflected appraisals, and labeling: A cross-group test of an internationist theory of delinquency. *Social Forces* **75**, 145-176. doi:10.2307/2580760

[913] Bussè C, Anselmi P, Pompanin S, Zorzi G, Fragiacomo F, Camporese G, Di Bernardo GA, Semenza C, Caffarra P, Cagnin A (2017) Specific verbal memory measures may distinguish Alzheimer's disease from dementia with Lewy bodies. *Journal of Alzheimer's Disease* **59**, 1009-1015. doi:10.3233/JAD-170154

[914] Espínola Nadurille M, Dolores Velasco F, Ramírez-Bermúdez J, Sosa Ortiz AL, Becerra Pino M (2007) Baja frecuencia clínica de la demencia por cuerpos de Lewy en el Instituto de Neurología de México = Low clinical frequency of Lewy body dementia in the Institute of Neurology in Mexico. *Revista Española de Geriatría y Gerontología* **42**, 328-332. doi:10.1016/S0211-139X(07)73571-0

[915] Fernández-Santiago R, Iranzo A, Gaig C, Serradell M, Fernández M, Tolosa E, Santamaría J, Ezquerra M (2016) Absence of LRRK2 mutations in a cohort of patients with idiopathic REM sleep behavior disorder. *Neurology* **86**, 1072-1073. doi:10.1212/WNL.0000000000002304

[916] Harciarek M, Sitek EJ, Barczak A (2017) Dementias and the frontal lobes In *Executive functions in health and disease.*, Goldberg E, ed. Elsevier Academic Press, San Diego, CA, pp. 445-486.

[917] Hashimoto K (2013) Potential role of the sigma-1 receptor chaperone in the beneficial effects of donepezil in dementia with Lewy bodies. *Clinical Psychopharmacology and Neuroscience* **11**, 43-44. doi:10.9758/cpn.2013.11.1.43

[918] Iwasaki K, Maruyama M, Tomita N, Furukawa K, Nemoto M, Fujiwara H, Seki T, Fujii M, Kodama M, Arai H (2005) Effects of the Traditional Chinese Herbal Medicine Yi-Gan San for Cholinesterase Inhibitor-Resistant Visual Hallucinations and Neuropsychiatric Symptoms in Patients With Dementia With Lewy Bodies. *The Journal of Clinical Psychiatry* **66**, 1612-1613. doi:10.4088/JCP.v66n1219a

[919] Jellinger KA (2006) Pathological substrate of dementia in Parkinson's disease--Its relation to DLB and DLBD. *Parkinsonism & Related Disorders* **12**, 119-120. doi:10.1016/j.parkreldis.2005.09.001

[920] Landis BN, Burkhard PR (2009) 'Olfactory disturbance in Parkinson disease': In reply. *Archives of Neurology* **66**, 805-806. doi:10.1001/archneurol.2009.88

[921] Lindeberg S (2024) in *Dissertation Abstracts International: Section B: The Sciences and Engineering* ProQuest Information & Learning.

[922] Lyus R, Kissima J, Yoseph M, Damas A, Gray W, Walker R, Paddick S-M, Robinson L, Dotchin C (2022) Proportions of dementia subtypes in rural Tanzania. *The American Journal of Geriatric Psychiatry* **30**, 424-425. doi:10.1016/j.jagp.2021.09.007

[923] Nomura T, Nakashima K, Inoue Y, Högl B (2011) Authors' reply to the comments of Miyamoto et al regarding 'Cardiac ^{123}I-MIBG accumulation in Parkinson's disease differs in association with REM sleep behavior disorder'. *Parkinsonism & Related Disorders* **17**, 654-654. doi:10.1016/j.parkreldis.2010.12.015

[924] Rockwood K, Gauthier S (2006) *Trial designs and outcomes in dementia therapeutic research*, Taylor & Francis, Philadelphia, PA.

[925] Snowden JS (1994) Contribution to the differential diagnosis of dementias: I Neuropsychology. *Reviews in Clinical Gerontology* **4**, 227-234. doi:10.1017/S0959259800003853

[926] Tröster AI, Abbott A (2019) Movement disorders with dementia in older adults In *Handbook on the neuropsychology of aging and dementia., 2nd ed.*, Ravdin LD, Katzen HL, eds. Springer Nature Switzerland AG, Cham, pp. 543-575.

[927] Weintraub D (2010) Psychiatric and cognitive complications of Parkinson's disease and dementia with Lewy bodies. *Primary Psychiatry* **17**, 25-28.

[928] Westervelt HJ, Bruce JM, Faust MA (2016) Distinguishing Alzheimer's disease and dementia with Lewy bodies using cognitive and olfactory measures. *Neuropsychology* **30**, 304-311. doi:10.1037/neu0000230

[929] Whitworth A, Lesser R, McKeith I (1999) Profiling conversation in Parkinson's disease with cognitive impairment. *Aphasiology* **13**, 407-425. doi:10.1080/026870399402154

[930] Bentley A (2024) in *Dissertation Abstracts International Section A: Humanities and Social Sciences* ProQuest Information & Learning.

[931] Armstrong MJ, Gamez N, Alliance S, Majid T, Taylor AS, Kurasz AM, Patel B, Smith GE (2021) Clinical Care and Unmet Needs of Individuals With Dementia With Lewy Bodies and Caregivers: An Interview Study. *Alzheimer Disease & Associated Disorders* **35**, 327-334. doi:10.1097/WAD.0000000000000459

[932] Ballard C, McKeith I, Harrison R, O'Brien J, Thompson P, Lowery K, Perry R, Ince P, Ballard C, McKeith I, Harrison R, O'Brien J, Thompson P, Lowery K, Perry R, Ince P (1997) A detailed phenomenological comparison of complex visual hallucinations in dementia with Lewy bodies and Alzheimer's disease. *International Psychogeriatrics*, 381-388.

[933] Ballard C, Patel A, Oyebode F, Wilcock G (1996) Cognitive decline in patients with Alzheimer's disease, vascular dementia and senile dementia of Lewy body type. *Age & Ageing* **25**, 209-213. doi:10.1093/ageing/25.3.209

[934] Bradshaw J, Saling M, Hopwood M, Anderson V, Brodtmann A, Bradshaw J, Saling M, Hopwood M, Anderson V, Brodtmann A (2004) Fluctuating cognition in dementia with Lewy bodies and Alzheimer's disease is qualitatively distinct. *Journal of Neurology, Neurosurgery & Psychiatry* **75**, 382-387. doi:10.1136/jnnp.2002.002576

[935] Brayne C, Richardson K, Matthews FE, Fleming J, Hunter S, Xuereb JH, Paykel E, Mukaetova-Ladinska EB, Huppert FA, O'Sullivan A, Dening T (2009) Neuropathological correlates of dementia in over-80-year-old brain donors from the population-based Cambridge city over-75s cohort (CC75C) study. *Journal of Alzheimer's Disease* **18**, 645-658. doi:10.3233/JAD-2009-1182

[936] Brown LJE, Aldridge Z, Pepper A, Leroi I, Dening KH (2022) 'It's just incredible the difference it has made': family carers' experiences of a specialist Lewy body dementia Admiral Nurse service. *Age & Ageing* **51**, 1-5. doi:10.1093/ageing/afac207

[937] de Rus Jacquet A, Timmers M, Ma SY, Thieme A, McCabe GP, Vest JHC, Lila MA, Rochet J-C (2017) Lumbee traditional medicine: Neuroprotective activities of medicinal plants used to treat Parkinson's disease-related symptoms. *Journal of Ethnopharmacology* **206**, 408-425. doi:10.1016/j.jep.2017.02.021

[938] Desmarais P, Massoud F, Filion J, Nguyen QD, Bajsarowicz P (2016) Quetiapine for Psychosis in Parkinson Disease and Neurodegenerative Parkinsonian Disorders: A Systematic Review. *Journal of Geriatric Psychiatry & Neurology* **29**, 227-236. doi:10.1177/0891988716640378

[939] Donaghy PC, Barnett N, Olsen K, Taylor JP, McKeith IG, O'Brien JT, Thomas AJ, Taylor J-P (2017) Symptoms associated with Lewy body disease in mild cognitive impairment. *International Journal of Geriatric Psychiatry* **32**, 1163-1171. doi:10.1002/gps.4742

[940] Doubleday EK, Snowden JS, Varma AR, Neary D (2002) Qualitative performance characteristics differentiate dementia with Lewy bodies and Alzheimer's disease. *Journal of Neurology, Neurosurgery & Psychiatry*, 602-607. doi:10.1136/jnnp.72.5.602

[941] Duro D, Tábuas-Pereira M, Freitas S, Santiago B, Botelho MA, Santana I (2018) Validity and Clinical Utility of Different Clock Drawing Test Scoring Systems in Multiple Forms of Dementia. *Journal of Geriatric Psychiatry & Neurology* **31**, 114-122. doi:10.1177/0891988718774432

[942] Falque A, Jordanis M, Landré L, Loureiro de Sousa P, Mondino M, Furcieri E, Blanc F (2022) Neural basis of impaired narrative discourse comprehension in prodromal and mild dementia with lewy bodies. *Frontiers in Aging Neuroscience* **14**, 1-9. doi:10.3389/fnagi.2022.939973

[943] Farfel JM, Nitrini R, Suemoto CK, Grinberg LT, Ferretti REL, Leite REP, Tampellini E, Lima L, Farias DS, Neves RC, Rodriguez RD, Menezes PR, Fregni F, Bennett DA, Pasqualucci CA, Jacob Filho W (2013) Very low levels of education and cognitive reserve: a clinicopathologic study. *Neurology* **81**, 650-657. doi:10.1212/WNL.0b013e3182a08f1b
[944] Foy CML, Nicholas H, Hollingworth P, Boothby H, Willams J, Brown RG, Al-Sarraj S, Lovestone S, Foy CML, Nicholas H, Hollingworth P, Boothby H, Willams J, Brown RG, Al-Sarraj S, Lovestone S (2007) Diagnosing Alzheimer's disease--non-clinicians and computerised algorithms together are as accurate as the best clinical practice. *International Journal of Geriatric Psychiatry* **22**, 1154-1163. doi:10.1002/gps.1810
[945] Galvin JE, Malcom H, Johnson D, Morris JC, Galvin JE, Malcom H, Johnson D, Morris JC (2007) Personality traits distinguishing dementia with Lewy bodies from Alzheimer disease. *Neurology* **68**, 1895-1901. doi:10.1212/01.wnl.0000263131.80945.ad
[946] Gubert E, do Prado ML (2011) Desafios na prática pedagógica na educação profissional em enfermagem. *Revista Eletrônica de Enfermagem* **13**, 285-295. doi:10.5216/ree.v13i2.9036
[947] Hindle JV, Watermeyer TJ, Roberts J, Martyr A, Lloyd-Williams H, Brand A, Gutting P, Hoare Z, Edwards RT, Clare L (2016) Cognitive rehabilitation for Parkinson's disease dementia: a study protocol for a pilot randomised controlled trial. *Trials* **17**, 1-9. doi:10.1186/s13063-016-1253-0
[948] Ikeda M, Mori E, Kosaka K, Iseki E, Hashimoto M, Matsukawa N, Matsuo K, Nakagawa M (2013) Long-Term Safety and Efficacy of Donepezil in Patients with Dementia with Lewy Bodies: Results from a 52-Week, Open-Label, Multicenter Extension Study. *Dementia & Geriatric Cognitive Disorders* **36**, 229-241. doi:10.1159/000351672
[949] Jhoo JH, Kim KW, Huh Y, Lee S, Park JH, Lee JJ, Choi EA, han C, Choo IH, Youn JC, Lee D, Woo JI (2008) Prevalence of dementia and its subtypes in an elderly urban Korean population: results from the Korean Longitudinal Study on Health and Aging (KLoSHA). *Dementia & Geriatric Cognitive Disorders* **26**, 270-276. doi:10.1159/000160960
[950] Jreige M, Kurian GK, Perriraz J, Potheegadoo J, Bernasconi F, Stampacchia S, Blanke O, Alessandra G, Lejay N, Chiabotti PS, Rouaud O, Nicod Lalonde M, Schaefer N, Treglia G, Allali G, Prior JO (2023) The diagnostic performance of functional dopaminergic scintigraphic imaging in the diagnosis of dementia with Lewy bodies: an updated systematic review. *European Journal of Nuclear Medicine & Molecular Imaging* **50**, 1988-2035. doi:10.1007/s00259-023-06154-y
[951] Liu S, Wang X-D, Shi Z, Zhou Y, Li J, Ji Y, Liu J, Yu T (2018) Caregiver burden, sleep quality, depression, and anxiety in dementia caregivers: a comparison of frontotemporal lobar degeneration, dementia with Lewy bodies, and Alzheimer's disease. *International Psychogeriatrics* **44**, 1131-1138. doi:10.1017/S1041610217002630
[952] Mitolo M, Salmon DP, Gardini S, Galasko D, Grossi E, Caffarra P (2014) The new Qualitative Scoring MMSE Pentagon Test (QSPT) as a valid screening tool between

autopsy-confirmed dementia with Lewy bodies and Alzheimer's disease. *Journal of Alzheimer's Disease* **38**, 823-832. doi:10.3233/JAD-131403
[953] Mori E, Ikeda M, Kosaka K, Mori E, Ikeda M, Kosaka K (2012) Donepezil for dementia with Lewy bodies: a randomized, placebo-controlled trial. *Annals of Neurology* **72**, 41-52. doi:10.1002/ana.23557
[954] Park J, Howard H, Tolea MI, Galvin JE (2020) Perceived Benefits of Using Nonpharmacological Interventions in Older Adults With Alzheimer's Disease or Dementia With Lewy Bodies. *Journal of Gerontological Nursing* **46**, 37-46. doi:10.3928/00989134-20191217-01
[955] Piersma D, Fuermaier ABM, De Waard D, Davidse RJ, De Groot J, Doumen MJA, Bredewoud RA, Claesen R, Lemstra AW, Scheltens P, Vermeeren A, Ponds R, Verhey F, De Deyn PP, Brouwer WH, Tuch O, Tucha O (2018) Assessing Fitness to Drive in Patients With Different Types of Dementia. *Alzheimer Disease & Associated Disorders* **32**, 70-75. doi:10.1097/WAD.0000000000000221
[956] Rahkonen T, Eloniemi-Sulkava U, Rissanen S, Vatanen A, Viramo P, Sulkava R, Rahkonen T, Eloniemi-Sulkava U, Rissanen S, Vatanen A, Viramo P, Sulkava R (2003) Dementia with Lewy bodies according to the consensus criteria in a general population aged 75 years or older. *Journal of Neurology, Neurosurgery & Psychiatry* **74**, 720-724. doi:10.1136/jnnp.74.6.720
[957] Roque M, Salva A, Vellas B (2013) Malnutrition in community-dwelling adults with dementia (Nutrialz Trial). *Journal of Nutrition, Health & Aging* **17**, 295-299. doi:10.1007/s12603-012-0401-9
[958] Shindo A, Ueda Y, Kuzuhara S, Kokubo Y (2014) Neuropsychological study of amyotrophic lateral sclerosis and parkinsonism-dementia complex in Kii peninsula, Japan. *BMC Neurology* **14**, 151-151. doi:10.1186/1471-2377-14-151
[959] Tateno M, Kobayashi S, Shirasaka T, Furukawa Y, Fujii K, Morii H, Yasumura S, Utsumi K, Saito T (2008) Comparison of the usefulness of brain perfusion SPECT and MIBG myocardial scintigraphy for the diagnosis of dementia with Lewy bodies. *Dementia & Geriatric Cognitive Disorders* **26**, 453-457. doi:10.1159/000165918
[960] Vatter S, McDonald KR, Stanmore E, Clare L, McCormick SA, Leroi I (2018) A qualitative study of female caregiving spouses' experiences of intimate relationships as cognition declines in Parkinson's disease. *Age & Ageing* **47**, 604-610. doi:10.1093/ageing/afy049
[961] Volkmer A, Cross L, Highton L, Jackson C, Smith C, Brotherhood E, Harding EV, Mummery C, Rohrer J, Weil R, Yong K, Crutch S, Hardy CJD (2024) 'Communication is difficult': Speech, language and communication needs of people with young onset or rarer forms of non-language led dementia. *International Journal of Language & Communication Disorders* **59**, 1553-1577. doi:10.1111/1460-6984.13018
[962] Watermeyer TJ, Hindle JV, Roberts J, Lawrence CL, Martyr A, Lloyd-Williams H, Brand A, Gutting P, Hoare Z, Edwards RT, Clare L (2016) Goal Setting for Cognitive Rehabilitation in Mild to Moderate Parkinson’s Disease Dementia and Dementia with Lewy Bodies. *Parkinson's Disease (20420080)*, 1-8. doi:10.1155/2016/8285041

[963] Williams DR, Warren JD, Lees AJ, Williams DR, Warren JD, Lees AJ (2008) Using the presence of visual hallucinations to differentiate Parkinson's disease from atypical parkinsonism. *Journal of Neurology, Neurosurgery & Psychiatry* **79**, 652-655.
[964] Beek M, Steenoven I, Zande JJ, Barkhof F, Teunissen CE, Flier WM, Lemstra AW, van de Beek M, van Steenoven I, van der Zande JJ, van der Flier WM (2020) Prodromal Dementia With Lewy Bodies: Clinical Characterization and Predictors of Progression. *Movement Disorders* **35**, 859-867. doi:10.1002/mds.27997
[965] Killen A, Flynn D, De Brun A, O'Brien N, O'Brien J, Thomas AJ, McKeith I, Taylor JP (2016) Support and information needs following a diagnosis of dementia with Lewy bodies. *Int Psychogeriatr* **28**, 495-501. doi:10.1017/S1041610215001362
