# Supplementary material for: Examining the lived experience of dementia with Lewy bodies through qualitative research: A systematic review
Source: Alzheimers Dement. 2025 May 15;21(5):e70217. doi: 10.1002/alz.70217 (PMC12079417; doi:10.1002/alz.70217)
Supplement: Supplementary file 3 — Supporting Information [file ALZ-21-e70217-s001.pdf]

Caregiver Experiences

THEMATIC FINDINGS — FAMILY CAREGIVERS

SYMPTOMS & IMPACT OF SYMPTOMS

- Matterson, et al. [40] Themes: (1) symptoms of cognitive fluctuations were inconsistent; (2) both Pt and CG had sleep disturbances due to DLB. Poor sleep quality was a trigger for worsening cognitive fluctuations. Excessive daytime sleepiness was common. → Sleep disturbances; Cognitive fluctuations
- Yumoto and Suwa [28] Changes in the daily life of people with DLB: Most common symptoms were cognitive decline, visual hallucinations, RBSD, and parkinsonism. Difficulties were due to symptoms Pt was aware and unaware of. Themes: bodily discomfort (trouble moving/doing), daily obstacles (forgetting, communicating), psychological pain, isolation, frustration, depression. → Common symptoms; Functional impairment
- Volkmer, et al. [77] Themes for LBD group related to aphasia: (1) communication difficulties a key problem, (2) feeling of isolation due to speech issues (worse for CG than for Pt), (3) doing anything that helps, (4) low access to speech and language therapy. → Communication; Social impairment; Coping
- Gallop, et al. [78] nOH impacted 8 areas for CG: physical health, wellbeing, daily activities, work, social and leisure, relationships, finances, and independence → Impairments d/t hypotension
- Kew, et al. [48] Focused on assessing coverage of ICF. 402 concepts identified, 79% of which linked to ICF categories. Concerns were: (1) body structures, (2) body functions, (3) activities, (4) environmental factors, and (5) personal factors. Categories 1,2,3 were most common. LBD CG reported more challenges than AD CG. Specific challenges not listed. → Challenges faced by CG
- Vatter, et al. [50] Areas impacted were relationship satisfaction, intimacy, communication, care responsibilities, emotional distancing, frustration, resentment, anger, sadness, fear of future. Three themes related to impacts experienced by CG: (1) altered relationship; (2) care partner challenges; and (3) acceptance and adjustment. → Relationship impacts
- Bradshaw, et al. [45] DLB CG cited lapse in awareness or attention, lost ability to engage in meaningful cognitive or physical activity with short lived alterations in cognitive and functional abilities vs. AD CG reporting confusion and more persistent alterations → Cognitive impairments + functional impairments
- Armstrong, et al. [54] Themes: (1) caregivers drive care, (2) DLB symptoms affect EOL for CG, (3) CG role, (4) death + post-death, and (5) supports for CG. DLB features that impacted CG included: fluctuations, hallucinations, delusions, REM, Autonomic symptoms, Physical limitations, falls. → EOL experiences

CHALLENGES WITH CLINICAL CARE & DIAGNOSIS

- Galvin, et al. [56] Presenting symptom were cognitive (48%), motor (39%), or mixed 13%. Multiple visits (>6) and multiple doctors (3.3 ± 1.5) needed to establish LBD diagnosis; 31% took > 2 years. Initial diagnosis not LBD in 78%: PD, AD, FTD, MDD. → Presenting symptoms; Difficulties w/ diagnosis
- Armstrong, et al. [57] Barriers: systems and physician issues, DLB issues, diagnostic inaccuracy, lack of clinician knowledge, prescribing errors, difficulty accessing resources, behavioral changes, and Medicare hospice criteria. → Challenges w/clinical diagnosis and care

BURDEN OF CARE

- Vatter, et al. [50] Areas impacted were relationship satisfaction, intimacy, communication, care responsibilities, emotional distancing, frustration, resentment, anger, sadness, fear of future. Three themes related to impacts experienced by CG: (1) altered relationship; (2) care partner challenges; and (3) acceptance and adjustment. → Barrier to EOL care
- Armstrong, et al. [54] Themes: (1) caregivers drive care, (2) DLB symptoms affect EOL for CG, (3) CG role, (4) death + post-death, and (5) supports for CG. DLB features that impacted CG included: fluctuations, hallucinations, delusions, REM, Autonomic symptoms, Physical limitations, falls. → EOL experience for CG
- Harding, et al. [29] Pt had worsening cognitive symptoms (70%), ability to do things (62%), well-being (57%) and changes to medication (26%). Carers had less support (55%) which adversely affected their mental health. → Lack of support + Mental health impacts

NEEDS & PRIORITIES (EDUCATION, SUPPORT, & RESEARCH)

- Killen, et al. [52] Half reported no information or support following diagnosis. Most wanted more information was about hallucinations, cognitive fluctuation, psychiatric symptoms, sleep, memory, tremor and medications (>60% all). 90% wanted information to increase awareness, coping and data on what has worked for others. → Educational needs of CG
- Stacy, et al. [27] Concerns identified: difficulty getting diagnosis, what to expect, strategies to cope, need for support, symptom management, physical care, managing meds and appointments, mental health, respite, communication → Challenges faced by CG
- Brown, et al. [49] Themes: (1) CG valued specialty support program, which (2) enabled better management of challenges with practical guidance, (3) provided emotional benefits, and (4) helped CG feel supported. → Nursing care support program
- Armstrong, et al. [54] Themes: (1) caregivers drive care, (2) DLB symptoms affect EOL for CG, (3) CG role, (4) death + post-death, and (5) supports for CG. DLB features that impacted CG included: fluctuations, hallucinations, delusions, REM, Autonomic symptoms, Physical limitations, falls. → Support needs for CG
- Armstrong, et al. [55] Themes: lack of knowledge about what to expect, end-of-life trajectory + symptoms, advance care planning, hospice, right-to-die, medications, end of life + death experience, and activities that enhanced end of life, lack of communication, difficulty predicting death. → Support needs at EOL
- Armstrong, et al. [26] Themes: Participants valued clinician time, diagnosis, education, symptom management, communication, and caring staff. Needs included education for Pt, CG, non-specialist clinicians and community providers, scheduling difficulties, CG support, financial concerns, advance care planning, local resources, and effective treatments for symptoms → Unmet needs and meaningful aspects of clinical care
- Armstrong, et al. [47] Themes: Research priorities for Pt and CG were DLB symptoms, therapies to prevent, cure, or delay progression, impact on daily function, quality of life, caregiving, and improving education. → Research priorities

EFFECT OF COVID LOCKDOWN ON SYMPTOMS

- Cagnin, et al. [58] Worsening of behavioral and psychological symptoms - 59.6% had worsening or onset of new BPSD, which was higher for DLB (63.8%). DLB Pt with BPSD had > neuropsychiatric symptoms vs. other groups. Worsening was most frequently reported for: irritability (40.2%), apathy (34.5%), agitation (30.7%), anxiety (29%), depression (25.1%), sleep disorder (24%), delusions and hallucinations (10%). BPSD was similar by CDR severity across diseases.
- Harding, et al. [29] Pt had worsening cognitive symptoms (70%), ability to do things (62%), well-being (57%) and changes to medication (26%). Carers had less support (55%) which adversely affected their mental health.

→ Worsening symptoms for pt and mental health for CG due to isolation during COVID

THEMATIC FINDINGS — PROFESSIONAL CAREGIVERS

NEEDS EDUCATION & AWARENESS

- Jackson, et al. [79] LBD is often under recognized and not diagnosed. Staff felt not well trained or equipped to provide specific care for LBD. Lack of staff awareness about benefits of obtaining a formal diagnosis. → Educational needs of paid CG

NOTES. REFERENCE NUMBER SHOWN AS [#].

Patient Experiences

- **THEMATIC FINDINGS — PEOPLE WITH LBD/DLB**
  - **SYMPTOMS**
    - **ALL SYMPTOMS**
      - Yumoto and Suwa [28] Changes in the daily life of people with DLB: Most common symptoms were cognitive decline, visual hallucinations, RBSD, and parkinsonism. Difficulties were due to symptoms Pt was aware and unaware of. Themes: bodily discomfort (trouble moving/doing), daily obstacles (forgetting, communicating), psychological pain, isolation, frustration, depression.
        - Diverse symptoms; Functional impacts; Lack of symptom awareness
      - van de Beek, et al. [42] Parkinsonism was the most frequent core feature in MCI-LB (69%). LB fared worse on neuropsychiatric symptoms, non-memory cognitive domains, and CG burden. LB group declined faster on attention
        - Prodromal symptoms
      - Larsson, et al. [51] Themes focus on: (1) disease impact, i.e., symptom experience and restricted activities; (2) self-perception and coping strategies; (3) importance of others. Patients reported wide range of symptoms and prioritized physical complaints over cognitive, in contrast to CG perspective in other studies.
        - Diverse symptoms; Functional impacts; Pt priority physical symptoms
      - Donaghy, et al. [43] Symptoms more common in DLB and MCI-LB: fluctuating attention/ concentration, rigidity/stiffness, shuffling, handwriting changes, drooling, frequent falls, posture changes, weak voice, REM sleep behavior disorder, spatial misperception, loss of smell, slowness, visual hallucinations.
        - Diverse symptoms
      - Galvin, et al. [56] Presenting symptom were cognitive (48%), motor (39%), or mixed 13%. Multiple visits (>6) and multiple doctors (3.3 ± 1.5) needed to establish LBD diagnosis; 31% took > 2 years. Initial diagnosis not LBD in 78%: PD, AD, FTD, MDD.
        - Presenting symptoms (CG perception)
    - **COGNITIVE**
      - Matterson, et al. [40] Themes: (1) symptoms of cognitive fluctuations were inconsistent; (2) both Pt and CG had sleep disturbances due to DLB. Poor sleep quality was a trigger for worsening cognitive fluctuations. Excessive daytime sleepiness was common.
        - Cognitive fluctuations; Impact on sleep
      - Harding, et al. [29] Pt had worsening cognitive symptoms (70%), ability to do things (62%), well-being (57%) and changes to medication (26%). Carers had less support (55%) which adversely affected their mental health.
        - Cognitive symptoms & functional impact
      - Bradshaw, et al. [45] DLB CG cited lapse in awareness or attention, lost ability to engage in meaningful cognitive or physical activity with short lived alterations in cognitive and functional abilities vs. AD CG reporting confusion and more persistent alterations
        - Cognitive fluctuations; Lack of awareness
    - **PSYCHIATRIC**
      - Yumoto and Suwa [41] Difficulties from hallucinations included feeling something strange is happening, discomfort, activity restrictions, inability to talk to family about it, and emotional distress. 70% did not use term hallucination.
        - Hallucination & Impact of hallucinations
      - Cagnin, et al. [58] Worsening of behavioral and psychological symptoms - 59.6% had worsening or onset of new BPSD, which was higher for DLB (63.8%). DLB Pt with BPSD had > neuropsychiatric symptoms vs. other groups. Worsening was most frequently reported for: irritability (40.2%), apathy (34.5%), agitation (30.7%), anxiety (29%), depression (25.1%), sleep disorder (24%), delusions and hallucinations (10%). BPSD was similar by CDR severity across diseases.
        - Behavioral and Psychiatric symptoms
      - Ballard, et al. [46] 93% DLB experienced at least one type of visual hallucination, and 56% had multiple, which was significantly more common than in the AD group.
        - Hallucinations
    - **SLEEP**
      - Kashihara, et al. [44] Sleep problems, vivid dreams, and nocturnal vocalization were more frequent in patients with PAF, IPD and DLB vs. controls. Difficulty falling asleep was similar to stroke controls. Awakenings higher in iPD group. Daytime sleepiness was higher in iPD and DLB.
        - Sleep problemns
    - **SPEECH**
      - Volkmer, et al. [77] Themes for LBD group related to aphasia: (1) communication difficulties a key problem, (2) feeling of isolation due to speech issues (worse for CG than for Pt), (3) doing anything that helps, (4) low access to speech and language therapy.
    - **URINARY**
      - Coindreau, et al. [71] 100% had over active bladder, stress incontinence (94%), detrusor overactivity (93%), bladder pressure elevation (79%), voiding difficulties (16%), constipation (92%), and fecal incontinence (44%)
        - Bladder & bowel symptoms
  - **IMPACT OF DISEASE**
    - Yumoto and Suwa [28] Changes in the daily life of people with DLB: Most common symptoms were cognitive decline, visual hallucinations, RBSD, and parkinsonism. Difficulties were due to symptoms Pt was aware and unaware of. Themes: bodily discomfort (trouble moving/doing), daily obstacles (forgetting, communicating), psychological pain, isolation, frustration, depression.
      - Physical, Psychological & Functional impact
    - Volkmer, et al. [77] Themes for LBD group related to aphasia: (1) communication difficulties a key problem, (2) feeling of isolation due to speech issues (worse for CG than for Pt), (3) doing anything that helps, (4) low access to speech and language therapy.
      - Communication; Isolation, Coping
    - Yumoto and Suwa [41] Difficulties from hallucinations included feeling something strange is happening, discomfort, activity restrictions, inability to talk to family about it, and emotional distress. 70% did not use term hallucination.
      - Psychosocial impact of hallucination
    - Larsson, et al. [51] Themes focus on: (1) disease impact, i.e., symptom experience and restricted activities; (2) self-perception and coping strategies; (3) importance of others. Patients reported wide range of symptoms and prioritized physical complaints over cognitive, in contrast to CG perspective in other studies.
      - Physical, Psychological & Functional impact
    - Bradshaw, et al. [45] DLB CG cited lapse in awareness or attention, lost ability to engage in meaningful cognitive or physical activity with short lived alterations in cognitive and functional abilities vs. AD CG reporting confusion and more persistent alterations
      - Functional impact
  - **NEEDS & PRIORITIES**
    - Watermeyer, et al. [53] Goals for cognitive rehab were: use technology (email), engage in leisure activities; manage medications; manage self (ADL), orient self to time/place, remember where things are, have social life, manage anxiety.
      - Goals for cognitive rehab
    - Armstrong, et al. [26] Themes: Participants valued clinician time, diagnosis, education, symptom management, communication, and caring staff. Needs included education for Pt, CG, non-specialist clinicians and community providers, scheduling difficulties, CG support, financial concerns, advance care planning, local resources, and effective treatments for symptoms
      - Healthcare priorities; Resources; Advance care planning
    - Armstrong, et al. [47] Themes: Research priorities for Pt and CG were DLB symptoms, therapies to prevent, cure, or delay progression, impact on daily function, quality of life, caregiving, and improving education.
      - Research priorities

Notes. Reference number shown as [#].
